# Gamma Radiation Shielding Efficiency of Cross-Linked Polystyrene-b-Polyethyleneglycol Block Copolymer Nanocomposites Doped Arsenic (III) Oxide and Boron Nitride Nanoparticles

**DOI:** 10.3390/polym17243330

**Published:** 2025-12-17

**Authors:** Bülend Ortaç, Taylan Baskan, Saliha Mutlu, Sevil Savaskan Yilmaz, Ahmet Hakan Yilmaz

**Affiliations:** 1 UNAM-National Nanotechnology Research Center and Institute of Materials Science and Nanotechnology, Bilkent University, Ankara 06800, Turkey; 2 Department of Physics, Faculty of Sciences, Karadeniz Technical University, University Avenue, Trabzon 61080, Turkey; 3 Department of Chemistry, Faculty of Sciences, Karadeniz Technical University, University Avenue, Trabzon 61080, Turkeysevily@ktu.edu.tr (S.S.Y.)

**Keywords:** radiation shielding, PS-PEG block copolymers, arsenic trioxide, boron nitride, gamma attenuation, mass attenuation coefficient, linear attenuation coefficient, the half value layer, the tenth value layer, the mean free path, the radiation protection efficiency

## Abstract

In recent years, polymer-based hybrid nanocomposites have emerged as promising alternatives to traditional heavy metal shields due to their low density, flexibility, and environmental safety. In this study, the synthesis of PS-PEG copolymers and the gamma radiation-shielding properties of PS-PEG/As_2_O_3_, PS-PEG/BN, and PS-PEG/As_2_O_3_/BN nanocomposites with different compositions are investigated. The goal is to find the optimal nanocomposite composition for gamma radiation shielding and dosimetry. Therefore, the mass attenuation coefficient (MAC), linear attenuation coefficient (LAC), half-value layer (HVL), tenth-value layer (TVL), effective atomic number, mean free path (MFP), radiation shielding efficiency (RPE), electron density, and specific gamma-ray constant were presented. Gamma rays emitted by the Eu source were detected by a high-purity germanium (HPGe) detector device. GammaVision was used to analyze the given data. Photon energy was in the vicinity of 121.8–1408.0 keV. The MAC values in XCOM simulation tools were used to compute. Gamma-shielding efficiency was increased by an increased number of NPs at a smaller photon energy. At 121.8 keV, the HVL of a composite with 70 wt% As_2_O_3_ NPs is 2.00 cm, which is comparable to the HVL of lead (0.56 cm) at the same energy level. Due to the increasing need for lightweight, flexible, and lead-free shielding materials, PS-b-PEG copolymer-based nanocomposites reinforced with arsenic oxide and BN NPs will be materials of significant interest for next-generation radiation protection applications.

## 1. Introduction

Radiation, in addition to occupying an important place in life, also has some negative effects. While radioactive materials in various areas are used to improve human health, they also pose a risk to human health. On the other hand, science utilizes radioactivity to meet the increasing energy demands resulting from the development of technology. Nuclear power plants, in particular, stand out in this area. Nuclear power plants use chain reactions to produce energy. However, the safe transportation and storage of these radioactive materials, which are the fuel used at the end of the process, emerges as a problem. Again, various shielding materials are needed during the safe transportation, storage, and use of these materials, which can have very negative effects on human health. Cosmic rays, another source of radiation, and the secondary radiation products that form that also require protection in terms of the occurrence of significant health problems today and in the future. Space technologies and some aircraft technologies are working on producing shielding materials within this framework. The impact of ionizing radiation on humans and the environment is increasing [1,2,3,4,5].

Technological procedures that involve radiation exposure possess future effects that are harmful to health, despite being indispensable because of their advantages in diagnostic and therapeutic applications in the medical domain. Patients subjected to CT and X-ray imaging scans are exposed to certain doses of gamma radiation, which may be transmitted via tissues and cause ionization in atoms, resulting in damage to their organs [6].

Similarly, during cancer treatments (therapy), patients are subjected to gamma radiation in significant doses targeting the cancerous cells, while simultaneously posing health risks to the surrounding organs and tissues. Extreme subjection to gamma rays enhanced the tendency of breast cancer, thyroid cancer, and leukemia [7].

In order to use the positive effects of radiation, the need to significantly reduce its negative effects, as well as the other properties of the material, is gaining importance day by day. The costs of radiation protection materials, their thermal resistance properties, and flexible structures for usable purposes stand out as important factors in technology research. This has increased the importance given to nanostructured materials. It is observed that nanomaterials improve other properties, as well as increasing radiation resistance [1,2,3,4,5,8].

On the other hand, the use of composite structures is increasing to increase the effects of nanomaterials. Composite structures are useful in improving the properties of the materials that form them. In addition, providing good thermal resistance and flexibility to a material, in addition to good shielding properties, does not require that all of its features be top-notch. It is more accessible, costlier, and effective for the composite to be adequate for its purpose. For these reasons, the use of composite materials in nuclear technologies and studies in this field is published [8,9,10,11,12,13,14,15,16]. Polymer composites reinforced with inorganic micro- and nano-NPs have recently come into use as alternative materials to lead in the field of radiation shielding, with major advantages such as flexibility, chemical stability, lightness, low cost, corrosion resistance, physical, thermal, and radiation properties, in addition to their lightness [8,14,15,17,18,19,20,21,22,23,24]. This study investigates the shielding capabilities of several PS-PEG copolymer systems, namely RPA1, RPA2, and RPA3, enhanced with As_2_O_3_ and boron nitride (BN) NPs through a detailed analysis of key attenuation metrics. Mona M. Gouda and co-workers investigated the radiation attenuation capability and particle size effect of PS-PbO, a novel polymer nanocomposite containing two different nanosized lead oxide nanoparticles (NPs) (PbO-A and PbO-B) in addition to the bulk size (PbO-K). Gamma-ray energies ranging from 0.06 to 1.3 MeV (from sources such as ^241^Am, ^133^Ba, ^137^Cs, and ^60^Co) were considered in the study. Experimental values revealed that the gamma-ray shielding performance of PS-PbO composites was improved in both low- and high-energy ranges by increasing the PbO concentration. This is consistent with the theory that increasing the Pb percentage increases the number of electrons in the composite and therefore increases the probability of γ-ray interaction [25]. Despite the well-known harmful nature of arsenic oxide, its addition to the current study exclusively stems from its relatively high atomic number of Z = 33. This greatly increases the probability of photoelectric absorption and the Compton scattering effect of photons. Crucially, the As_2_O_3_ NPs used within the cross-linked PS-PEG matrix forms a solid polymer–inorganic matrix that greatly reduces the potential leaching of As_2_O_3_ NPs into the environment. This particular phenomenon has been well documented within the context of other polymer–NPs composites that serve as traps and have very low solubility levels at ambient conditions with relative phases of arsenic that are well entrapped. The nanocomposites developed in this study are intended for use in closed-system radiation-shielding applications (e.g., medical device housings, detector casings), where environmental exposure is inherently limited. As_2_O_3_ is a network former in which AsO_3_ pyramidal units are discovered. Glasses containing heavy metal oxides, such as As_2_O_3_, have sparked a lot of interest in recent years due to their high mechanical, radiation, and nonlinear optical characteristics [26]. Studies on protection against ionizing radiation by Afaf M. Babeer and others have found improved performance with the addition of As_2_O_3_. An increase in mass attenuation values was observed as the As_2_O_3_ content was increased from 0 to 16 mol%. Furthermore, HVL measurements of the studied As_2_O_3_-NiO-Na_2_O-B_2_O_3_ glass samples were found to exhibit relatively lower HVL values compared to the HVL of certain incumbent materials commonly used for protection against ionizing radiation [27].

This study brings forth the application of PS-PEG-based hybrid nanocomposites incorporated with As_2_O_3_ and BN NPs together for the first time and provides evidence of their combined gamma-ray attenuation coefficient values. Additionally, we have incorporated the crucial fact that the study combined simulations with experiments using the HPGe detector and XCOM simulation results that led the way in developing air-cooled and lead-free shield materials. The originality of this study rests with the first-time material development and assessment of the gamma attenuation efficiency of PS-PEG hybrid nanocomposites reinforced with As_2_O_3_/BN NPs. Indeed, the combined effects of the As_2_O_3_ and BN NPs increase the gamma attenuation efficiency of the material substantially while preserving the flexibility and low density of the material. Additionally, this study introduces the first complete structure/attenuation relationship experimentally measured with the aid of XCOM simulation on the gamma attenuation efficiency of the PS-PEG/As_2_O_3_/BN material system.

One of the major strengths of the PS-PEG/BN copolymers is found in their biological and physical characteristics. RP2ABN-2, for example, possesses a density of about 1.92 g/cm^3^, several orders of magnitude lower than that of lead (11.34 g/cm^3^) [28] and that of tungsten (19.25 g/cm^3^) [29], thus leading to significantly lighter shielding solutions. In addition, whereas Pb is notoriously toxic and a source of environmental risks, and W, albeit safer, still presents biocompatibility issues, the PS-PEG/BN composites are naturally biocompatible. As a consequence, these are perfect contenders for uses like wearable protection gear and medical diagnosis, for which safety and comfort are of critical importance. Lead-based substances are said to be the gold standard for radiation shielding due to their high density and superior attenuation. For all that, the toxicity of lead, together with its high weight, is a serious drawback for some critical uses, e.g., for biocompatible shielding or for portable shields. Tungsten, being less toxic but equally effective, is, however, extremely heavy. For that matter, the development of novel composites for shielding that can be a compromise between performance of attenuation, safety factors, and weight considerations assumes a priority. In this respect, PS-PEG/BN copolymer composites are a promising candidate. In the present work, the authors study the shielding of gamma rays for a series of selected PS-PEG/BN nanocomposites relative to that of lead and tungsten [29].

Saberi Rise and the others present a new, fast, simple, and low-cost synthesis of bismuth powders using aluminum foil as a gamma-shielding alternative to lead and cement-based materials. Flexible BiPMMA composites with 40, 50, and 60 wt% of bismuth were subsequently prepared, and their gamma-shielding properties were investigated [9].

Lead-free and high-atomic-number materials, which are integrated with a polymer matrix (for structural support), have attracted significant attention [10,11].

The use of shields in radiation applications is a widely accepted form of protecting gadgets, human and non-human components of the biosphere from non-deliberate exposure to ionizing radiation. The cost of accidental exposure to radiation is invaluable, ranging from biological effects such as gene reconstruction leading to tissue and organ malfunctioning to diseases such as cancer [30].

The effectiveness of a shielding material hinges on its capacity to attenuate or absorb gamma-ray energy. Evaluating the effectiveness of materials against gamma radiation necessitates the examination of several essential parameters. One important parameter is the attenuation coefficient, which gauges a material’s ability to diminish the intensity of gamma rays. This coefficient quantifies the probability of radiation interacting with matter over a given distance and is affected by factors such as incident photon intensity, absorber’s atomic number, and material density. In recent years, numerous researchers have engaged in extensive studies regarding the attenuation coefficients of diverse materials. These studies encompass a wide range of substances, including bricks, ores, glasses, brow, organic compounds, and minerals [12,13,14,15,16].

Nanostructured polymeric composites have recently attracted considerable attention as potential new materials, replacing conventional high-Z materials due to their lightness, mechanical hardness, and better thermal and chemical durability. As part of this study, we have found that the addition of certain materials like As_2_O_3_ and BN results in stronger gamma-ray attenuation capabilities. The results clearly show that nanocomposite materials possess better gamma-ray attenuation capabilities than their respective homogeneously combined materials. Some of the materials have shown remarkable gamma-ray shielding efficiency that could lead to the reduction in material thickness with successful protection. Most importantly, As_2_O_3_ and BN together make the highest contribution toward the enhancement of gamma-ray attenuation behavior. Already, it has been noticed that polymeric composites have the disadvantage of higher thickness materials required at an insignificant cost when compared with Pb and W materials; despite this limitation, the lighter density makes these materials very attractive. As_2_O_3_ and As_2_O_3_/BN NPs together make the highest shielded performance. RP3A-3 and RP3ABN-1 are very efficient materials with relatively low HVL values and higher $\mu_{L}$ values. Among the prepared copolymers, RP2ABN-2, RP2ABN-3, and RP2A-3 provided the best values for most of the major attenuation parameters. $\mu_{L}$ took the values of 0.899 cm^−1^, 0.815 cm^−1^, and 0.761 cm^−1^ at 121.78 keV for RP1A-3, RP3A-3, and RP3ABN-1 nanocomposites, respectively; thus, these values are close to the values of Pb (1.24 cm^−1^) [31] and W (1.10 cm^−1^) [31]. Also, these values are close to the values of Pb (1.12 cm^−1^) and tungsten carbide (WC) (1.23 cm^−1^) at 344 keV [32]. The HVL was found to be 0.85 cm and 0.97 cm for RP3A-3 and RP2ABN-2, respectively, while 0.56 cm and 0.63 cm were found for Pb [31] and W [31], respectively. The HVL values of Pb and WC are 0.61 cm and 0.56 cm at 344 keV, respectively [32]. Although the nanokompozitler require greater thickness for equivalent radiation protection, they achieve a usable advantage through lower density.

This study presents a detailed comparison between the $\mu_{m}$ and $\mu_{L}$ experimental data of nanocomposites containing different amounts of As_2_O_3_ and BN NPs-based PS-PEG copolymers and the shielding performance of selected metal oxides (Al_2_O_3_, MnO_2_, CuO, MgO) across photon energies ranging from 121.78 keV to 1408.01 keV. Dahinde et al. investigated a comparative analysis of radiation attenuation properties of Al_2_O_3_, MnO_2_, CuO, and MgO at various photon energies. At 122 keV, experimentally, the HVL values are 0.5821 cm for Al_2_O_3_, 0.522 cm for MnO_2_, 1.7163 cm for CuO, and 1.0202 cm for MgO. Theoretical results closely follow, with Al_2_O_3_ at 0.5901 cm, MnO_2_ at 0.5364 cm, CuO at 1.7415 cm, and MgO at 1.0353 cm. Among the oxides, CuO shows the highest HVL, indicating that it is the least effective in attenuating low-energy photons. In contrast, MnO_2_ demonstrates the lowest HVL, highlighting its superior shielding performance at this energy level. At 356 keV, experimentally, Al_2_O_3_ exhibits an HVL of 0.3787 cm, MnO_2_ of 0.3492 cm, CuO of 0.6499 cm, and MgO of 0.4774 cm. The theoretical values are similarly consistent, with Al_2_O_3_ at 0.3907 cm, MnO_2_ at 0.3564 cm, CuO at 0.631 cm, and MgO at 0.4925 cm. As expected, HVL values decrease with increasing energy. However, CuO continues to have the highest HVL, confirming its relatively poor shielding performance, while MnO_2_ remains the most effective. At 1330 keV, the HVL values drop further at higher energy: Al_2_O_3_ records 0.2083 cm, MnO_2_ 0.1692 cm, CuO 0.2587 cm, and MgO 0.2412 cm experimentally. The theoretical results align with values of 0.1953 cm for Al_2_O_3_, 0.1764 cm for MnO_2_, 0.2776 cm for CuO, and 0.2311 cm for MgO. The HVL values at this high energy are significantly lower due to increased photon penetration [33].

Consequently, the purpose is to consider the protection performance of nanocomposites in a broader context than traditional oxide materials and to interpret the observed trends. Comparing the protection ability of a single material to that of a single mass, the MAC was found to be high for RP2A-2, RP3A-3, RP3ABN-1, RP3ABN-2, and RP2ABN-2.

## 2. Materials and Methods

Six nanocomposite systems were examined over a range of photon energies from 121.78, 344.28, 778.9, 964.08, 1085.87, 1112.07, to 1408.01 keV. The attenuation coefficients were determined experimentally or calculated through duly calibrated computational methods, and the result was given in tabular format for comparative analysis.

### 2.1. Synthesis and Characterization

Our research group carried out synthesis, characterization, and detailed analysis of PS-b-PEG in the laboratory. The molecular weight of the cross-linking PEG polymers used in the synthesis of cross-linked PS-b-PEG block copolymers (1000 gmol^−1^, 1500 gmol^−1^_,_ and 10,000 gmol^−1^) was cross-linked. The characteristic peaks of PEG (1000-1500-10,000) cross-linkers and PS-b-PEG copolymers were examined in detail using FTIR and NMR methods [14,15,16,17,18,19,20].

### 2.2. Characterization of Polymer-Nanostructured-Particle-Based Nanocomposites

#### 2.2.1. Thermogravimetric Analysis (TGA)

The degradation temperatures and degradation percentages of PS-PEG/As_2_O_3_, PEG/BN, and PS-PEG/As_2_O_3_/BN nanocomposites, Seiko II Exstar 6000 (Seiko Instruments Inc., Chiba, Japan) were determined with the TGA/DTA/DTG analyzer system.

#### 2.2.2. Scanning Electron Microscopy (SEM) with Energy Dispersive X-Ray Analysis (EDX) Examinations

To examine the surface morphology of the nanocomposites, SEM analyses were performed using a Quanta 200 FEG-SEM (field emission gun (FEG)-scanning electron microscope (SEM)) (FEI Company, Hillsboro, OR, USA). Before SEM analyses, the nanocomposites were fixed on a carbon tape and coated with a 10 nm Au/Pd alloy (with PECS-682) to prevent the electron charging effect. Also, energy dispersive X-ray (EDX) analysis of the nanocomposites was performed.

#### 2.2.3. Transmission Electron Microscope (TEM) Analyses

The structural analyses of polymer–polymer-nanostructured-particle-based nanocomposites were carried out using a transmission electron microscope (TEM) from FEI Company(FEI-Tecnai G2F30, Hillsboro, OR, USA) system equipped with an EDX. A scanning transmission electron microscope (STEM)-EDX was used to examine As_2_O_3_ and BN NPs on polymer nanostructures. TEM samples were also prepared by fixing nanomaterials onto carbon-coated TEM grids.

#### 2.2.4. X-Ray Diffraction (XRD) Analysis 

XRD measurements were taken with the PANalytical X-Pert Powder device located at the Karadeniz Technical University Central Research Center. The device sends X-rays to a sample holder with a Cu Kα (1.54 Angstrom wavelength) X-ray source. The sample holder is a disk where 1 mg of the sample to be measured is spread evenly over a central region. This disk can be rotated 5–140° in the device we use. Thus, the sample is exposed to X-rays at angles between 5 and 140°, and the XRD device scans the regions that will give the characteristics of the structure. The X-Ray Diffraction method (XRD) is based on the principle that every material with a crystal structure diffracts X-rays in a characteristic pattern depending on its unique atomic arrangement. These diffraction patterns for each crystal or phase define that crystal like a fingerprint.

### 2.3. Preparation of the Nanocomposites

**Scheme 1:**
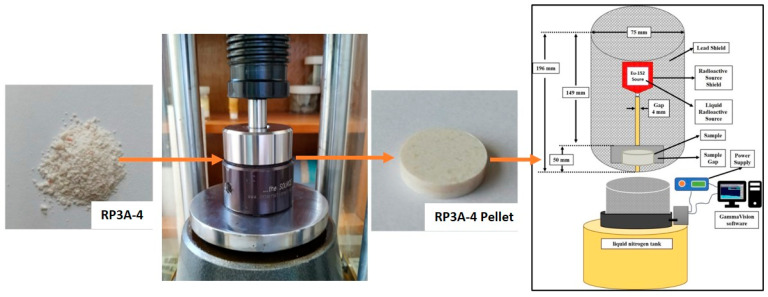
Pellet preparation from nanocomposite powder by hydraulic press (MTI Corporation, Richmond, CA, USA) and picture of an HPGe detector for gamma radiation attenuation investigations.

Table 1 shows the composition of nanocomposites. The nanocomposite pellets were prepared by pressing at room temperature using a hydraulic press with a 10-ton strength mold under 10 MPa pressure for 15–25 min. The mold diameter of 13 mm is the diameter of the pellets. Scheme 1 shows some nanocomposite powder mixtures, the pellet preparation hydraulic press (MTI Corporation), and an example of the prepared pellet.

### 2.4. The Gamma Radiation Investigations of the Naonocomposites

#### Determination of Radiation Shielding Effectiveness

The tested composites were denoted by combinations such as RPA1-RP1A-2, RPA2-RP2A-3, and RPA3-RP3A-2, including BN-modified variants (e.g., RP1ABN-3, RP2ABN-2, RP3ABN-3). The HVL measurements were performed at photon energies of 121.78, 344.28, 778.9, 964.08, 1085.87, 1112.07, and 1408.01 keV. Values were obtained using a standard gamma attenuation experimental setup and validated by repeated measurements. Detector specifications: threshold is approximately 40 keV, bias voltage is 3500 volts, and gain is 0.5489.

The experimental system shown in Scheme 1 was established to determine the gamma-shielding properties of composite pellets. The HPGe detector system (25% efficiency) consists of a body containing the germanium block and the counting system, a tank containing liquid nitrogen at −196 °C, a power supply to which the 3500 Volt power required for the counting system is applied, and a computer software (GammaVision Software V6) that manages all these.

The gamma radiation source is a radioactive source known as ^152^Eu, which emits more than 10% gamma at various energy values. These energy values are 121.8, 344.3, 778.9, 964.1, 1085.9, 1112.1, and 1408.0 keV. The ^152^Eu source we used to be manufactured on 1 October 2010 had an activity of 5.12 g and 98.50 μCi. The activity of the source at the time of the measurements was approximately 48.17 μCi.

The counting is performed by converting the photons falling on the detector into electrical signals. For this reason, a geometry (Scheme 1) is created at the top of the system. This geometry is placed inside a lead shield. There is a 4 mm diameter gap in the middle of the shield. High-energy photons coming from the source pass through this gap and reach the sample chamber. If there is no sample in this section, the photons reach the detector and are counted by the detector. This count is called an empty count ($I_{0}$). Then, the sample is placed, and in this case, some of the photons are absorbed by the sample. The detector counts the photons (I) that pass without being absorbed. This count is called the absorption count. Three measurements were taken for each composite pellet. Both counting processes are given as a count–energy graph by the GammaVision software. Peak analysis is performed on this graph, and the values for peak areas (I) and ($I_{0}$) are given. The duration of the measurements and the potential value given by the power supply are controlled by GammaVision software. The power supply applies a potential of 3500 V to the germanium crystal. The duration of each measurement is 1200 s. The radiation efficiencies of the nanocomposites can be calculated using the peak areas obtained. To measure this efficiency, $\mu_{L}$ (LAC), $\mu_{m}$ (MAC), HVL, TVL, MFP, and RPE coefficients were calculated.

The $\mu_{L}$ is a method used to study the attenuation shielding capabilities of materials against gamma radiation. Table S2 shows the experimental and theoretical (XCOM) $\mu_{m}$ values of the nanocomposites. Figure 5 shows $\mu_{m}$ (graphs of PS-PEG/As_2_O_3_, PS-PEG/BN, and PS-PEG/As_2_O_3_/BN nanocomposites).

MAC, the shielding ability (gamma attenuation) of any material in good geometrical conditions, depends on the density of the absorber and the LAC. MAC is defined as the probability of interaction between the mass per unit area of the material and the incident gamma photons, usually expressed in cm^2^/g. MAC and LAC work similarly and are related by the material density $\rho \;(g/{cm}^{3})$. The mathematical equations used in the calculations are arranged in a comprehensive Table 2 to facilitate easy reference and comparison.

**Table 2:** The equations used in this study.

Formula Name	Equations	Equation Numbers	Symbol Descriptions	Ref.
Lambert–Beer	$I=I_{0}\;e^{{-µ}_{L}x}$	(1)	*I_0_*, the incident gamma intensity; *I*, Lineer attenuation coefficient $(\mathrm{LAC});\;\mu_{L}$, the attenuation shielding capabilities; x, the thickness of the nanocomposite	[8]
The mass attenuation coefficient (MAC)	$\mu_{m}=\frac{\mu_{L}}{\rho }\;({cm}^{2}/g)$	(2)	$\mu_{m}$$\;(\mathrm{MAC}),\;\mu_{L}$ = Lineer attenuation coefficient (LAC), ρ = the material density	[8]
Lambert–Beer	$I=I_{0}\;e^{{-µ}_{m}\times \frac{m}{A}}$	(3)	m: The mass of the pellet, A: the surface area	[8]
The half-value layer (HVL) and Tenth-value layer (TVL)	$HVL=\frac{\ln2}{\mu_{L}}\;(cm)$ $TVL=\frac{\ln10}{\mu_{L}}$ (cm)	(4)	HVL: half-value layer TVL = tenth-value layer	[8]
Mean free path (MFP)	$MFP=\frac{1}{\mu_{L}}\;({cm}^{-1})$	(5)	MFP = The mean free path	[8]
Radiation shielding efficiency (RPE)	$RPE\;(\%)=\left(1-\frac{I}{I_{0}}\right)\times 100$	(6)	RPE: Radiation shielding efficiency	[8]

## 3. Results

### 3.1. The Thermal Gravimetry (TG)/Differential Thermogravimetric (DTG)/Differential Thermal (DTA) Analysis

**Figure 1:**
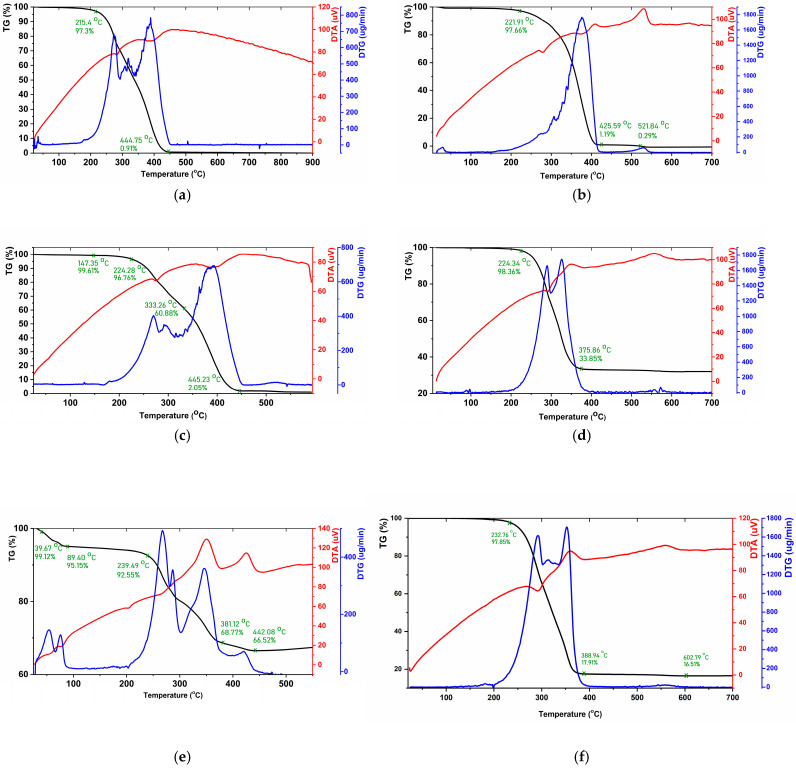
TGA thermograms of RP1A-1 (PS-b-PEG1000-50 wt% As_2_O_3_-50 wt%) (**a**), RP2A-1 (PS-b-PEG1500-50 wt% As_2_O_3_-50 wt%) (**b**), RP3A-1 (PS-b-PEG10 000-50 wt% As_2_O_3_-50 wt%) (**c**), RP1ABN-1 (PS-b-PEG1000-15 wt% BN-15 wt% As_2_O_3_-70 wt%) (**d**), RP2ABN-1 (PS-b-PEG1500-15 wt% BN-15 wt% As_2_O_3_-70 wt%) (**e**), RP3ABN-1 (PS-b-PEG10 000-15 wt% BN-15 wt% As_2_O_3_-70 wt%) (**f**).

Figure 1a–f show TG (% weight loss), DTG (decomposition rate), and DTA (thermal event) profiles for the nanocomposites. TGA results of the nanocomposites for a variety of nanocomposites are presented in Table S1, with a particular emphasis on the three phases of thermal degradation. The temperature (t °C) at which degradation occurs and the mass wt% that remains after each stage are visible.

TG/DTG/DTA curves of the RP1A-1 nanocomposite, composed of PS-b-PEG1000 (50 wt%) and As_2_O_3_ NPs (50 wt%), are shown in Figure 1a. The thermal behavior of the RP1A-1 can be summarized in three stages: in the first stage, the initial loss of 2.7 wt% at 215.4 °C is due to the loss of humidity in the nanocomposite, resulting in 97.3 wt% mass retention. In the second stage, around 444.75 °C, the main degradation of the polymer components (PS-b-PEG and As_2_O_3_ NPs occurs, leaving only 0.91 wt% mass retention). RP1A-1 degrades to 99.09 wt% at the highest second-stage temperature (445 °C), demonstrating the superior thermal stability of the main chain. No further thermal events were observed in the third stage.

The initial decomposition temperature of RP2A-1 (PS-b-PEG1500-50 wt% As_2_O_3_-50 wt%) is 221.91 °C (remaining amount 97.66 wt%), and the primary weight loss of 2.34 wt% is due to moisture loss (Figure 1b). The secondary decomposition temperature of RP2A-1 was recorded at 425.59 °C, with a weight loss of 1.19%.

RP3A-1 (PS-b-PEG10 000-50 wt% As_2_O_3_-50 wt%) showed the decompositions at 147.35 °C, 224.34 °C, with weight losses 0.38 wt% and 3.24 wt% (Figure 1c). These decompositions usually mean losing moisture. The third decomposition shows with weight losses 39.12 wt% at 333.26 °C. The fourth decomposition was seen at 445.23 °C, causing a further weight loss of 97.91 wt% (remaining amount 2.05 wt%). A residual substance of 0.39 wt% remained after thermally induced degradation (Figure 1c). The RP1ABN-1 nanocomposite exhibited a minor decomposition at 224.34 °C, with a weight loss of 1.64 wt% (remain 98.36%). This indicates moisture loss. Another decomposition peak was detected as 66.15 wt% loss at 375.86 °C, but no other weight loss was recorded after this step. A considerable residue of 33.85 wt% was detected, which can be attributed to the formation of thermally stable inorganic residues such as BN and As_2_O_3_ (Figure 1d). The mass loss of the RP2ABN-1 composite, starting at 39.67 °C, continues until 86 °C and 239.49 °C. The 0.88 wt%, 4.85 wt%, and 7.45 wt% losses corresponding to these three temperatures indicate that moisture has been removed (Figure 1e). The degradations at 381.12 °C and 442.08 °C are 31.23 wt% and 33.48 wt%, and are attributed to the degradation of PS-b-PEG copolymer into the thermally stable region of BN and As_2_O_3_ inorganic residues.

Figure 1f shows the TG/DTG/DTA curves of the RP3ABN-1 nanocomposite, composed of PS-b-PEG10,000 (15 wt%), BN (15 wt%), and As_2_O_3_ NPs (70 wt%). RP3ABN-1 is a BN/As_2_O_3_-rich nanocomposite with long-chain PEG, and the resulting thermal curve gives the impact of high inorganic loading alongside that of PEG10,000. It was at 232.76 °C that major decomposition of the polymer constituents (PEG and PS) occurred, with a residual mass of 97.85 wt%. Until 388.94 °C, widespread mass loss was complete, leaving behind mostly inorganic residues; residual mass was 17.91 wt%. At 602.79 °C, the sample was in a thermal plateau, with a residual mass of 16.51 wt%.

RP3A-1 and RP1ABN-1 have higher initial thermal resistance because they keep more than 98 wt% of their mass during the first stage of degradation. The second degradation stage ranges from 239 °C to 445 °C. It is the same as breaking down the copolymer chain. The RP2ABN-1 (92.55 wt%) and RP3A-1 (60.88 wt%) nanocomposites have a lot of residues because they have polymer and inorganic NPs in them. RP1A-1 (0.91 wt%) and RP2A-1 (1.19 wt%), on the other hand, are almost completely broken down, which means they are less resistant to heat at this point. The third degradation stage is observed only in some composites, between 442 °C and 603 °C. It involves the decomposition of polymer and inorganic residues. RP2ABN-1 (66.52 wt%) and RP3ABN-1 (16.51 wt%) show that there are a lot of leftovers because of the carbon filler. RP2A-1 and RP3A-1 also have small amounts of third-stage residue. RP2ABN-1 has a lot of inorganic material in it, which makes it very stable at high temperatures. This makes it good for use in high-temperature situations.

The BN-modified samples (RP1ABN-1, RP2ABN-1, RP3ABN-1) always did better than the BN in keeping their mass, showing that BN is a big part of how well things resist heat. RP2ABN-1, which exhibits the highest degradation resistance, stands out with its high residual mass in both the second and third stages. RP3ABN-1 exhibits both heat resistance and good thermal thresholds with a reasonable residual mass. The high-stability RP2ABN-1 nanocomposite retains the most residue even after all three stages. It has excellent thermal stability due to its BN or inorganic content. It is ideal for high-temperature or flame-retardant applications. RP3A-1 and RP3ABN-1 retain moderate mass in the second and third stages. They exhibit balanced degradation, making them suitable for applications requiring both stability and partial decomposition. RP2ABN-1 and RP3A-1 nanocomposites can be used as insulation or flame-retardant materials. TGA data show that the composites stay stable in terms of structure and temperature up to the temperatures that are common in medical imaging rooms or aerospace cabins. This means that As-containing species are not likely to evaporate when the materials are in use.

### 3.2. SEM, TEM, and EDX Characterization

Morphology primarily controls how efficiently the high-Z NPs intercept and attenuate photons inside the polymer matrix. In our nanocomposites, TEM and SEM images show that the BN and As_2_O_3_ NPs are (i) finely dispersed in the PS-PEG matrix, (ii) largely free of micron-scale agglomerates, and (iii) well embedded within a relatively low-porosity microstructure. Such a morphology is known to enhance gamma-ray shielding because it maximizes the effective interfacial area between the incident photons and the high-Z phases, leading to a higher effective MAC at a given overall filler loading. Several studies on polymer–metal oxide nanocomposites (e.g., PbO-, Se_2_O_3_-, and As_2_O_3_-filled polymers [8,34,35] have reported that uniform nanoscale dispersion and strong interfacial adhesion yield higher MAC, lower HVL, and improved radiation protection efficiency compared with systems where the same NPs are present as large aggregates or poorly bonded clusters, even at similar weight fractions. In contrast, pronounced agglomeration and voids introduce low-density regions and “shadow zones” that are weakly interacting with photons; this effectively reduces the path length of photons inside the dense phase and can even increase buildup factors due to multiple scattering in the polymer-rich regions. In our composites, the TEM (Figures S4–S6) and their statements/SEM micrographs indicate that BN platelets and As_2_O_3_ NPs domains form a continuous attenuation network rather than isolated regions. For the BN-rich formulations, the partial orientation of plate-like BN particles, frequently reported for hexagonal BN/polymer systems, contributes to both mechanical integrity and radiation shielding. Platelets aligned preferentially in the plane of the film increase the probability of photon interaction along the in-plane direction, which is consistent with the experimentally observed increase in MAC and reduction in HVL for samples with finer, more homogeneous BN dispersion. Similar structure–property relationships have been documented for h-BN/epoxy and h-BN/PMMA systems, where improved exfoliation and dispersion of BN sheets are directly correlated with higher photon attenuation coefficients and reduced MFP at diagnostic and therapeutic energies. Regarding the As_2_O_3_-bearing samples, the TEM images show that arsenic oxide NPs with dimensions on the order of tens of nanometers are dispersed throughout the PS-PEG matrix. This specific structure effectively brings the arsenic-rich elements into the sample volume throughout the material, which significantly increases the probability of photon absorption by the arsenic-rich zones and results in the enhancement of the calculated MAC and LAC of the nanocomposite. Overall, the TEM/SEM analysis confirms a clear link between morphology and radiation properties in the nanocomposites: (i) nanoscale dispersion and good wetting of BN and As_2_O_3_ NPs by the polymer phase enhance MAC and reduce HVL/TVL; (ii) minimization of voids and large agglomerates avoids low-density channels that would otherwise degrade shielding; and (iii) for plate-like BN NPs, partial orientation and formation of a quasi-continuous filler network further improve photon attenuation. These trends are in line with previous reports on polymer-based gamma-ray shields, where control of filler morphology (particle size, distribution, interfacial adhesion, and network formation) is identified as a key design parameter to optimize the radiation response of nanocomposite systems [25]. As_2_O_3_ is toxic on its own, but in our nanocomposites, it is not used as a free powder. It is trapped as a high-Z inorganic phase in a chemically cross-linked PS-b-PEG matrix. SEM, TEM, and attenuation analyses show that the NPs are spread out throughout the bulk and that the composites are dense, with little open porosity. This makes it very unlikely that particles will be released or come into direct contact with tissue during normal use. Figure S1a,b show SEM images (c) EDX (Map Sum Spectrum) graph and (d) EDS Layered Image of RP1A-1 nanocomposite. Figure S2a,b SEM images (c) EDX (Map Sum Spectrum) graph and (d) EDS Layered Image of RP2A-1 nanocomposite. Figure S3a,b present SEM images (c) EDX (Map Sum Spectrum) graph and (d) EDS Layered Image of RP3A-1 nanocomposite.

**Figure 2:**
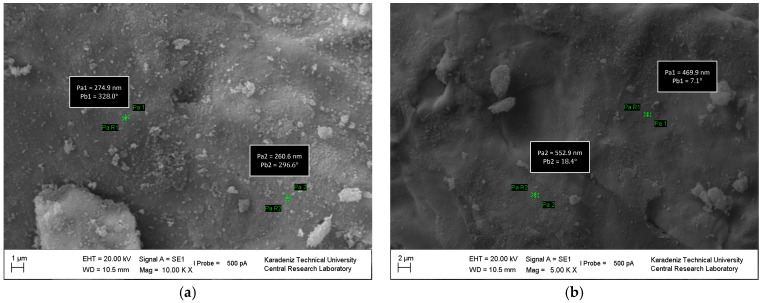

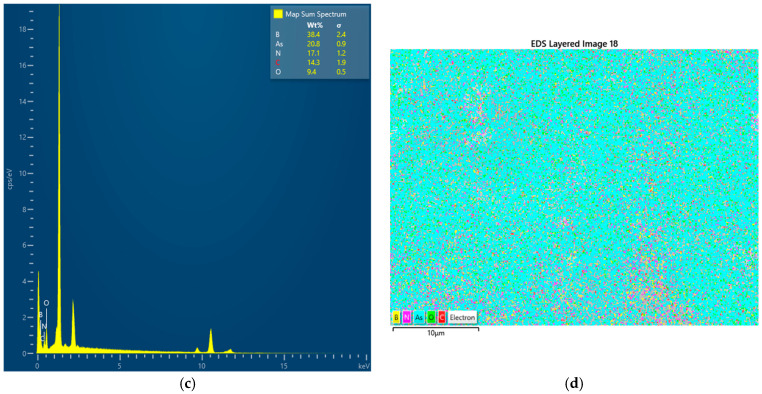
(**a**,**b**) SEM images, (**c**) EDX (Map Sum Spectrum) graph, and (**d**) EDS Layered Image of RP1ABN-1 nanocomposite.

SEM pictures of RP1ABN-1 nanocomposite are in Figure 2a,b, Map Sum Spectrum of B, N, O, C, and As atoms are in Figure 2c, and EDS Layered Image in Figure 2d. RP1ABN-1 nanocomposite is highly particulate, and average size values are between 260 and 276 nm and form some micrometric agglomerates. SEM micrographs (Figure 2a,b) reveal that As_2_O_3_ and BN NPs have cylindrical grain agglomerates ranging from 210 to 276 nm. On the other hand, it was observed that As_2_O_3_ and BN NPs had a homogeneous morphology in the composite. Microscopic image in Figure 2a, RP1A-2 shows the fine particle size and crystal structure of the nanocomposite. Figure S1a,b show SEM images (c) EDX (Map Sum Spectrum) graph and (d) EDS Layered Image of RP1A-1 nanocomposite. Figure S2a,b SEM images (c) EDX (Map Sum Spectrum) graph and (d) EDS Layered Image of RP2A-1 nanocomposite. Figure S3a,b present SEM images (c) EDX (Map Sum Spectrum) graph and (d) EDS Layered Image of RP3A-1 nanocomposite.

**Figure 3:**
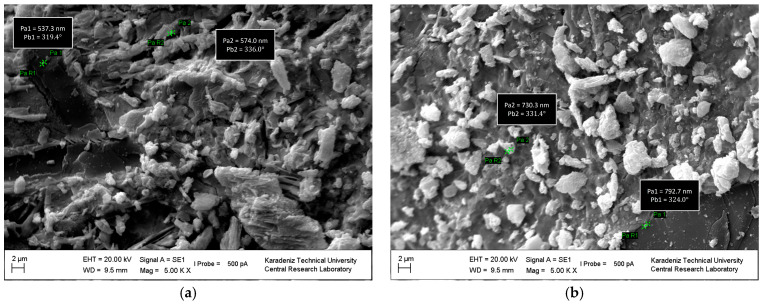

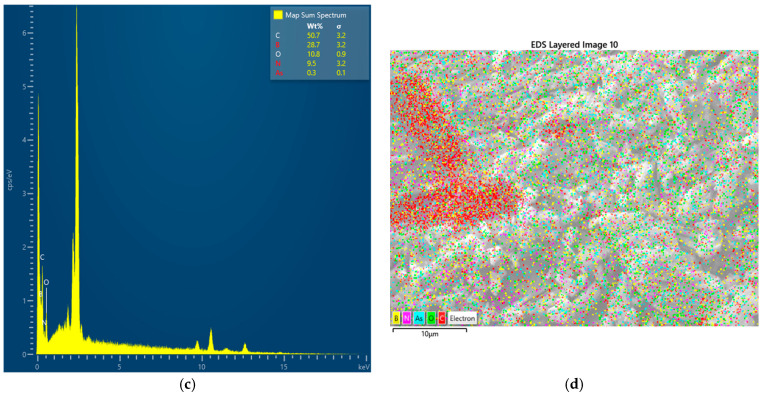
(**a**,**b**) SEM images (**c**) EDX (Map Sum Spectrum) graph, and (**d**) EDS Layered Image of RP2ABN-1 nanocomposite.

SEM photos of the RP2ABN-1 nanocomposite are shown in Figure 3a,b. As seen in these photographs, it exhibits a rod and granular structure. Rod size: width: 537.3 nm, in Figure 3a, and 730.3 nm in Figure 3b was measured. SEM (Figure 3a,b) images show that the RP2ABN-1 nanocomposite consists of 500–800 nm microspheres. The collection of these microspheres creates a network of interconnected hierarchical pores that are widely dispersed. SEM images of the RP2ABN-1 nanocomposite shown in Figure 3 show that the BN particles are rod-shaped and the As_2_O_3_ NPs are nearly spherical. The SEM images show that both particles in the nanocomposite are nanostructured, although there are agglomerations in some parts. At the same time, SEM images show that the morphology of the RP2ABN-1 nanocomposite is homogeneous, compact, rough, and cracked. As_2_O_3_ NPs accumulation in the form of agglomerates was observed on the surface of the composite (Figure 3a,b).

RP2ABN-1 nanocomposite EDX maps are shown in Figure 3c. It shows the distribution of carbon (C), oxygen (O), boron (B), nitrogen (N), and arsenic (As) elements in the nanocomposite and the presence of all C, As, B, N, and O elements in different colors. SEM and EDX images show smaller agglomeration sites of the NPs. Similar observations have been reported in previous studies [8,22,34,35].

**Figure 4:**
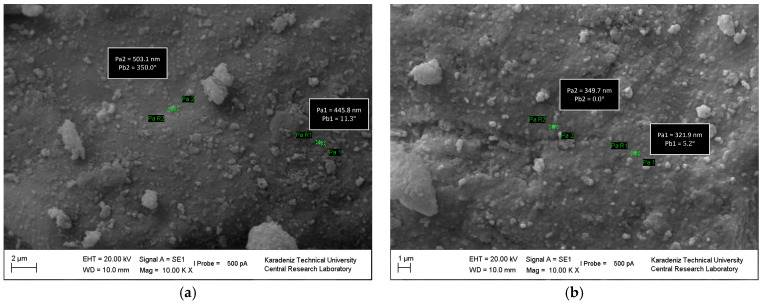

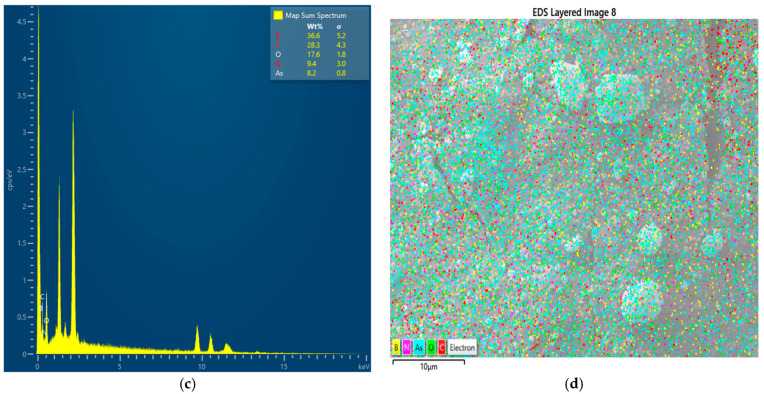
(**a**,**b**) SEM images (**c**) EDX (Map Sum Spectrum) graph and (**d**) EDS Layered Image of RP3ABN-1 nanocomposite.

SEM images of the RP3ABN-1 nanocomposite are shown in Figure 4a,b. The size of As_2_O_3_ and BN NPs on the PS-b-PEG (10,000) copolymer surface varies between 445 and 504 nm (Figure 4a) and 321 and 349 nm (Figure 4b). SEM EDX analysis values give map sum spectrums of B, N, As, O, and C atoms (Figure 4c). Figure 4d shows the EDS image. The EDS image of the RP3ABN-1 nanocomposite shows that the atoms in the content are homogeneously distributed.

The XRD diffraction patterns for examining the crystal structure of selected nanocomposites are shown in Figure S11, and their explanations are included in the Supplementary Materials.

### 3.3. Gamma Radiation Research Results of the Nanocomposites

#### 3.3.1. Investigation of Experimental MAC (*µ*_m_) and LAC (*µ*_L_) Values of the Nanocomposites

##### Investigation of Experimental MAC (*µ*_m_) Values of the Nanocomposites

**Figure 5:**
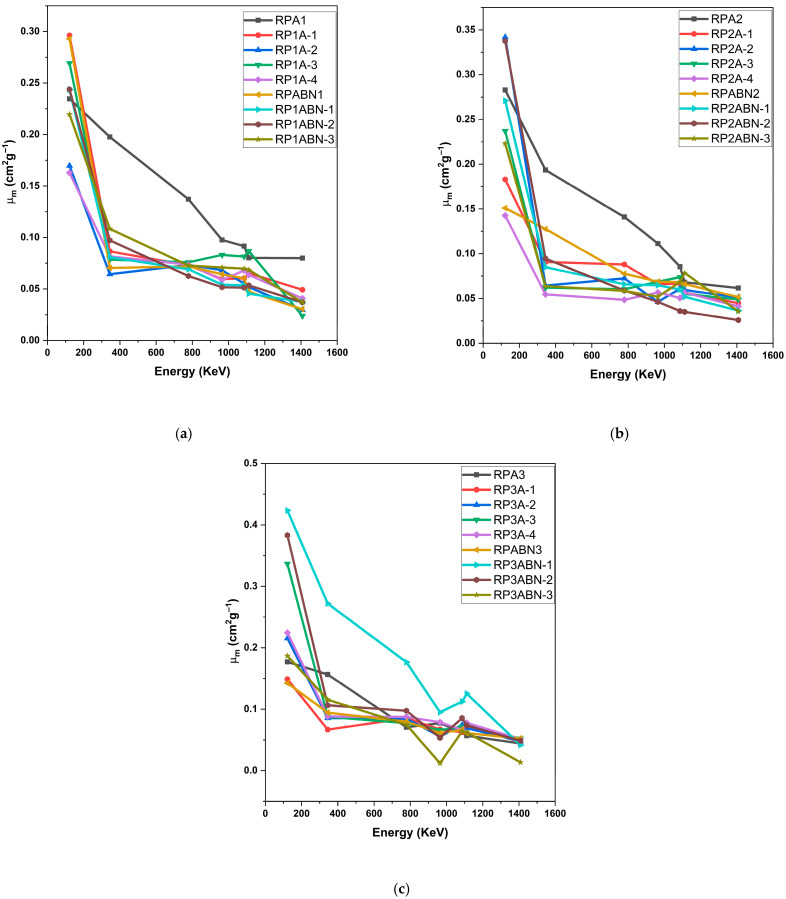
MAC (cm^2^/g) of As_2_O_3_- and BN-based PS-PEG copolymers: (**a**); RPA1, RP1A-1, RP1A-2, RP1A-3, RP1A-4, RPABN1, RP1ABN-1, RP1ABN-2, RP1ABN-3. (**b**); RPA2, RP2A-1, RP2A-2, RP2A-3, RP2A-4, RPABN2, RP2ABN-1, RP2ABN-2, RP2ABN-3. (**c**); RPA3, RP3A-1, RP3A-2, RP3A-3, RP3A-4, RPABN3, RP3ABN-1, RP3ABN-2, RP3ABN-3.

**Figure 6:**
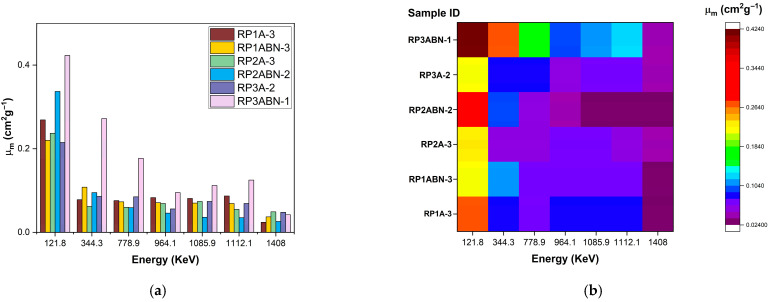
(**a**) Comparison of the most efficient nanocomposites according to their mass absorption coefficients. (**b**) Heat map showing the relationship between energy and mass absorption coefficients of the most efficient composites.

In Figure 5a–c the MACs of various As_2_O_3_ and BN NPs-based PS-PEG copolymers at different gamma-ray energies (from 121.78 keV to 1408.00 keV) are given. These graphs are labeled (a), (b), and (c), corresponding to three copolymers: RPA1, RPA2, and RPA3, and the MAC values of their nanocomposites. Experimental MAC results are given in Figure 5 and calculated using Equation (3). These values are based on the pellet properties given in Table 1. Table S2 compares the theoretical MAC values obtained from XCOM with the experimental MAC values. Figure 5a presents a comparison of the MAC protection properties of PS-b-PEG (1000) nanocomposites doped with As2O3 and BN NPs. The effect of sample thickness should be taken into account in radiation measurements. Thicker samples exhibit more pronounced MAC shielding properties. However, when evaluated with the pellet thicknesses given in Table 1, it is seen that RP1A-1 (0.296 cm^2^/g) and RP1A-3 (0.269 cm^2^/g) nanocomposites containing 50 wt% and 70 wt% As_2_O_3_ NPs provide the best MAC protection properties. The results indicate that among the nanocomposites prepared by adding As_2_O_3_ NPs to PS-b-PEG (1000), PS-b-PEG (1500), and PS-b-PEG (10,000) copolymers, the RP3A-3 nanocomposite, obtained with 70 wt% As_2_O_3_ NPs addition, had the highest MAC value (0.336 cm^2^/g). At 121.78 keV, RP3ABN-2 displayed the highest attenuation coefficient (0.423 cm^2^/g), followed by RP2A-2 (0.342 cm^2^/g) and RP3ABN-2 (0.383 cm^2^/g), and RP2ABN-2 (0.337 cm^2^/g). The MAC value of the RPA1 copolymer containing 50 wt% BN and PS-PEG (1000) copolymer increased from 0.235 cm^2^/g to 0.293 cm^2^/g in the RPABN1 nanocomposite. It can be concluded that the RP1ABN-1, RP1ABN-2, and RP1ABN-3 samples containing BN exhibited more stable shielding behavior and improved structural properties compared to the samples without this additive. Although the BN doping does not significantly increase, Figure 5b presents a comparison of the MAC shielding properties of PS-b-PEG Type (1500) doped nanocomposites. Similarly to Figure 6a, when Table S2 is examined, the effectiveness of RP2A-3 stands out among the PS-b-PEG Type (1500) doped nanocomposites.

Figure 5c includes the PS-b-PEG (10,000) doped nanocomposites. The As_2_O_3_ rich RP1A-3, RP2A-3, and RP3A-3 samples appear to be more effective in terms of radiation shielding. In order to obtain the most effective structural shielding properties of the samples, it is important to examine the LAC values free of this thickness factor and the HVL and TVL values obtained in parallel with this value. RPA1 and RPA2 also showed good performance (0.235 and 0.283 cm^2^/g, respectively), while RPA1BN1 showed a higher value (0.293 cm^2^/g) when 50% BN was added. This indicates that the addition of both BN and As_2_O_3_ significantly enhanced the attenuation for lower energies.

Considering the general trend with energy, RP1A-1: 0.296 cm^2^/g at 121.8 keV, decreasing to 0.049 cm^2^/g at 1408 keV, an approximately sixfold decrease was observed. RP1A-3: 0.269 cm^2^/g at 121.8 keV, decreasing to 0.024 cm^2^/g at 1408 keV, an approximately elevenfold decrease was observed. A comparison between the nanocomposites revealed that the highest initial value at 121.8 keV was 0.296 cm^2^/g for RP1A-1, and the lowest was 0.163 cm^2^/g for RP1A-4. Looking at the general trend, RP1A-1 and RP1A-3 have the strongest absorption capacity at low energy. RP1A-2 and RP1A-4 remain at relatively lower values. The energy dependence is clear and consistent, with strong shielding at low energy and weak shielding at high energy. The strongest nanocomposites are RP1A-1 and RP1A-3 at low energy. At high energy (1112 keV), RP1A-3 provides good shielding but drops rapidly at 1408 keV. In general, RPA1-containing nanocomposites can provide strong barriers against low-energy gamma radiation. However, their effectiveness decreases significantly at higher energies. Therefore, RPA1- and RPA1-containing nanocomposites may be particularly suitable for medical imaging, radiotherapy suites, and low- and medium-energy gamma shielding; however, they are not sufficient alone in environments such as high-energy nuclear power plants or deep space radiation. The µ values of the RPA2 and RP2A-containing nanocomposites RPA2A-1, RP2A-2, RP2A-3, and RP2A-4 decrease as the energy increases.

The RP2A-2 nanocomposite showed the highest and lowest energy performance at 121.8 keV. The lowest initial value belongs to the RP2A-4 nanocomposite at 121.8 keV. Results of low-energy studies of the nanocomposites containing RPA2 (PS-b-PEG (1500)) indicate that RP2A-2 is the nanocomposite that best absorbs low-energy gamma photons and is well-suited for medical radiology and nuclear medicine applications. In terms of medium-energy stability and RP2A-1, nanocomposites exhibit moderate tendencies and stable behavior. They also lose substantial performance at high-energy levels. Thus, they are not adequate for high-energy nuclear reactors or radiation exposure due to space alone. RPA2-based nanocomposites are a good nanocomposite family for low- and medium-energy gamma rays. The RP2A-2 nanocomposite is the strongest candidate at low energy levels (high absorption capacity of 0.342). The RP2A-3 nanocomposite is the most stable at high energy levels (with the least decrease of 0.074). In general, RPA2 nanocomposites can be used in low- and medium-energy medical, industrial, and environmental radiation protection applications. For RP3A-1, RP3A-2, RP3A-3, and RP3A-4 nanocomposites containing RPA3, the values of µ diminish upon increasing the energy. For RP3A-3 at 121.8 keV, it registered a reduction of 0.336 and 0.052 at 1408 keV, roughly a 6.5-time reduction. RPA3 (PS-b-PEG (1000)) registered a reduction of 0.177 and 0.044 at 1408 keV; roughly, RP3A-4 (0.225) comes in second at the same energy. RP3A-3 and RP3A-4 nanocomposites maintain the best protection at high energy levels. RP3A-1 is the weakest nanocomposite, exhibiting limited effectiveness at both low and high energy levels. From their overall performance, the nanocomposites containing RPA3 copolymer offer wider and well-rounded performance compared to nanocomposites containing RPA1 and RPA2. RP3A-3 and RP3A-4 nanocomposites maintain the best protection at high energy levels. RP3A-1 is the weakest nanocomposite, exhibiting limited effectiveness at both low and high energy levels. From their overall performance, the nanocomposites containing RPA3 copolymer offer wider and well-rounded performance compared to nanocomposites containing RPA1 and RPA2. Examining the range of 344.3–964.1 keV, the RP1ABN-3 nanocomposite has the highest shielding value at 344.3 keV. In the high-energy range (1085.9–1408 keV), the highest shielding value is again for the RP1ABN-3 nanocomposite. At 1408 keV, the shielding efficiency values for all nanocomposites are similar. The BN doping is particularly pronounced at low energy, particularly in the RPABN1 variant, with a value of 0.293, providing a significant increase compared to nanocomposites without BN doping. At medium energy, RP1ABN-3 appears to be more advantageous. At high energy, the effect of the BN doping decreases, and the values approach each other. The most prominent low-energy nanocomposite is RPABN1. The most prominent medium-energy nanocomposite is RP1ABN-3. It is apparent from Figure 5 that the MAC for all nanocomposites is high for 121.8 keV, and as the energy increases, the value of µ reduces sharply. RP2ABN-2 shows a decrease of about 13 times from a value of 0.337 for 121.8 keV to 0.026 for 1408 keV. RPABN2 shows a decrease of about 3 times from a value of 0.151 for 121.8 keV to 0.052 for 1408 keV. At the low energy of 121.8 keV, RP2ABN-2 has the highest shielding value. RP2ABN-1 and RP2ABN-3 follow, with RPABN2 having the lowest shielding value. In the high-energy range of 1085.9–1408 keV, the lowest values are again observed in RP2ABN-2. The next highest value is displayed by the RP2ABN-3 and RPABN2 nanocomposites. It is apparent that RP2ABN-3 and RPABN2 have higher stability at higher energy levels. The presence of BN makes a remarkable difference, especially at lower energy levels of 121.8 keV. At low energy, RP2ABN-2, with a MAC value of 0.337, is the strongest nanocomposite in the SI4 table and provides the highest protection against low-energy gamma radiation. This is particularly valuable for medical radiology, nuclear medicine rooms, and X-ray protective coatings. In terms of high-energy resistance, RPABN2 and RP2ABN-3 are the most durable nanocomposites. This indicates that they are relatively more stable against high-energy radiation. RPABN2 nanocomposites have a very strong absorption capacity for low-energy gamma rays. However, as energy increases, the absorption capacity of all nanocomposites significantly decreases. RP2ABN-2 is the strongest low-energy nanocomposite. RPABN2 and RP2ABN-3 are the most stable high-energy forms. RPABN2 nanocomposites are typically among the best at shielding against low-energy radiation, but as energy levels rise, their effectiveness declines. Experimental studies of RPABN3 nanocomposites revealed significant fluctuations of the µm across the estimated photon energy spectrum range (121.8–1408 keV). RP2ABN-2 is the strongest low-energy nanocomposite. RPABN2 and RP2ABN-3 are the most stable high-energy forms. The results present evidence of BN doping effects on gamma-ray attenuation, which becomes dramatically superior at the low-energy end. Comparative evaluation demonstrates just how important it is to compose optimally for some needs: the low-energy shielding of RP3ABN-1 is better, and the high-energy shielding of RP3ABN-2 is better. The RP3ABN-3, however, is the worst of the rivals given its higher instability over the range that was under consideration. The overall performance of RP3ABN-1 and RP3ABN-2 together gives the best shielding across the whole range of photon energies. These two materials complement each other, making them the most suitable candidates for applications concerning the protection of radiation that require interaction with different energy levels, especially when there is a need for low- and high-energy gamma protection.

High-energy photons (X-rays and γ-rays) interact with matter and are ultimately absorbed through energy transfer. This process is related to the atomic composition of the target materials and has nothing to do with their morphology and structure. The three main mechanisms of photon–matter interaction include the photoelectric effect, Compton scattering, and pair production. Each of these three interactions occurs with a certain relative probability. In the photoelectric effect, the incident photon interacts with a nuclear electron in the target atom, causing the nuclear electron to be ejected. Some of the energy is used to overcome the electron’s binding energy, and the remainder is converted into the kinetic energy of the ejected photoelectron. The photoelectron ejected leaves the atom in an unstable excited state. The photoelectric effect occurs when an incident photon interacts with a nuclear electron in a target atom, causing the nuclear electron to be ejected. Some of the energy is used to overcome the electron’s binding energy, while the rest is converted into the kinetic energy of the ejected photoelectron. Unlike the photoelectric effect, Compton scattering involves an inelastic scattering of the incident photon. Instead of being lost, it transfers some of its energy to the electron, which is then scattered with diminished energy during Compton scattering. The orbital electron absorbs a portion of the photon’s energy (greater than its binding energy) and is ejected from the atom as a recoil electron [36]. The Compton effect typically occurs in the outer electrons of a nucleus. The scattered photons, however, possess a significant amount of energy and are ultimately absorbed through the photoelectric effect or multiple scattering. The MACs values decrease along with a swell in gamma radiation energies. The occasion for this situation is the interaction of gamma radiation with materials via a photoelectric effect, Compton scattering, and pair production. The photoelectric effect is most influential in the low gamma energy regions; for this case, MACs values are greater in these gamma radiation energy areas. Compton effect is overpowering at middle radiation energy areas, conversely, pair production is dominant at elevated gamma irradiation energy regions; herewith, MACs worthies start to reduce with the escalation of gamma energies [8,34,35].

Even more than RPA2 itself, RP2A-2 and RP2ABN-2 show peak µ_m_ at low energies (121.78 keV). This implies better attenuation, maybe from a rise in doped As_2_O_3_ or BN. This indicates that RPA3 base composition, combined with BN and As_2_O_3_, yields superior shielding performance across a broader energy range. The efficacy of gamma-ray shielding materials is significantly influenced by the atomic number (Z) of their components. Arsenic (Z = 33) and boron (Z = 5) have distinct attenuation properties. The uniform trend observed in all graphs indicates increased As_2_O_3_ NP concentration that presumably enhances attenuation, particularly at lower energy levels. BN contributes at moderate to elevated energy, where Compton scattering prevails. Table 3 shows where the best performance is noteworthy.

Figure 6a,b show the relationship between the $\mu_{m}$ values and the heat map displaying the comparison of the best MAC values among all composites and nanocomposites, respectively. The RP3ABN-1 (0.272 cm^2^/g), RP2ABN-2 (0.095 cm^2^/g), and RP3ABN-3 (0.102 cm^2^/g) composites maintain their efficiencies. The RP3ABN-1 nanocomposite is superior at all energies except 1408 keV. At 1408 keV, the BN-doped samples are efficient. The BN-doped composites maintain their absorption efficiency while increasing the strength of the composites.

Figure 6b shows the heat maps of these nanocomposites against energy. Figure 6b shows the heat maps of these nanocomposites against energy. At a low energy of 121.8 KeV, the MAC values for radiation protection effectiveness of the nanocomposites are higher than at other energy values. Within this natural behavior, the heat map shows the most prominent example for each energy. RP3ABN-1 has the highest MAC values at 121.8, 344.3, 778.9, 1085.9, and 1112.1 KeV, while RP2A-3 and RP3A-2 have the highest MAC values at 1408 KeV.

Sharma and colleagues investigated the gamma-ray blocking properties of polymer concretes containing 5 wt% Bismuth (III) Oxychloride (BiClO). At 59.5 keV, the MAC was about 0.379 cm^2^/g, closely matching FLUKA and XCOM calculations. At 1112.1 keV, the MAC was around 0.061 cm^2^/g, also consistent with simulation data [37]. El-Mesady et al. prepared PVC polymers with basalt NPs (0–50 wt%). Increasing basalt content raised the linear attenuation coefficient from 14.42 to 23.86 cm^−1^ at 0.015 MeV and from 0.030 to 0.048 cm^−1^ at 15 MeV. The MAC improved with basalt concentration, and the HVL thickness decreased from 0.048 to 0.029 cm at 0.015 MeV [38]. Ahmed M. El-Khatib analyzed SnO_2_, Bi_2_O_3_, and CdO-doped silicone rubber nanocomposites. The value of MAC measured after the experiments for 778.9 keV was between 0.0738 and 0.0798 cm^2^/g, indicating efficient gamma attenuation [39]. Our PS-PEG block copolymers and As_2_O_3_, and BN nanoparticles, had efficient gamma radiation-shielding properties. Values obtained for MAC were at a comparable energy range of 0.059 to 0.177 cm^2^/g for these sets of materials, as seen in Figure 6a, where the materials had better shielding properties. Our developed nanocomposites had higher MAC values than the conventional standard reference systems.

In the literature, the MAC values of the 50% PbO/high-density polyethylene (HDP) system were determined as 0.1182 cm^2^/g at 0.662 MeV and 0.064 cm^2^/g at 1.173 MeV. RP3ABN-1 (0.177 cm^2^/g at 778.9 keV) and RP1ABN-3 (0.037 cm^2^/g at 1408.0 keV) nanocomposites developed in this study gave MAC results close to the PbO/HDP reference values [40], and the theoretical data in Table S2 support these findings. In comparative analyses, when compared with literature studies such as BiClO-added polymer concretes (0.379 cm^2^/g, 59.5 keV), PVC polymers with basalt NPs (LAC: 23.86 cm^−1^, 0.015 MeV), and SnO_2_/Bi_2_O_3_/CdO-added silicone rubber systems (MAC: 0.0738–0.0798 cm^2^/g, 778.9 keV), the present nanocomposites showed similar MAC results. They also observed that these materials possess a competitive level of performance compared to that of the values reported by Z. Alsayed et al. [41] for lead-doped materials. These materials’ properties establish that these nanocomposites can be environmentally friendly alternatives to lead materials. Table 4 shows comparison of the nanocomposites and conventional shielding materials. “PS-b-PEG” matrices consist of permanently cross-linked networks where PEG blocks (Mn = 1000, 1500, and 10,000 g/mol) act as multifunctional cross-links between polystyrene segments. Hence, differences in size of the PEG blocks and composition ratio PS/PEG simultaneously influence the cross-link density and network architecture, directly affecting high-Z filler confinement and consequent radiation attenuation coefficient values for high-Z NPs As_2_O_3_ and BN NPs. When loaded for the three respective copolymers RPA1 (PEG1000), RPA2 (PEG1500), and RPA3 (PEG10,000) with high-Z inorganic NPs, MAC values for composites filled with As_2_O_3_: RP1A-1/RP1A-3 (in RPA1 matrix), RP2A-2 (in RPA2 matrix), and especially for RP3A-3 (in RPA3 matrix containing 70% As_2_O_3_) at 121.78 keV energy increased successively to 0.336 cm^2^ g^−1^, being higher than MAC for any composition containing just As_2_O_3_ NPs for RPA3 matrix than for any others. Likewise, for hybrid composites containing As_2_O_3_/BN NPs, RPA3ABN-2 and RP3ABN-1 made from PS-b-PEG (10,000) showed higher attenuation coefficients at 121.78 keV up to 0.423 cm^2^ g^−1^. This implies higher excess amount of NPs for which larger PEG blocks and smaller effective crosslinking densities of RPA3 matrix result in a “loosely” cross-linked network for higher filler loadability and homogenous filler distributions without apparent aggregation to larger filler-rich masses while being unfavorable for larger filler loadability to avoid thick macromolecular heterogeneous masses for RPA1 (PEG1000) having higher effective crosslinking densities to result directly in MAC values indicative of moderate to high-Z at 50–70% As_2_O_2_ filler contents with resultant higher homogenous MAC values. HVL values for radiation attenuation coefficients for optimized bulk composites: 0.85 cm for RP3A-3 and 0.97 cm for RP2ABN-2 at 121.78 keV energy give values similar to those for Pb and W upon adjusting for bulk densities to afford direct insights into highly effective influence of network architectures on respective bulk attenuation pathways at desired higher filler loadability for effective radiation protection applications at desired radiation safety to each human being similarly to each surrounding Pb/W. In brief, filler acceptance/dispersion can be increased by having a low crosslinking density combined with high PEG values/chain length (RPA3), making higher values of effective ‘Z’ and ‘density’ at any ‘thickness’ possible, while dense resins (RPA1) possess low filler acceptance combined with slight reductions in attenuation performance at similar concentrations.

When analyzed for microstructural and interfacial interactions responsible for the improvement of protection efficiency, As_2_O_3_ NPs are found to be homogeneously distributed inside the pore-like PS-PEG matrix. TEM analysis shows that As_2_O_3_ NPs are homogeneously dispersed inside the PS-PEG matrix and no large clusters of inorganic components are present inside the continuous copolymer matrix.

This composition provides the maximum number of interfaces between the polymer and filler, as well as ensuring that photons experience a homogeneous high-Z material surrounding. BN induced densification and interfacial formation. SEM-EDX analysis of BN-containing samples like RP3ABN-1 shows homogeneous distributions of B, N, As, O, and C components within the PS-b-PEG (10,000) matrix and few BN platelets. According to this paper, introduction of BN NPs enhances interfacial bonding between filler and matrix, minimizes voids and porosity, as well as effective stack thickness and photon density, while active edge planes of BN come into intimate contact with PS-PEG chains surrounding them to give rise to ‘coherent interfaces’ associated with ‘interface shielding.’ This is because many sub-nm-scale interfacial areas between As_2_O_3_ and BN NPs within PS-PEG act as photon and secondary electron scattering centers to prolong their paths to increase chances of energy loss or photon absorption while being confined within the matrix. This is behind MAC/LAC and HVL/TVL enhancements for BN-containing hybrids against BN-free hybrids of equivalent matrix composition.

**Figure 7:**
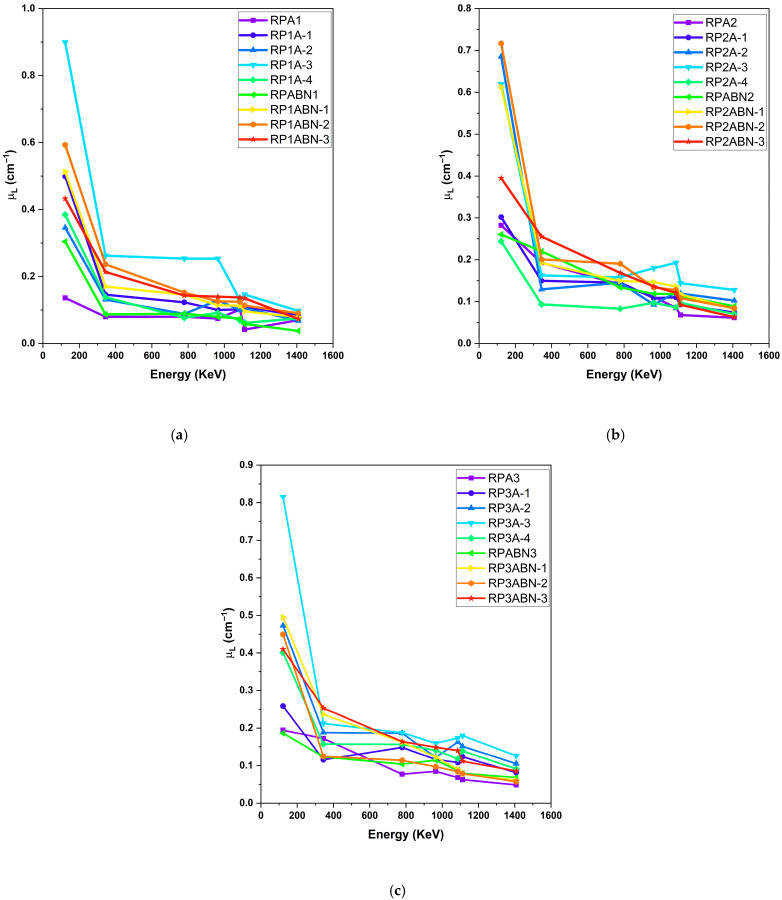
LAC ($\mu_{L}\;({cm}^{-1})$) of As_2_O_3_- and BN-based PS-PEG copolymers: (**a**); RPA1, RP1A-1, RP1A-2, RP1A-3, RP1A-4, RPABN1, RP1ABN-1, RP1ABN-2, RP1ABN-3. (**b**); RPA2, RP2A-1, RP2A-2, RP2A-3, RP2A-4, RPABN2, RP2ABN-1, RP2ABN-2, RP2ABN-3. (**c**); RPA3, RP3A-1, RP3A-2, RP3A-3, RP3A-4, RPABN3, RP3ABN-1, RP3ABN-2, RP3ABN-3.

MAC shows the absorption capacity depending on the composition and density of the material. LAC is closer to the original value of the material component. It provides a shielding efficiency free from possible deviations during the experiment. Figure 5 and Figure 7 show the energy-dependent distributions of the MAC and LAC experimental values of the nanocomposites. The obtained results show that the samples with high LAC values are more effective in absorption than the samples with low values. Experimental and theoretical LAC values (XCOM data) are given in Table S3. When the data are compared, it can be seen that the experimental study follows a flow in accordance with the theoretical results. Although it is predicted that the MAC and LAC activities will decrease as the energy values increase, it is also observed with the XCOM data. This decrease is also reflected in the experimental results. This character of the experimental values shows that the experiment was carried out correctly.

- The Statistical Analysis of Multiple Samples

Radiation measurements were performed by taking three consecutive separate measurements for each sample; the samples were measured simultaneously on the same day, with the detector geometry partially moved. The average of these measurements was compared with the blank measurement to calculate MAC and LAC values. For the RP3A-1 sample, the graphs in Figures S7–S10. clearly show the 778.90, 964.08, 1085.87, 1112.07, and 1408.01 keV peaks, while the 121.78 and 344.28 keV peaks are less pronounced; peak areas were analyzed separately with the Origin program (Figures S7–S10).

Table S6 compares the average with the RP3A-1 measurements. Despite the stability of the ^152^Eu source, small deviations were observed due to detector sensitivity. Taking at least three measurements for each sample increased the reliability of the average values, confirming that the deviations were statistically stable. This approach ensured experimental consistency by minimizing potential outliers. The apparent fluctuations in the MAC values of RP3ABN-1 (0.423 cm^2^/g at 121.78 keV), RP1A-3 (0.269 cm^2^/g), and RP2A-2 (0.342 cm^2^/g) are due to the pellet thicknesses, while the HVL values (0.91, 0.77, 1.01 cm, respectively) give more consistent results. Multiple measurements and statistical analysis confirm the experimental origin of the differences, highlighting the reliability of the radiation-shielding performance. In this study, the standard deviation for the experimentally obtained LAC values was calculated and is given in Table S3. Since these LAC values are the basis of all other radiation data, the standard deviations are at the same level.

##### Investigation of Experimental LAC (μ_L_) Values of the Nanocomposites

Figure 7 shows the *μ*_L_ values of nanocomposites composed of various As_2_O_3_ NPs, BN NPs, and PS-PEG copolymers at different gamma-ray energies ranging from 121.78 keV to 1408.01 keV. In Figure 7a, RP1ABN-2 nanocomposite obtained from BN-doped samples presents a more effective performance at lower energies (121.78 keV, 344.28 keV, and 778.9 keV). At higher energies (964.08, 1085.87, and 1112.07 keV), RP1ABN-3 is more functional in terms of radiation-shielding activity. An exception for RP1ABN-2 is 1408.01 keV. This formation once again shows that BN deposition is effective in shielding. RP2A-3 stands out among all samples in Figure 7b, while RP2ABN-2 and RP2ABN-3 nanocomposites also stand out with their activities in BN-doped nanocomposites. Similarly, in Figure 7c, RP3A-3 is the most effective radiation shield. RP3ABN-3 appears to be more effective in BN-doped nanocomposites. All these results show that RP1A-3, RP2A-3, and RP3A-3 nanocomposites absorb strong radiation. The LAC value decreases continuously with increasing energy. RP1A-3 and RP1ABN-1 possess maximum energy of the gamma rays at lower energies, especially at 121.78 and 344.28 keV. The RPA1 copolymer exhibits lower LAC values throughout the spectrum, which are associated with lower efficacy of protection.

The experimental LAC values of the RPA1 copolymer and its doped nanocomposites (RP1A-1, RP1A-2, RP1A-3, RP1A-4) show high values at low photon energies (121.8 keV) and a decrease as the energy approaches 1408 keV (Table S3 and Figure 7a). RP1A-3 also maintained its outstanding performance at higher energies (0.272 at 1085.9 keV) and therefore has the highest shielding material properties in this series. In general, the outcomes revealed that the highest attenuation of low-energy gamma rays is offered by RP2A-2. Conversely, RP2A-3 is identified to be the most steady and well-rounded among all materials tested, across the whole energy range, and possessing the highest stability for high energy. The experimental LAC values for the RPA3 copolymer and the RP3A-1, RP3A-2, RP3A-3, and RP3A-4 nanocomposites decrease as photon energy increases (Table S3 and Figure 7c). High µ_L_ values are observed for all samples at 121.8 keV, while the values decrease steadily as the energy increases to 1408 keV. RP3A-3 has the highest attenuation ability for all energy ranges (e.g., 0.815 at 121.8 keV and 0.126 at 1408 keV). RP3A-2 exhibits the second highest strength amongst the nanocomposites, with elevated values in the low- to medium-range energy and stable values at higher energy (0.473 at 121.8 keV and 0.105 at 1408 keV). While RP3A-1 and RP3A-4 offered intermediate performance, the RPA3 copolymer sample gave the lowest LAC values at all energy steps (0.195 at 121.8 keV to 0.049 at 1408 keV).

In the medium-energy range (344.3–964.1 keV), RP3A-3 and RP3A-2 have high values, with RP3A-1 remaining at a moderate level, while RPA3 and RP3A-4 exhibit relatively lower values. RP3A-3 exhibits a maximum absorption at higher energies (1085.9 keV and above), followed by RP3A-2. Although the attenuation of RP3A-4 and RP3A-1 is moderate, the lowest attenuation is observed for the RPA3 copolymer. The RP3A-3 nanocomposite doped with RPA3 greatly increases the attenuation of low- to medium-energy photons. The LAC values of RPABN1, RP1ABN-1, RP1ABN-2, and RP1ABN-3 nanocomposites decrease systematically with the increase in photon energy.

The LAC values for RPABN1 and the RP1ABN-1, RP1ABN-2, and RP1ABN-3 nanocomposites show a systematic decrease with increasing photon energy. RP1ABN-1 initially exhibits a higher LAC value at low energy (0.513), decreasing to intermediate levels at higher energy. In contrast, RPABN1 exhibited the lowest LAC values in all regions (e.g., 121.8 keV: 0.362; 1408 keV: 0.037). The unprecedented superiority of RP1ABN-2 at low energy (121.8 keV) underscores the key role of compositional/doping design for effective low-energy gamma photon attenuation. In the intermediate energy range (344.3–964.1 keV), RP1ABN-2 again takes the lead, with RP1ABN-3 following closely behind; these two nanocomposites possess significant advantages over their copolymers. At higher energy (1085.9 keV), the difference between the nanocomposites decreases slightly, with RP1ABN-2 still maintaining the highest residual attenuation, followed by RP1ABN-3, and RPABN1 remaining the weakest performer. The BN doping provides a significant increase in the mid-energy range, particularly for the RP1ABN-2 nanocomposite. RP1ABN-3 ranks second in the mid- and higher energy ranges. Consequently, RP1ABN-2 is the best of the RPABN1 nanocomposite family. Its higher low-energy starting value and higher energy shielding capacity make it the ideal choice for any application requiring effective radiation shielding in the mid-energy spectrum. RP2ABN-1 (and partially RP2ABN-3) is preferred because it can accommodate a very wide range of energy levels. The results confirm that BN NPs greatly improve the shielding efficiency for the RPABN2, RP2ABN-1, RP2ABN-2, and RP2ABN-3 nanocomposites (Figure 7). The corresponding point where the maximum attenuation is observed is 121.78 keV. This is because of the presence of the K-edge absorption of arsenic (As, Z = 33), contributing to the increased photoelectric absorption. After this value, the attenuation is substantially reduced, and because of the interaction of photons, the value is close to zero for higher energy. The addition of BN NPs to the As_2_O_3_-PS-PEG copolymers makes the presence of this compound beneficial for its radiation-shielding properties. The nanocomposites made from RP1A copolymer, especially RP1A-3 and RP1ABn-1, can be considered optimal for the protection of low-energy gamma-rays.

In Figure 7c, at 121.78 keV, the RP3A-3 and RP3ABN-1 nanocomposites have higher attenuation efficiencies. At energies above 121.78 keV, the RP3ABN-3 nanocomposite stands out. At 344.28 keV, it has the best absorption efficiency among the BN-doped samples and all samples. The BN-doped As_2_O_3_ PS-PEG nanocomposites (RP3ABN-1, 2, 3) outperform the copolymer and individually doped nanocomposites. The hybrid systems provide improved attenuation. The LAC values start to decrease and evolve towards almost similar results among the samples at 1085.87 keV. However, RP3A-3 and RP3ABN-3 still have a clear advantage. Experimental and theoretical LAC values (XCOM data) are given in Table S3. When the data are compared, it can be seen that the experimental study follows a flow in accordance with the theoretical results.

**Figure 8:**
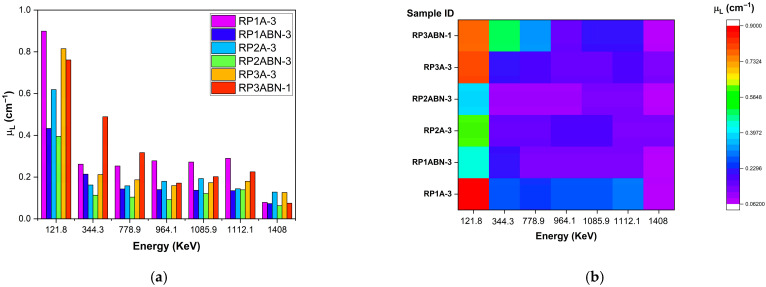
(**a**) Comparison of µ_L_ for the block copolymers and the nanocomposites. (**b**) Heat map.

Figure 8a,b present the LAC values and heat map for the block copolymers and the nanocomposites. In Figure 8b, the linear mass absorption values of these nanocomposites are evaluated on the heat map. RP1A-3 gave the highest values at 121.8, 1085.9, 964.1, and 1112.1 KeV, while the RP3ABN-1 nanocomposite showed high activity at 334.3 and 778.9 KeV. At 1408 KeV, RP2A-3 and RP3A-3 gave high linear absorption values. Furthermore, the heat map also shows that the linear absorption values obtained from the nanocomposites at higher energies are quite close to each other. In this context, parallel to the MAC values, RP3ABN-1 stands out as the sample with the best radiation-shielding effectiveness. These results demonstrate the value of BN doping.

The addition of BN NPs improves filler–matrix adhesion, reduces voids, and increases the effective bulk thickness for photon attenuation, increasing the overall composite density [42]. In a study by Sato et al., the radiation-shielding effect of the resulting composite films was attributed to interfacial affinity because the h-BN filler had a larger specific surface area [43]. In this study, the active edge planes of the BN nanoparticle filler chemically interacted with the surrounding PS-b-PEG matrix. The enhanced interactions resulted in consistent interfaces and an interfacial shielding effect. Adding BN NPs reduces porosity and voids (fewer low-density paths), increases the effective bulk thickness, and creates many nanometer-scale interfaces that increase scattering and path length for photons and secondary electrons (increasing the probability of energy loss before escape). As a result, well-dispersed BN NPs within the PS-b-PEG polymer matrix form numerous interfaces. These interfaces act as scattering centers for secondary electrons and photons, increasing their effective path length and the probability of absorption within the material. It is worth mentioning that the BN particles added to the composite suffer from the lack of interfacial adhesion to the polymer matrix, resulting in limited enhancement or even decay of mechanical strength. In order to improve the interfacial adhesion between BN particles and the polymer matrix, Shin et al. [44] carried out a surface modification of BN particles and developed HDPE/m-BN composites in which m-BN particles can achieve homogeneous dispersion. The adhesion between BN particles and polymer matrix was greatly improved due to the affinity between the C-C bonds in HDPE and the C-C bonds in the silane moieties. Compared to HDPE/BN composites (without surface modification of BN particles), the prepared HDPE/m-BN composites exhibited more homogenous filler dispersion, higher tensile modulus and superior neutron shielding performance. Shang et al. [45] developed a neutron shielding composite film with multilayer structure by alternating high density polyethylene/hexagonal boron nitride (HDPE/hBN) layers and low-density polyethylene (LDPE) layers. Multilayer PE/hBN films studied by Shang et al. exhibited better neutron shielding efficiency than random PE/hBN films due to the controllable periodic distribution of hBN particles, increasing the probability of collision between incident photons and flake-shaped particles.

Within the entire photon energy range examined (121.78 to 1408.0 keV), it is seen from Figure 5 for MAC and also confirmed by HVL/TVL plots for LAC values that PS-PEG/As_2_O_3_/BN nanocomposites display MAC and LAC attenuation similar to high-Z polymer composites—namely, the mass attenuation coefficient decrease.

At the low-energy side (121.78 keV), all composites have their highest MAC and LAC values. The hybrid material RP3ABN-1 has the highest MAC value of 0.383–0.423 cm^2^ g^−1^ for different pellet thicknesses and also the highest LAC value of 0.761 cm^2^ g^−1^ among BN-doped composites, which clearly shows higher attenuation. Other composites with high LAC values of 0.815–0.899 cm^−1^ are RP1A-3. Between 344 and 964 keV, MAC and LAC both drop steadily by a factor of 3 to 6 for all compositions.

In the intermediate-energy range (344–964 keV), although there is a reduction overall, there are As_2_O_3_-containing composites (RP1A-3, RP2A-3, RP3A-3) and BN-containing systems (RP2ABN-2, RP3ABN-1).

On the high-energy side (1085–1408 keV), attenuation coefficients approach their lowest values for higher photon energies. For instance, MAC values reduce from 0.30 to 0.42 to 0.02–0.05 cm^2^ g^−1^ for 121.8 to 1408 keV photon energy ranges. However, for 1408 keV photons too, RP3ABN-1, RP3A-3, and RP2A-3 approach comparatively better attenuation coefficients than others. This is because of changes in principal interaction types for higher photon energy values. In the low-energy region (121–344 keV), the photoelectric effect is dominant, leading to substantial attenuation and a sharp decay of MAC values. The high-Z As (A = 33) contribute substantially to attenuation coefficients or absorption, thereby providing higher values of MAC (0.30–0.42 cm^2^ g^−1^). The BN compound contributes little to the photoelectric effect because of its low atomic numbers, B and N.

In the medium-energy range (778–1085 keV), Compton scattering takes dominance. MAC plots become flattened, LAC becomes gradually slower, suggesting a predominance of electron density contribution over high-Z material absorption. These plots remain independent of filler types and have similar slopes for all composites. In the high-energy region (≥1 MeV), Compton scattering continues to be dominant; pair production continues to remain negligible. In the 1085–1408 keV energy range, the slight MAC and LAC decay continues to result mainly from Compton scattering. Pair production is not significant for these polymer composites with As/Z = 33 because for pair production to occur higher Z values and higher energy photons than those considered here are required, and also no rise or plateau region signifying pair production is noticed from experimental data.

Hence, for the full range of energy measured for attenuation, the factors controlling attenuation are the photoelectric effect (121–344 keV), Compton scattering (778–1408 keV), and no pair production for these As (atomic number (Z) = 33)-containing polymer composites.

To begin with, quantitative analysis of MAC data clearly appears to indicate BN-containing samples to possess better performance than BN-free samples for a photon energy range of 121 to 778 keV. This is because, for instance, MAC for PS-PEG (10,000) matrix at 121.78 keV is 0.336 cm^2^ g^−1^ for optimum As_2_O_3_-only formulation RP3A-3 and 0.423 cm^2^ g^−1^ for RP3ABN-1-containing BN. Similarly, for 50 wt% BN-doped samples RPA1 to RPABN1, MAC values improve from 0.235 to 0.293 cm^2^ g^−1^. Additionally, at 344.3 and 778.9 keV photon energies, MAC values for RP3ABN-1 are found to be higher than those for its As_2_O_3_-only samples. This is noteworthy considering improvement at 1408 keV is also very significant for BN-doped samples.

Second is the explanation of the energy dependence of attenuation to underline BN’s role. According to explanations presented in the paper, at low photon energies dominated by the photoelectric effect principle for attenuation, As_2_O_3_ (Z = 33) is dominant. BN (Z^−^ = 5) has a stronger effect at medium to high energies, where Compton scattering is the main process. Both mechanisms are significant in the 121–778 keV range. BN platelets and NPs create many more interfaces with the PS-PEG matrix. The TEM/SEM observations and published data have confirmed that these interfaces improve filler/matrix adhesion, filled voids, and low-density channels, and also increase the optical path length for both photons and secondary electrons. This means that photons not trapped in high-Z As_2_O_3_ photoelectric events experience Compton scatterings within BN-rich areas and interfaces before being emitted from the sample. This raises the probability of photon energy loss or absorption.

In our study, the PS-PEG/As_2_O_3_/BN nanocomposites do not match Pb or W in absolute attenuation per unit thickness, but they come close enough in shielding performance that they become quite competitive for practical use when considering density and biocompatibility. In comparison with Pb and W (attenuation vs. density and biocompatibility), RP3ABN-1, the best-performing hybrid at 121.8 keV, achieves a MAC value of 0.383–0.423 cm^2^ g^−1^ and a LAC value of 0.761 cm^−1^; RP1A-3 and RP3A-3 also show high LAC values of 0.899 and 0.815 cm^−1^, respectively. These values are close to those for Pb (μ = 1.24 cm^−1^) and W (μ = 1.10 cm^−1^) at the same energy.

The corresponding HVL values of the best nanocomposites are 0.77–0.97 cm, compared to 0.56 cm for Pb and 0.63 cm for W at 121.8 keV. Therefore, our materials require approximately 1.3–1.7 times more thickness to achieve the same 50% attenuation, but their densities are only 1.9 g cm^−3^, compared to 11.34 g cm^−3^ (Pb) and 19.25 g cm^−3^ (W). As a result, the areal mass (ρ × HVL) of the nanocomposites for an equivalent attenuation is several orders of magnitude lower than that of Pb/W, providing a significant weight advantage for mobile or wearable shields. Compositionally, PS-PEG and BN are more biocompatible and environmentally friendly than Pb or W. As_2_O_3_ is toxic in its free form, but in our design it is anchored within a cross-linked PS-PEG matrix and co-dispersed with BN, providing a high-Z contribution to attenuation while minimizing direct contact and potential leakage. Therefore, the overall system is a lead-free, lighter, and more flexible alternative to traditional metal shields. When examined in terms of weight-per-unit efficiency and HVL in practical applications for medical imaging barriers and wearable gowns, the relevant factor is often the areal density at the target transmission, rather than the thickness alone. Because our composites have a density approximately 6–10 times lower than Pb/W, their mass per unit area remains much smaller, even with a thicker HVL. This means that for a given level of protection in the diagnostic range of 120–400 keV, a PS-PEG/As_2_O_3_/BN panel or garment can be several times lighter than an equivalent Pb or W shield. In practice, this translates to shields that are easier to wear for extended periods (reduced musculoskeletal load for personnel and patients) and easier to integrate into flexible or shaped components (curtains, mobile displays, or conformal inserts), while also providing clinically significant attenuation (HVL in the sub-centimeter to 1 cm range at diagnostic energies).

#### 3.3.2. The Calculating of HVL, TVL, MFP, and RPE (%) Values of the Nanocomposites

##### The Calculation of Half-Value Layer (HVL) Values of the Nanocomposites

**Figure 9:**
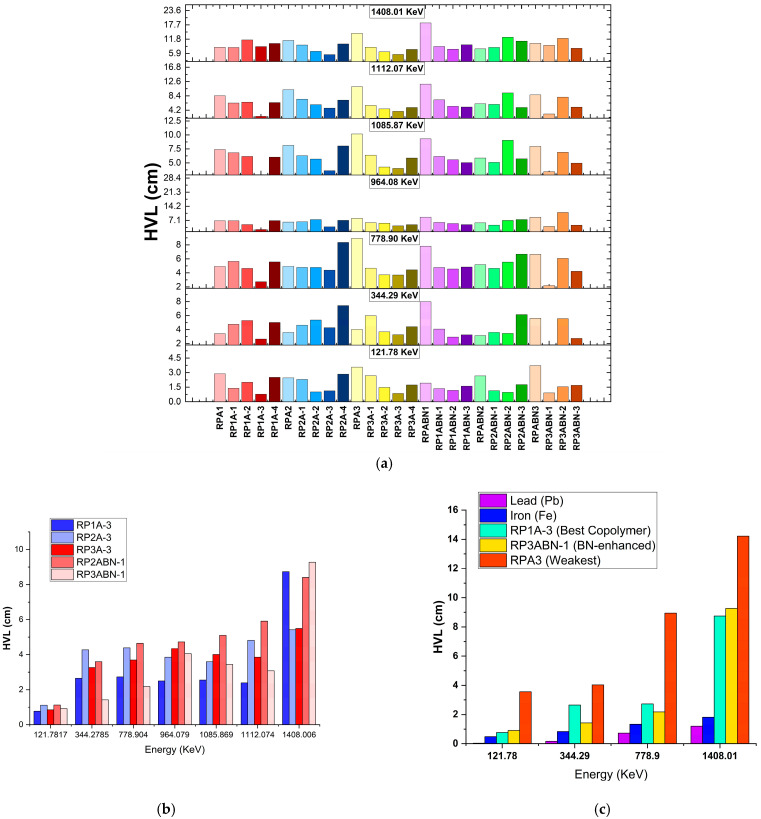
(**a**) HVL (cm) of As_2_O_3_ and BN-based PS-PEG copolymers. (**b**) Comparison of samples with the best HVL value. (**c**) Comparison of nanocomposites with natural radiation shields known in nature via HVL.

Figure 9a and Table S4 show the HVL values of the nanocomposites. A higher attenuation coefficient (μ) and correspondingly lower HVL values indicate a higher photon absorption ability of the material. Therefore, a lower HVL is an indication of better radiation-shielding performance.

Among the studied nanocomposites, the lowest HVL value was found for RP1A-3 (0.77 cm^3^) with 121.78 keV. The lowest value among the BN-doped nanocomposites was obtained for the RP3ABN-1 nanocomposite. While the HVL values for samples without BN doping varied between 0.77 and 3.56 cm^3^, they varied between 0.91 and 3.72 cm^3^ for BN-doped samples. Consequently, the absorption efficiencies of the nanocomposites increase with the addition of BN. RP3ABN-1 is the most effective nanocomposite at 344.28 and 778.90 keV. RP1A-3 has better shielding properties at 964.08, 1085.87, and 1112.07 keV, and the best HVL value at 1408 keV belongs to RP2A-3. In BN-doped samples, the best value at this energy level belongs to RP1ABN-2. RP1A-3 and RP3ABN-1, as observed from Figure 9b, have the optimal HVL. The addition of As_2_O_3_ NPs raises the interaction through the photoelectric effect, leading to the decrease in HVL. However, the addition of BN nanoparticles raises the attenuation coefficient at all energy levels, especially for the hybrid nanocomposites (RP2ABN-X). RP2A-3, a nanocomposite of only As_2_O_3_ NPs, is optimal, proving to be best among all, interacting effectively because of the presence of high-atomic-number materials. RP2ABN-1, RP3ABN-1, and RP2A-3, among the copolymers, prove to be most effective, being measured by high LAC value, proving them to be of high attenuation capacity. Although they possess less attenuation capacity compared to high-atomic-number materials such as lead [28] and Tungsten [29], they prove to be quite effective, given the fact that its density is lower compared to the high-atomic-number materials. HVL of RP2ABN-1 and RP3ABN-1 nanocomposites was lower than HVL of copolymers. These HVLs depend on the minimum HVL of lead [28], its high attenuation capacity because of its high density and atomic number. Considering the MAC value, RP2ABN-1, RP3ABN-1, and RP2A-3 have good values per gram. This means that although the nanocomposites have lower densities, they can still provide good protection in mass-normalized measurements. Conversely, the extremely high MAC values for Pb and W are largely due to their much higher densities. The HVL for RP3ABN-1 at 121 keV is essentially twice that of Pb, but it is still a reasonable value given the material’s lightweight nature and biocompatibility. For dose reduction applications, they may be appropriate for low-dose applications where weight, flexibility, or non-toxic properties may be of greater significance than optimal attenuation. RP2ABN-1, RP3ABN-1, and RP2A-3 provide a compelling balance of attenuation power, safety, and flexibility. Although RP2ABN-1, RP3ABN-1, and RP2A-3 lack attenuation properties comparable to lead, they provide a biocompatible, portable version for shielding against gamma rays for diagnostic or wearable applications.

Figure 9c shows the comparison of the HVL values of the investigated nanocomposites with the HVL values of Pb and Fe. The HVL values of Pb and Fe were calculated using XCOM. The weakest is the RPA3 copolymer. Adding As2O3 NPs to the copolymer improves shielding properties. The best nanocomposite here is RP1A-3. The BN NPs added to this mixture also contributed to shielding. While the effect of BN is mostly on strength, it is clear that it provides some energy improvements. Among the nanocomposites with BN addition, RP3ABN-1 yielded the most efficient results. In all materials, the HVL increases with photon energy, consistent with a transition from photoelectric dominance to Compton scattering at 122 keV to 1.4 MeV. The RPE is generally largest at the lowest energy point and decreases by 3–7% at higher energies. These trends confirm both the experiment and the expected physics of photon–matter interactions. At low energies (121.8 keV), the best HVL values are for RPA1-3 and RP3A-3, followed by RP2A-2 and RP2A-3. Good performers with slightly higher thicknesses include RP3A-2 and RP1A-1. In the intermediate energy region (344–964 keV), RP1A-3 continues to dominate with an HVL of 2.65–2.73 cm across 344–964 keV. RP2A-3 and RP3A-3 are also competitive in this group, while RP3A-2 is low–medium. At higher energies (1.1–1.4 MeV), the thinnest shields at 1408 keV are RP2A-3 and RP3A-3, followed by RP3A-2. Maximum attenuation is observed in the RPA2 and RP2A-3 nanocomposites containing the RPA2 copolymer. An examination of the RPE values reveals that the low-energy RPE has a maximum value for the RP3A-3, RP2A-2, RP1A-1, RP1A-3, and RP2A-3 nanocomposites in each nanocomposite group. Significantly lower values are found in several samples at 1408 keV. Overall, RP1A-3 is an ideal low- to medium-energy shield and a promising candidate at 1 MeV. RPA2 (the benchmark) is ideal at the highest energy level studied, and RP2A-3 is a good and versatile shield with excellent efficiency in the MeV region. RP3A-3 provides the thinnest low-energy shielding. RP3A-2 is the most versatile and reproducible of the range. Therefore, the choice between them will depend on the application: the ultra-low HVL versions (RP1A-3; RP3A-3) are ideal for diagnostic ranges, while the RPA2 and RP3A-2/RP2A-4 nanocomposites are capable of shielding at the MeV scale.

**Table 4:** Comparison of nanocomposites and conventional shielding materials (with references).

Material Type	Density (g/cm^3^)	HVL (cm)	Toxicity/Environmental Impact	Mechanical and Structural Properties	Comments/Limitations	References
Lead (Pb)	11.34	1.1–1.3	Highly toxic; strict handling and disposal controls required	Soft/ductile; needs mechanical support or cladding	Highest attenuation/volume; toxicity and weight are major drawbacks	[31,46,47]
Tungsten (W)	19.3	0.9–1.1	Generally lower acute toxicity than Pb; very dense; machining energy-intensive	Very hard/brittle; expensive; high melting point	Outstanding attenuation; cost/weight and brittleness limit large structures	[31,46]
Ordinary Concrete	2.3–2.4 (mix-dependent)	6–7	Non-toxic; easy to source; CO_2_ footprint depends on cement content	Brittle; requires thickness for high-energy photons	Low cost and constructible; bulky for mobile/wearable uses	[31,48,49]
Polymer Nanocomposites (e.g., polymer + WO_3_/Bi_2_O_3_, BaSO_4_, WC, W/Bi)	1.8–3.5 (loading-dependent)	1.2–3.0	Lead-free options; polymer matrices generally low toxicity	Lightweight, flexible, corrosion-resistant; properties tunable by filler type/size/volume	Excellent specific (per-weight) shielding; absolute attenuation limited at very high E if loading is low	[50,51,52,53]
MOF-Containing Composites (MOF/polymer, MOF/textile, etc.)	0.8–2.5 (system-dependent)	2–4 (reported, system-dependent)	Typically low toxicity; potential for multifunctionality (EMI adsorption, sorption)	Porous/engineerable; enables multifunctional designs	Evidence base emerging; scale-up and radiation tolerance vary by MOF	[51,54]
RP1A-2 (70 wt% As_2_O_3_ + 30 wt% PS-b-PEG (1000))	2.04	4.64 cm at 778.9 KeV	Non-toxic, recyclable	Porous, multifunctional (EMI + radiation) Easy to shape and fabricate	have lower absolute shielding at high energies	This study
RP1A-3 (90 wt% As_2_O_3_ + 10 wt% PS-b-PEG (1000))	3.34	2.73	“	“	“	This study
RP2A-3 (90 wt% As_2_O_3_ + 10 wt% PS-b-PEG (1500))	2.61	4.39	“	“	“	This study
RP3A-3 (90 wt% As_2_O_3_ + 10 wt% PS-b-PEG (10,000))	2.42	3.70	“	“	“	This study
RP2ABN-1 (70 wt% As_2_O_3_ + 15 wt% BN + 15 wt% PS-b-PEG (1500))	2.26	4.65	“	“	“	This study

Note: HVL values of the materials in Table 4 at 662 keV are typical ranges derived from NIST XCOM data and standard handbooks; actual values vary with composition, moisture, porosity, and filler loading. HVL values of the nanocomposites in Table 4 are at 778.9 KeV.

##### The Calculating of TVL Values of the Nanocomposites

**Figure 10:**
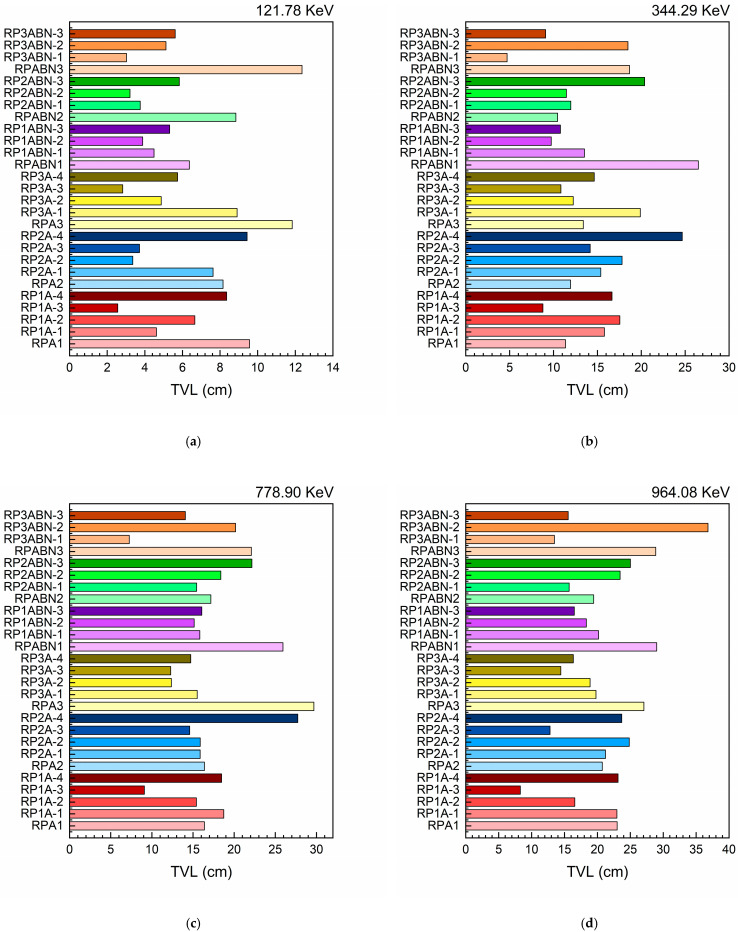

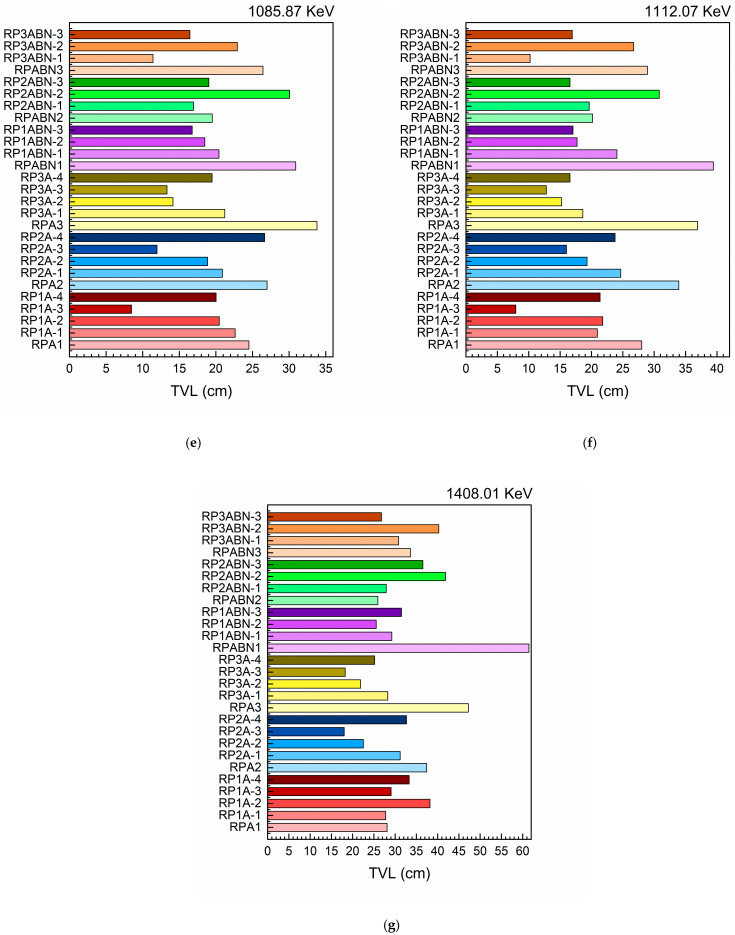
TVL (cm) of As_2_O_3_- and BN-based PS-PEG copolymers: (**a**); 121.78 keV (**b**); 344.29 keV (**c**); 778.90 keV (**d**); 964.08 keV (**e**); 1085.87 keV (**f**); 1112.07 keV (**g**); 1408.01 keV.

The graphs in Figure 10 show the TVL values of the nanocomposites. In this study, the radiation attenuation performances of nanocomposites were comparatively evaluated based on TVL values measured at different energy levels (121.8–1408.0 keV). Increasing TVL indicates that a material has lower attenuation efficiency at that energy level. Therefore, lower TVL values are associated with better radiation absorption behavior.

The RP1A-3 nanocomposite demonstrated the best performance in the low-energy region. The TVL value of 2.56 recorded at a photon energy of 121.8 keV demonstrates that RP1A-3 can effectively absorb low-energy photons. Because this value is the lowest TVL among all samples in Table S4, RP1A-3 provides the most efficient shielding in the low-energy range. In the medium-energy range, the RP3ABN-1 nanocomposite appears to stand out. TVL values of 4.71 cm and 7.26 were obtained at 344.3 keV and 778.9 keV, respectively. These findings clearly denote that RP3ABN-1 possesses a relatively higher capacity for photon energy absorption. This finding supports that the RP3ABN-1 nanocomposite possesses a shield capability optimized for this energy range. The RP1A-3 nanocomposite for high energy again displayed prominent performance. The TVL was measured to be 8.30 cm, 8.47 cm, and 7.94 cm for 964.1 keV, 1085.9 keV, and 1121.1 keV, respectively. These findings denote that the RP1A-3 possesses a highly stable and efficient performance for high-energy photon attenuation, keeping the efficiency unchanged, especially when the value of TVL again dropped to 7.94 cm for 1121.1 keV.

The TVL value of the RP2A-3 nanocomposite at 1408.0 keV, the highest energy level examined in the study, is 18.02 cm. That is a high TVL, indicating that RP2A-3 composites require a thick shield for high-energy photons, and its ability to be absorbed is less efficient than other composites for this energy range. This suggests that RP2A-3 is not an effective material in the high energy range. Generally, RP1A-3 exhibits the best radiation attenuation performance in the low and high energy ranges, and RP3ABN-1 nanocomposites in the medium-energy range. The performance of RP2A-3 decreased significantly at very high energies. These results indicate that the type of dopant used, matrix structure, and nanocomposite morphology significantly affect attenuation behavior depending on the energy.

Dahinde et al. studied the radiation-shielding properties of MnO_2_, CuO, Al_2_O_3_, and MgO for 122 keV and 1330 keV energy photons [33]. The experimental TVL for 122 keV energy photons was measured to be 1.1905 cm for Al_2_O_3_, 1.3276 cm for MnO_2_, 0.4038 cm for CuO, and 0.6793 cm for MgO, whereas, theoretically, the value was found to be 1.1744 cm, 1.2919 cm, 0.3979 cm, and 0.6694 cm for Al_2_O_3_, MnO_2_, CuO, and MgO, respectively. CuO had the lowest photon attenuation efficiency of 90%, TVL of 0.4038 cm, whereas MnO_2_ had the lowest attenuation efficiency of 2.4989 cm for 1330 keV energy photons. The experimental TVL for 1330 keV energy photons was found to be 2.4989 cm for Al_2_O_3_, 4.0057 cm for MnO_2_, 2.5788 cm for CuO, and 2.9231 cm for MgO. The theoretically calculated value was found to be a little higher, 3.5484 cm for Al_2_O_3_ and 3.9286 cm for MnO_2_. The increase in TVL with increase in energy is due to the decrease in photon interaction probability. Once again, MnO_2_ is found to be the lowest attenuating material, needing the highest thickness, whereas CuO was a medium performer. The experimental value of TVL showed a remarkable difference in energy-dependent attenuation capacity among the different nanocomposites. The general trend was that television value was lower at lower energy, whereas television value was high for high energy, due to decrease in photon interaction probability. This result is well supported by the experimental findings of Dahinde et al. for MnO_2_, CuO, Al_2_O_3_, and MgO oxide materials. Dahinde and colleagues reported TVL values at 122 keV as 1.1905 cm for Al_2_O_3_, 1.3276 cm for MnO_2_, 0.4038 cm for CuO, and 0.6793 cm for MgO. They stated that CuO provided the highest attenuation efficiency with its lower TVL value. This energy level, compared to the 2.56 cm TVL value for the RP1A-3 nanocomposite at 121.8 keV in our study, demonstrates that the oxide-based materials alone have higher absorption capacity, but the nanocomposite structures exhibit higher TVL values due to different matrix–filler interactions. Furthermore, the fact that RP1A-3 exhibits the best performance in the lower energy region in our study demonstrates that optimization of the composite structure significantly impacts material-based differences. A similar trend is observed at higher energy levels. Dahinde et al. reported TVL values of 2.4989 cm, 4.0057 cm, 2.5788 cm, and 2.9231 cm for Al_2_O_3_, MnO_2_, CuO, and MgO samples, respectively, at 1330 keV, and reported the lowest attenuation efficiency for MnO_2_. These results indicate that all oxides exhibit higher TVL values with increasing energy. In our study, at the closest energy range, 1408.0 keV, the RP2A-3 composite was determined to require the highest thickness, with a TVL of 18.02 cm. Compared to Dahinde’s oxide systems, filler–matrix interactions are weakened in the nanocomposite structure, particularly at very high energies, and pure oxides are more effective attenuators. However, the fact that the best energy-dependent performance in our nanocomposite systems varies depending on the energy region between RP1A-3 and RP3ABN-1 suggests that the nanocomposite design operates through a more complex mechanism, distinct from the individual behavior of the oxides. The optimal performance of RP3ABN-1 in the mid-energy range is similar to the moderate performance of CuO in Dahinde’s studies, indicating that the role of metal oxide structures in the composite varies with energy.

Overall, both in this study and the data reported by Dahinde et al., it is clear that TVL increases with increasing energy, thus decreasing the probability of photon interaction. While Dahinde’s oxide-based systems offer more efficient shielding performance with lower TVL values at higher energies, in our nanocomposite systems, performance was found to be strongly correlated with filler type, concentration, and matrix structure. These results demonstrate that nanocomposite design must be optimized for specific energy requirements and that it is not possible to directly transfer the behavior of individual oxide materials to composite systems. In the RP1ABN, RP2ABN, and RP3ABN nanocomposites, despite the decrease in As_2_O_3_ NPs and the increase in BN, the absorption values remain nearly equivalent to the good TVL values mentioned above. When we examine the BN-doped sets in particular, the RP3ABN-1 composite yields the best values at all energies except 1408 keV. Despite its lower As_2_O_3_ content than the RP1A-3 composite, it was observed to provide acceptable absorption values. The lowest HVL and TVL values were observed for RPA3 at 121.78 keV, followed by RPA1 and RPA2. The homogeneous composites yielded significantly higher HVL and TVL values at 121.78 keV. The BN-doped materials demonstrated moderate shielding capabilities. The RP3ABN-1, RP2ABN-2, and RP1ABN-3 composites stand out among the BN-doped samples in terms of their performance. Among the nanocomposites, RP1A-2, RP1A-3, RP2ABN-3, and RP3ABN-3 performed similarly to or better than barite concrete in terms of HVL [55]. The studied nanocomposites performed comparably better than conventional materials [38,45]. RP1A-3, RP2ABN-3, and RP3ABN-3 nanocomposites performed close to barite concrete in terms of TVL.

As a result, the RP1A-3 composite provides the best protection at 121.78, 344.29, 778.90, and 964.08 keV. RP2A-3 provides the best TVL value at 1085.87 and 1408.01 keV (Table S4). RP1A-3, RP2A-3, and RP3A-3 nanocomposites provide the most effective radiation protection. These composites contain 90% As_2_O_3_ (Table 1).

When As_2_O_3_ and BN NPs are combined together, RP3ABN-1 is found to have the best performance among all the nanocomposites, containing 15 wt% PS-b-PEG (10,000), 15 wt% BN, and 70 wt% As_2_O_3_. This hybrid nanocomposite is found to have the largest MAC value for all cases (0.423 cm^2^ g^−1^ at 121.78 keV) and also possesses higher values of LAC (0.761 cm^−1^) than BN-doped samples and larger values of RPE. When the proportion of As_2_O_3_ NPs are decreased from 90 wt% to 70 wt%, HVL and TVL values remain small for energies higher than 121.78–1112 keV and are very close to values for As_2_O_3_-rich composites. This particular combination shows a genuine synergistic effect because As_2_O_3_ NPs acts as high-Z material with strong γ-photon interaction, while BN acts as follows: (i) enhances thermal stability and additionally enhances structural stability too, (ii) also helps to retain its dense microstructure without any cracks (SEM/TEM analysis is required to confirm) for all combinations.

##### The Calculating of MFP Values of the Nanocomposites

**Figure 11:**
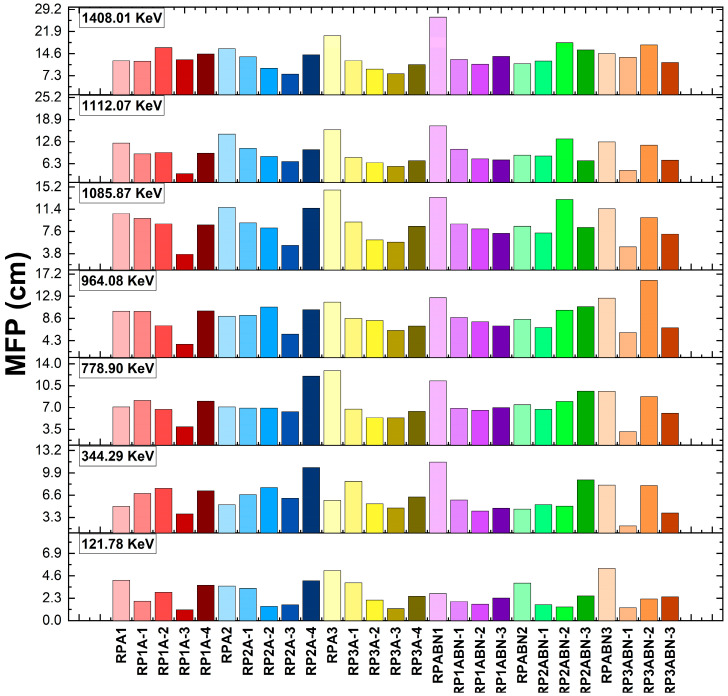
MFP (cm) of the PS-PEG copolymers and the nanocomposites.

The graphs in Figure 11 show the MFP values of the copolymers and the nanocomposites. When the energy-dependent mean free paths of the nanocomposites tested in the study are analyzed, it is found that RP1A-3 shows the best attenuation ability at lower energy levels. At a photon energy of 121.8 keV, the RP1A-3 composite exhibited the lowest MFP value in the table with an MFP value of 1.11 cm, indicating that the material has a high probability of interaction with low-energy photons. In the medium-energy range, RP3ABN-1 nanocomposite is remarkable. The value of 2.05 cm for the MFP at 344.3 keV shows that this particular sample is better than the others, as far as photon absorption is concerned, for medium energy. This value confirms that the photon–matter interaction decreases as expected and the MFP increases as the energy increases. At higher energy levels, the RP1ABN-1 sample exhibited significant performance.

The MFP value of 3.15 cm for this composite at 778.9 keV indicates that photon interaction activity persists in the medium- to high-energy range. Continuing to increase the energy, the RP1A-3 composite was again found to be predominant, yielding RP1A-3 MFP values of 3.60 cm, 3.68 cm, and 3.45 cm for 964.1 keV, 1085.9 keV, and 1121.1 keV, respectively. These findings have made it quite evident that the RP1A-3 sample experiences a quite steady attenuation process for high-energy photons. Corresponding to the highest energy of 1408.0 keV, the RP2A-3 composite was found to have the highest value of 7.82 cm for the MFP. The high value reveals that RP2A-3 experiences lower interaction for high-energy photons, thus requiring a greater thickness of RP2A-3 for efficient attenuation.

Generally, while RP1A-3 offers more effective attenuation performance at low energies, RP3ABN-1 in the mid-energy range, and RP1ABN-1 in the mid- to high-energy region, the attenuation effectiveness of RP2A-3 decreases significantly at the highest energies. These results demonstrate that nanocomposites exhibit different interaction mechanisms depending on energy, and that composite design plays a critical role in radiation shielding. This value confirms that the photon–matter interaction decreases as expected and the MFP increases as the energy increases. At higher energy levels, the RP1ABN-1 sample exhibited significant performance. The MFP value of 3.15 cm for this composite at 778.9 keV indicates that photon interaction activity persists in the medium- to high-energy range. Continuing to increase the energy, a dominance of the RP1A-3 composite was observed, giving MFP values of 3.60 cm, 3.68 cm, and 3.45 cm for 964.1 keV, 1085.9 keV, and 1121.1 keV, respectively. It is made apparent that the RP1A-3 possesses a fairly constant attenuation property against high-energy photons.

The energy-dependent MFP values measured from this research make it clear that the interaction behavior of photons to the nanocomposites differs extensively according to the energy range. When the energy range was low (121.8 keV), the RP1A-3 had the lowest MFP of 1.11 cm, proving to be the most efficient among the samples because of its ability to interact well even at low-energy photons. Examining Dahinde et al.’s 122 keV findings corresponding to the same energy level, it is seen that MgO exhibited the shortest MFP value of 0.5826 cm, while CuO had the lowest interaction length of 0.9802 cm. In this comparison, it can be said that the pure oxides in Dahinde’s study exhibited higher photon interaction efficiency at low energy compared to our nanocomposites [33]. This difference can be explained by the fact that the organic phase in the nanocomposite matrix structure, despite the high-atomic-number density of the oxides, somewhat reduces the interaction probability. The RP3ABN-1 sample differs from the others, being unique because of its MFP of 2.05 cm at medium energy, namely 344.3 keV, signifying that this composite is better at absorbing medium-energy photons. This is comparable to the trend found by Dahinde et al. for oxide materials, where as energy increases, the MFP values of all materials increase, and the probability of photon–matter interactions decreases. Indeed, in Dahinde’s study, the MFP values of the oxides at 1330 keV were measured in the range of 3.6–5.7 cm; these values are significantly higher than the MFP values of the nanocomposites in the medium-energy region in our study. Therefore, it appears that the filler–matrix interaction is more effective in nanocomposites than in oxides at certain energy ranges, with RP3ABN-1, in particular, providing relatively more intense interactions in the medium-energy region. In the high-energy region (778.9–1121.1 keV), the MFP values of RP1ABN-1 and RP1A-3 samples, ranging from 3.15 to 3.68 cm, indicate that the nanocomposites still exhibit stable attenuation behavior against high-energy photons. The value of the MFP for 1330 keV is given as 4.9988 cm for Al_2_O_3_, 5.7102 cm for MnO_2_, 4.2459 cm for CuO, and 3.7054 cm for MgO. These values, compared to our nanocomposite results, indicate that MgO has the shortest MFP value at high energy. This demonstrates that pure oxides have more intense scattering and absorption mechanisms, especially at higher energies. The RP2A-3 nanocomposite exhibits a very high MFP value of 7.82 cm at the highest energy level (1408 keV), demonstrating that this material has a low interaction probability with very high-energy photons. The MFP values in Dahinde’s oxides at levels close to this energy range, in the range of 3.6–5.7 cm, confirm that oxides are more efficient attenuators than nanocomposites at higher energies. In particular, MgO exhibits short MFP values at both low and high energy levels, demonstrating that this oxide provides reliable shielding performance across all energy ranges.

Furthermore, from the study conducted by Dahinde et al., it is observed that the CuO had the lowest HVL and TVL for all energy intervals, such as HVL of 0.347 cm at 1275 keV energy and TVL of 0.4038 cm at 122 keV energy. This phenomenon is because of its high atomic density, thus greater attenuation power. This observation is comparable to our findings, where RP1A-3 performed better at low energy, and RP3ABN-1 and RP1ABN-1 performed better at medium and high energy. It is important to state that the findings of both this study and that of Dahinde et al. [33] make a compelling case for the fact that mean free path length increases, and the interaction probability of photons reduces along with the increase in energy. Although the findings from this study clarify that the oxide materials provide better protection for shorter mean free path lengths, especially at high energy, another thing is that the interaction of materials and filler within the nanocomposite is much more complicated due to the energy levels.

##### The Calculating of RPE Values of the Nanocomposites

**Figure 12:**
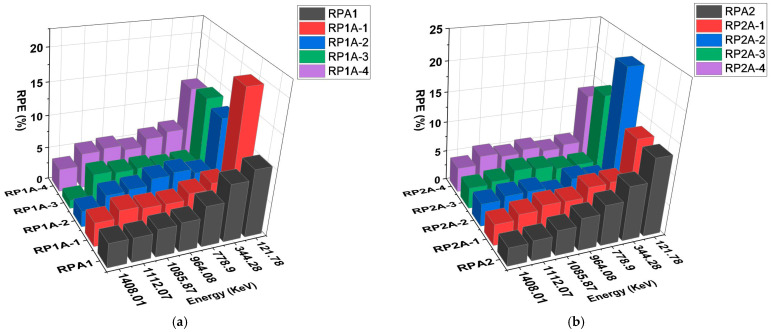

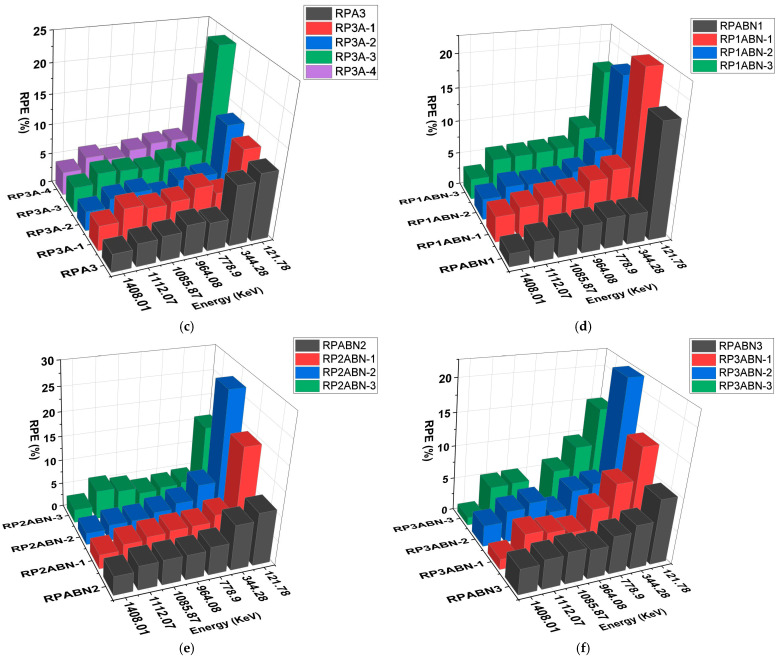
RPE (%) of As_2_O_3_- and BN-based PS-PEG copolymers: (**a**); RPA1, RP1A-1, RP1A-2, RP1A-3, RP1A-4. (**b**); RPABN1, RP1ABN-1, RP1ABN-2, RP1ABN-3. (**c**); RPA2, RP2A-1, RP2A-2, RP2A-3, RP2A-4. (**d**); RPABN2, RP2ABN-1, RP2ABN-2, RP2ABN-3. (**e**); RPA3, RP3A-1, RP3A-2, RP3A-3, RP3A-4. (**f**); RPABN3, RP3ABN-1, RP3ABN-2, RP3ABN-3.

RPE measurements can be examined as a result of pellet measurements, as in MAC. The RPE calculated as given in Equation (6) shows the percentage decrease in the initial photon number. Figure 12 graphs examine the radiation-shielding efficiencies of pellets within their own groups of nanocomposites. Pellet thicknesses should also be taken into account when examining the Figure 12 graphs.

The value of RPE obtained shows a considerable decrease in the attenuation ability of the nanocomposites as a result of the increase in the energy of the gamma photons. When the energy of the photons is 121.8 keV, the highest value of RPE was obtained for RP2ABN-2 nanocomposite, namely 27.20%, within the range of this study. The systematic decrease in attenuation efficiency was found to be proportional to the increase in the energy level; thus, RPE of 9.89% was obtained for RPABN3 at 344.3 keV, 7.28% for RP3A-1 at 778.9 keV, and 5.92% for RP3A-4 at 964.1 keV. However, for high-energy photons, the attenuation trend reduced considerably, such that the lowest was found to be for RP1ABN-3 nanocomposite at 1085.9 keV for RPE of 5.80%, for RP3A-1 at 1121.1 keV for RPE of 6.10%, and finally at 1408.0 keV, that is, the highest energy level of this investigation, for RP3A-3 nanocomposite, where the value of RPE was 4.19%, respectively. These experimental observations strictly reveal that, for the nanocomposites, the ability to absorb high-intensity photons is less because of the dominance of Compton scattering, whereas for high-intensity photons, their efficiency is limited because of the dominance of Compton scattering.

When examined together with the thicknesses in Table 1, the effectiveness of the RP1A-3 nanocomposite is seen as in the MAC comments. When all measurements and analyses are examined, nanocomposites with high As_2_O_3_ NPs content show high RPE. On the other hand, it was observed that BN-doped nanocomposites contributed to radiation efficiency, and RP1ABN-3, RP2ABN-2, RP3ABN-1, and RP3ABN-3 nanocomposites with relatively low As_2_O_3_ NPs content compared to others also showed strong RPE value.

In our study, high-Z As_2_O_3_ NPs are the major contributory material to γ-ray attenuation, while BN primarily adds to stability and supports the maintenance of an efficient microstructure for good shielding. Together as NPs, their role is undoubtedly synergistic. Formulations containing 70 wt% As_2_O_3_ NPs and 10–15 wt% BN NPs and composed of PS-b-PEG as the remaining components emerge as optimum on the basis of the present result.

These nanocomposites possess MAC, LAC, HVL, and TVL values equivalent to or arguably better than any As_2_O_3_-rich composition but have been formulated to possess better stability and to facilitate better processability for use as protection components.

When only As_2_O_3_NPs are added to PS-b-PEG, MAC is raised and becomes higher with higher concentrations of As_2_O_3_, while LAC is also raised but to a lesser extent than MAC values for all energy points. Then, for all three types of copolymer matrixes (PEG 1000, 1500, and 10,000 molecular weight matrixes), formulations RP1A-3, RP2A-3, and RP3A-3 containing 90 wt% As_2_O_3_ NPs have higher MAC and LAC while having smaller HVL/TVL values for all energy points studied than any of the samples containing modulators but no As_2_O_3_ NPs or any of its compositions, confirming As_2_O_3_ NPs to have dominated gamma attenuation mainly through photoelectric and Compton scattering processes. The addition of BN to the copolymer (e.g., RPABN1 containing 50 wt% BN) enhances MAC from 0.235 to 0.293 cm^2^ g^−1^ at the 121.78 keV energy point itself, though BN’s atomic number is substantially lower than As_2_O_3_. This implies that BN also helps to reduce photons to some extent at low to medium energy, mostly through enhanced apparent densities and tortuous paths generated by its plate-like morphology. The values of MAC for all BN-containing samples (RP1ABN series, RP2ABN series, and RP3ABN series) show reliable and sustained performance at all energy points and display significantly enhanced thermal stability measures by showing substantial residue percentages at TGA (e.g., 66 wt% residue for RPABN1 at the third stage of analysis).

## 4. Conclusions

This study focuses on the gamma radiation shieldings of PS-PEG nanocomposites reinforced with As_2_O_3_, BN, and the combined form of As_2_O_3_/BN. The outcomes of this study clearly show that the hybrid nanocomposites RP1A-3 and RP3ABN-1 possess better gamma radiation shielding than the other PS-PEG nanocomposites and the existing results of various other polymers used as gamma radiation shielding. This clearly indicates that the As_2_O_3_/BN-reinforced PS-PEG nanocomposites possess the highest MACs and the lowest values of HVL, TVL, and MFP over the higher photon energies (121–1408 keV).

RP1A-3 and RP3ABN-1 polymer composites (with BN and As_2_O_3_ nanoparticles) have the highest mass attenuation coefficient (MAC) values at all tested photon energies, demonstrating superior performance even after intensity normalization. These composites significantly increase gamma-ray absorption capacity compared to the base copolymers; for example, the HVL for RP1A-3 are 0.77 cm (121.78 keV), 2.65 cm (344.28 keV), 2.73 cm (964.08 keV), 2.39 cm (1112.07 keV), and 8.74 cm (1408.01 keV). RPA1, RPA2, RPA3, RPABN1, RPABN2, and RPABN3 exhibit the lowest MAC and worst performance. In the low- to medium-energy range (121.78–1112.07 keV), RP1A-3 and RP3ABN-1 provide excellent attenuation with low HVL values comparable to conventional metals such as lead (density 11.34 g/cm^3^) and tungsten (19.25 g/cm^3^). Among the RPE series, these composites provide the highest value of relative shielding effectiveness than base systems. Their lightweight, biocompatible, and non-toxic structure make them ideal candidates for diagnostic radiology, portable devices, and personal protection; variants such as RP3ABN-1 and RP2ABN-2 are adequate for low- to medium-energy gamma applications.

In conclusion, MAC and LAC reduce monotonically for increasing photon energy until 121.78 keV. The combined action of As_2_O_3_ and BN NPs makes RP3ABN-1 most effective for all but the lowest portions of the spectrum. The photoelectric processes become dominant at very low energies because of its high-Z filler As_2_O_3_ NPs, while Compton scattering dominates at intermediate to high energies up to 1408 keV. Pair production is unimportant for these composites for all energy values considered. In our nanocomposites, BN NPs not only substitute for part of high-Z As_2_O_3_ NPs but also change the interaction of the shield material with the particles and its microstructure. This improvement makes PS-PEG/As_2_O_3_/BN hybrids superior to As_2_O_3_ NP-doped composites at 121–778 keV.

The PS-PEG/As_2_O_3_/BN systems are better than traditional metal shields for portable, flexible, and aerospace shielding because they have a low density and a high attenuation rate. Table 1 shows that the densities of our best formulations (RP1A-3, RP2A-3, RP3A-3) are between 2.04 and 3.34 g·cm^−3^, and the HVL values are between 2.73 and 4.64 cm at 778.9 keV. When weighed, our composites have specific shielding performance capabilities better than normal concrete and equivalent to lead (Pb). They also have the advantages of being lightweight, resistant to corrosion, and easy to work with because of the polymer matrix. In practical terms, these traits mean that the composites can be made into flexible sheets or thin laminates, shaped and drilled without the problems that come with W, and used in lightweight structures where weight is important (like portable/wearable protectors, aerospace panels, and housings for imaging detectors). The good thermal stability of PS-b-PEG and the fact that As_2_O_3_/BN NPs are not a metal suggest that these materials will still work well as shields even after long periods of radiation exposure and moderate temperature changes, which are common in medical imaging and aircraft/spacecraft cabins. Nevertheless, further testing is needed for these materials to fully certify them for use in such applications. The innovation of the proposed study arises due to the combined effects of As_2_O_3_ and BN as dual NPs of the PS-PEG matrix that promotes gamma-ray absorption without utilizing highly dense metals like lead or tungsten. Also, the proposed composite materials appear lighter and more biocompatible and non-toxic compared to conventional gamma-ray protection materials that contain dense materials like lead or tungsten. Specifically, the proposed study examined that the optimal performance of the shielded materials occurs at low and medium energies with the shielded material of the RP3ABN-1 type, whereas the shielded material of the type RP1A-3 indicated highly efficient protection performance on all measured conditions. To further justify the gamma-ray protection properties of the proposed materials, the proposed study examined the XRD results of the materials that indicated the crystal structures of the proposed nanocomposite materials with a distinct structure that validates the inclusion of NPs with stability at the required levels throughout the matrix of the proposed materials. Finally, the proposed study brings the first suggestive results of the inclusion of PS-PEG/As_2_O_3_, PS-PEG/As_2_O_3_/BN combined materials concerning gamma-ray protection performance that validates the proposed materials as an innovation of currently available PS-PEG/As_2_O_3_/BN gamma protection analogs. As evidence of the shielding phenomenon, XRD results showed that the nanocomposites possess a crystal structure with uniform distribution of NPs within the matrix with minimal incorporation effects on the nanoparticles’ structure.

Further studies on neutron attenuation, long-term stability, and mechanical characterization of optimal composites are also necessary to assess their applicability under real-world radiation exposures. RP3ABN-1 is the most effective shielding material for gamma radiation, particularly at low energies. RP3ABN-1 and RP1A-3 offer excellent balances between attenuation efficiency and physical/thermal properties. They are suitable for medical imaging protection, wearable protective clothing, aerospace radiation protection, and portable radiation protection in nuclear environments. This paper presents a new class of polymer-based nanocomposites that innovatively blends polymer chemistry and radiation physics, offering a safer and lighter alternative to traditional materials such as lead and tungsten for gamma protection.

## Figures and Tables

**Table 1:** The composition of the nanocomposites.

	Nanocomposite ID	PS-b-PEG Type	PS-b-PEG (wt%)	BN (wt%)	As_2_O_3_ (wt%)	Pellet Thickness d (cm)	Density (gr/cm^3^)
1	RPA1	1000	100	0	0	0.448	1.03
2	RP1A-1	1000	50	0	50	0.437	1.68
3	RP1A-2	1000	30	0	70	0.401	2.04
4	RP1A-3	1000	10	0	90	0.165	3.34
5	RP1A-4	1000	46.2	0	53.8	0.512	1.69
6	RPA2	1500	100	0	0	0.492	1.00
7	RP2A-1	1500	50	0	50	0.477	1.65
8	RP2A-2	1500	30	0	70	0.373	2.00
9	RP2A-3	1500	10	0	90	0.277	2.61
10	RP2A-4	1500	46.2	0	53.8	0.593	1.71
11	RPA3	10,000	100	0	0	0.613	1.10
12	RP3A-1	10,000	50	0	50	0.509	1.74
13	RP3A-2	10,000	30	0	70	0.31	2.20
14	RP3A-3	10,000	10	0	90	0.339	2.42
15	RP3A-4	10,000	46.2	0	53.8	0.433	1.79
16	RPABN1	1000	50	50	0	0.525	1.23
17	RP1ABN-1	1000	15	15	70	0.49	2.11
18	RP1ABN-2	1000	5	5	90	0.353	2.43
19	RP1ABN-3	1000	26.1	13	60.9	0.435	1.97
20	RPABN2	1500	50	50	0	0.443	1.72
21	RP2ABN-1	1500	15	15	70	0.358	2.26
22	RP2ABN-2	1500	5	5	90	0.443	2.12
23	RP2ABN-3	1500	26.1	13	60.9	0.443	1.77
24	RPABN3	10,000	50	50	0	0.559	1.31
25	RP3ABN-1	10,000	15	15	70	0.209	1.80
26	RP3ABN-2	10,000	5	5	90	0.559	1.17
27	RP3ABN-3	10,000	26.1	13	60.9	0.405	2.19

**Table 3:** The best performance of the nanocomposites.

Best Performers (Low Energy)	Trend	Notable Insight
RPABN1, RPA2	$\mu_{m}$ peaks at 121.78 keV	RPABN1 composites enhance attenuation marginally
RP2A-3, RP3ABN-3	Moderate to high $\mu_{m}$	Enhanced shielding from RP1ABN-3
RP3ABN-1	Highest $\mu_{m}$ overall	Most efficient shielding copolymers in the study

## Data Availability

The data presented in this study are available on request from the corresponding author.

## Supplementary Materials

The following supporting information can be downloaded at: https://www.mdpi.com/article/10.3390/polym17243330/s1, Figure S1: (a-b) SEM images (c) EDX (Map Sum Spectrum) graph and (d) EDS Layered Image of RP1A-1 nanocomposite, Figure S2: (a-b) SEM images (c) EDX (Map Sum Spectrum) graph and (d) EDS Layered Image of RP2A-1 nanocomposite, Figure S3: (a-b) SEM images (c) EDX (Map Sum Spectrum) graph and (d) EDS Layered Image of RP3A-1 nanocomposite, Figure S4: TEM pictures (a-b, d-g) and EDS Results (c) TEM images of of RP1A-2, Figure S5: (a-f) TEM pictures of RP2A-2, Figure S6: (a-f) TEM pictures of RP3A-2, Figure S7: Origin graphs plotted for three separate measurements of sample RP1A-3, Figure S8: RP3A-1 sample peak analysis for 121.78 KeV (a) Peak area for measurement without sample (b) First RP3A-1 measurement (c) Second First RP3A-1 measurement (d) Third RP3A-1 measurement, Figure S9: RP3A-1 sample peak analysis for 344.28 KeV (a) Peak area for measurement without sample (b) First RP3A-1 measurement (c) Second First RP3A-1 measurement (d) Third RP3A-1 measurement, Figure S10: RP3A-1 sample peak analysis for 778.90 (Peak index 1), 964.08 (Peak index 2), 1085.87 (Peak index 3), 1112.07 (Peak index 4), 1408.01 (Peak index5) KeV (a) Peak area for measurement without sample (b) First RP3A-1 measurement (c) Second First RP3A-1 measurement (d) Third RP3A-1 measurement, Figure S11: XRD graphs of RP1A-1, RP2A-1, RP3A-1, RP1ABN-1, RP2ABN-1 and RP3ABN-1 nanocomposites, Table S1: TGA results of the nanocomposites. Table S2: Experimental and XCOM *µ*_m_ Values of the nanocomposites, Table S3: Experimental and XCOM *µ*_L_ Values of the Nanocomposites, Table S4: RPE(%), HVL, TVL, MFP Values of the Nanocomposites, Table S5: XRD value, Table S6: The amount of deviation between the peak areas obtained for RP3A-1 and the average of these areas.
