# Supplementary material for: Gamma Radiation Shielding Efficiency of Cross-Linked Polystyrene-b-Polyethyleneglycol Block Copolymer Nanocomposites Doped Arsenic (III) Oxide and Boron Nitride Nanoparticles
Source: Polymers (Basel). 2025 Dec 17;17(24):3330. doi: 10.3390/polym17243330 (PMC12737299; doi:10.3390/polym17243330)
Supplement: Supplementary file 1 [file polymers-17-03330-s001.zip › polymers-3972644-supplementary.pdf]

Table S1. TGA results of the nanocomposites.

PCM ID	FIRST STAGE OF DEGRADATION		SECOND STAGE OF DEGRADATION		THIRD STAGE OF DEGRADATION	
	t ºC	Remaining Mass %wt	t ºC	Remaining Mass %wt	t ºC	Remaining Mass %wt
RP1A-1	215.4	97.3	444.75	0.91	-	-
RP2A-1	221.91	97.66	425.59	1.19	521.84	0.29
RP3A-1	147.35	99.61	333.26	60.88	445.23	2.05
RP1ABN-1	224.34	98.36	375.86	33.85	-	-
RP2ABN-1	39.67	99.12	239.49	92.55	442.08	66.52
RP3ABN-1	232.76	97.85	388.94	17.91	602.76	16.51

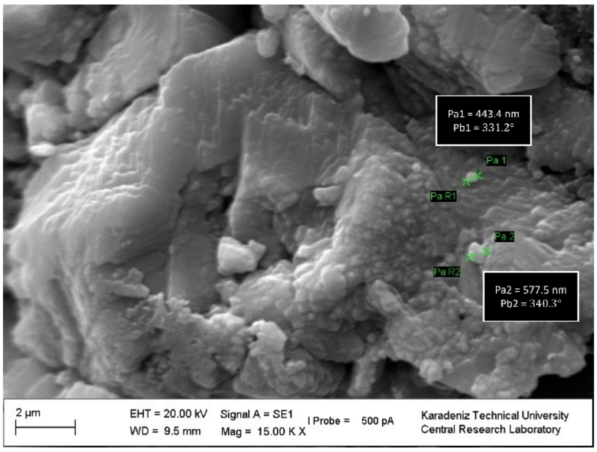

(**a**)

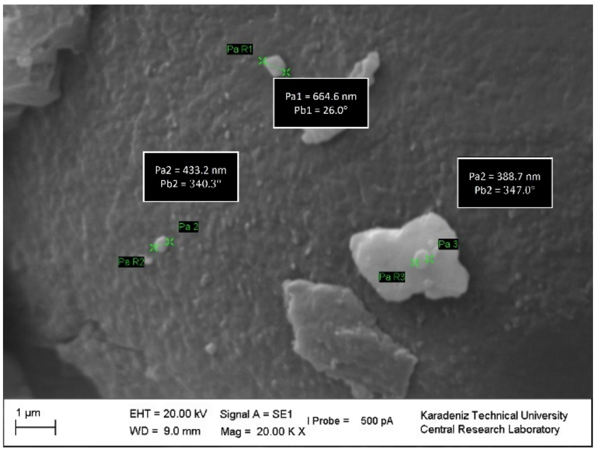

(**b**)

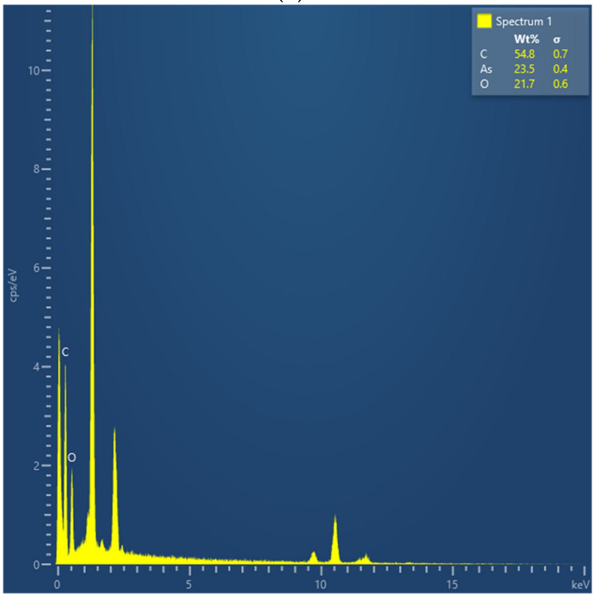

(**c**)

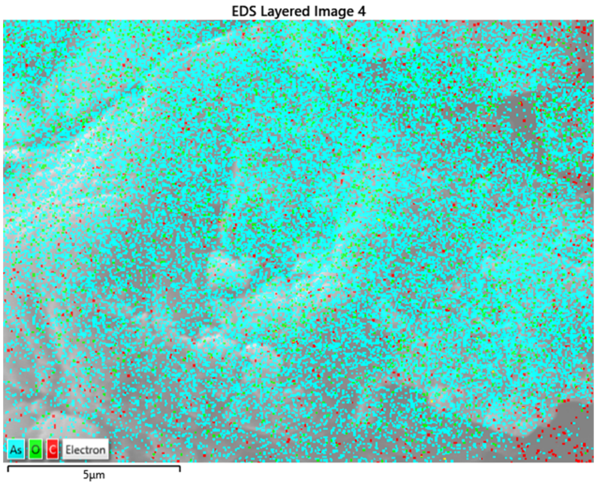

(**d**)

Figure S1. (a-b) SEM images **(c)** EDX (Map Sum Spectrum) graph and **(d)** EDS Layered Image of RP1A-1 nanocomposite.

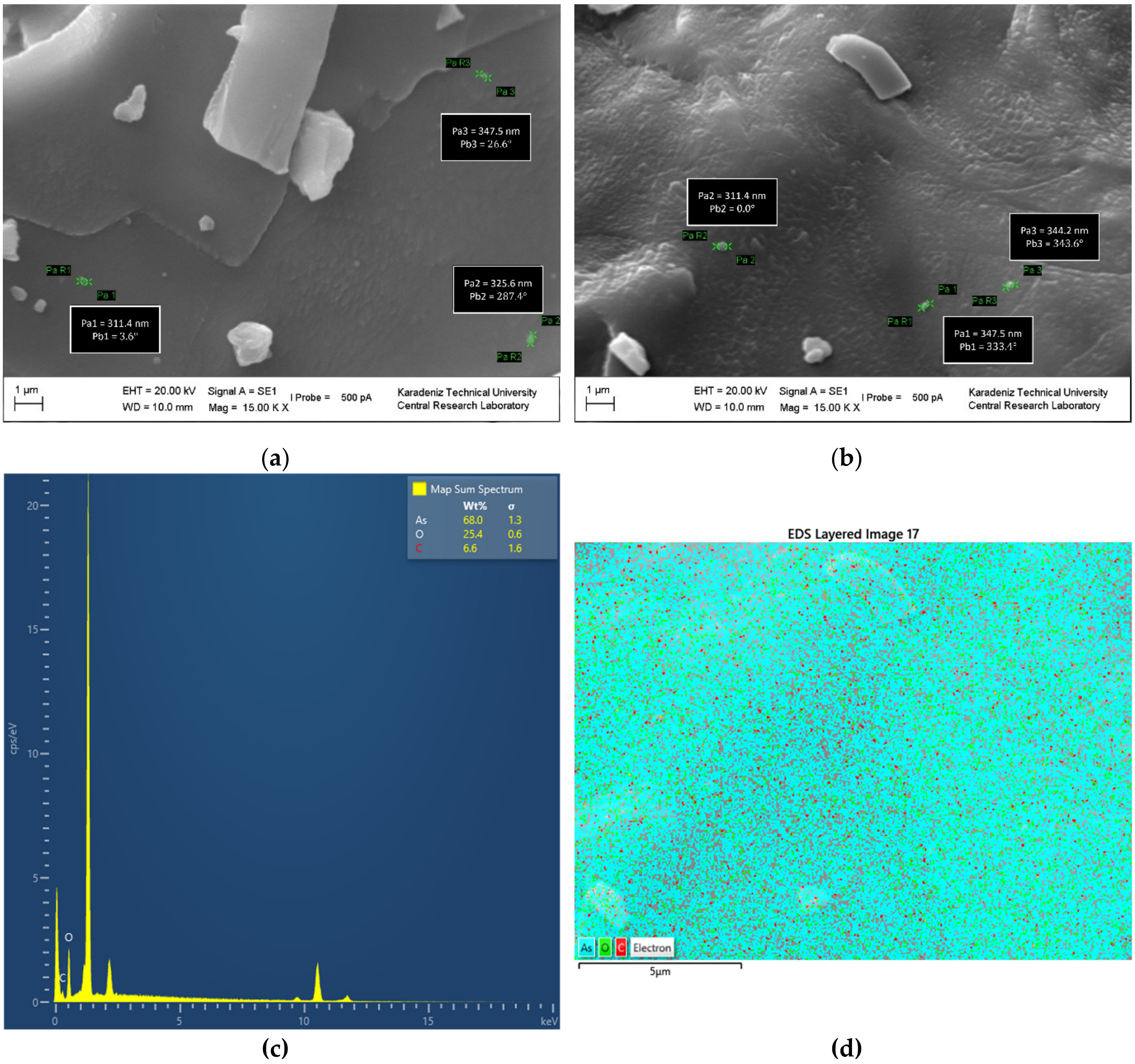

Figure S2. (a-b) SEM images **(c)** EDX (Map Sum Spectrum) graph and **(d)** EDS Layered Image of RP2A-1 nanocomposite.

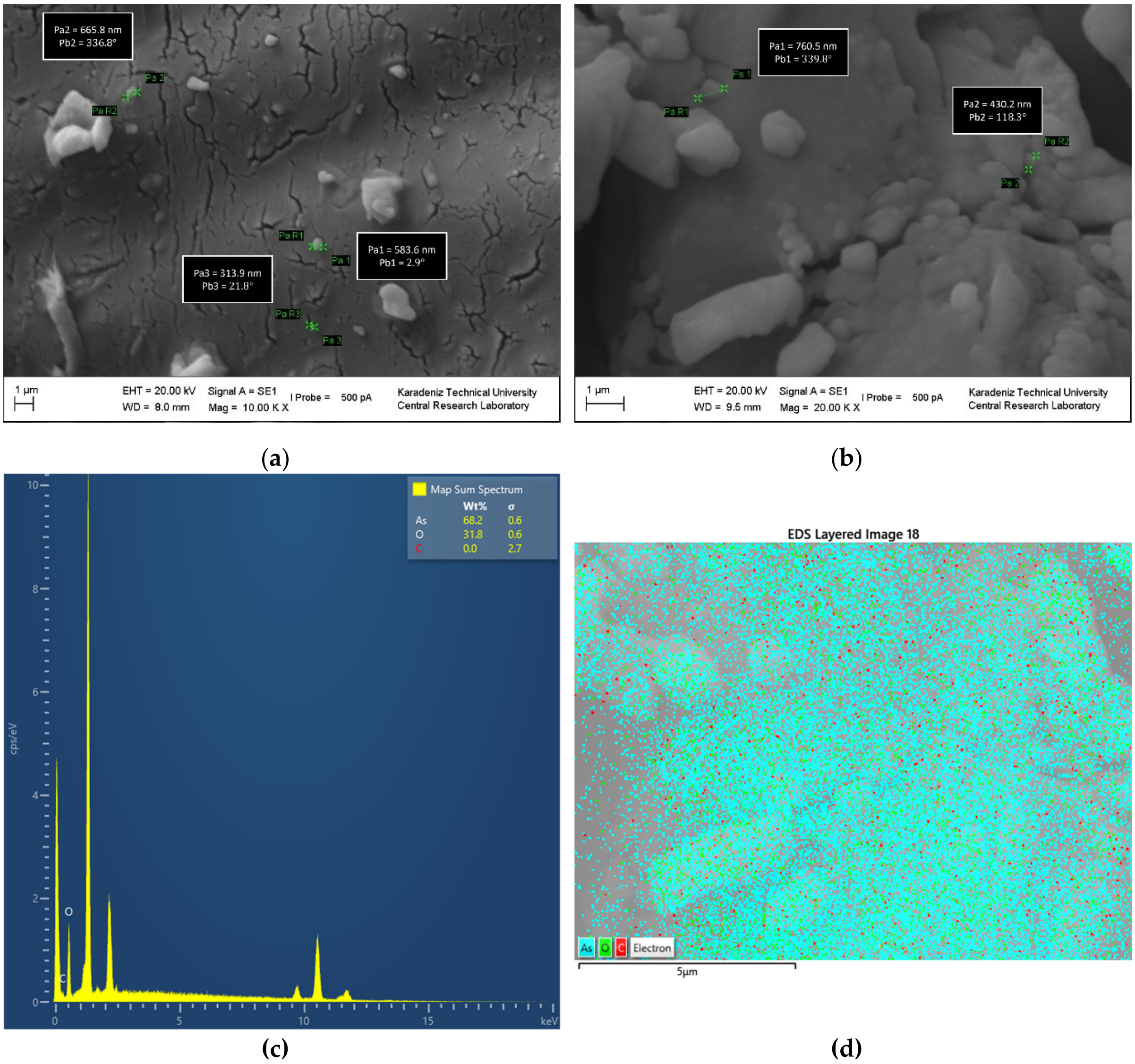

Figure S3. (a-b) SEM images **(c)** EDX (Map Sum Spectrum) graph and **(d)** EDS Layered Image of RP3A-1 nanocomposite.

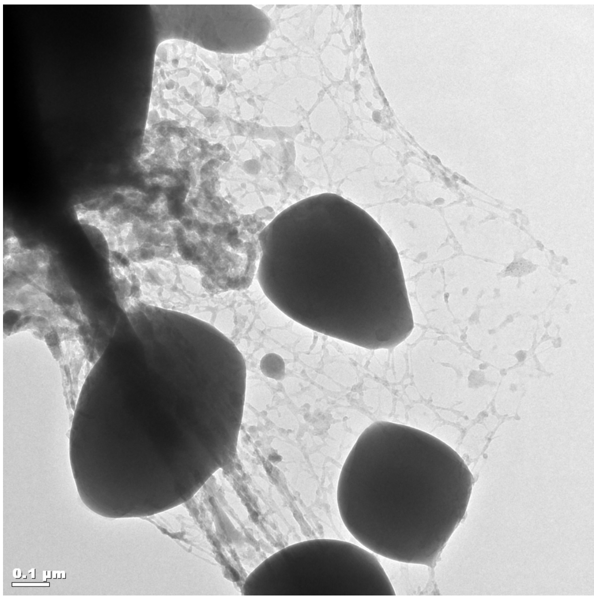
0.1 µm

(**a**)

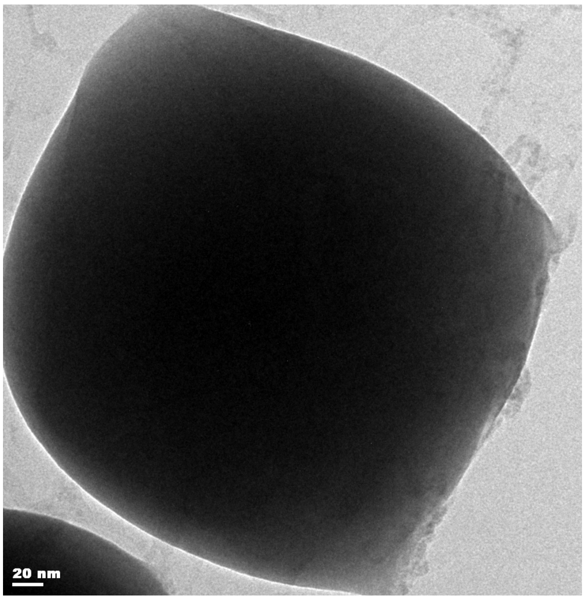
20 nm

(**b**)

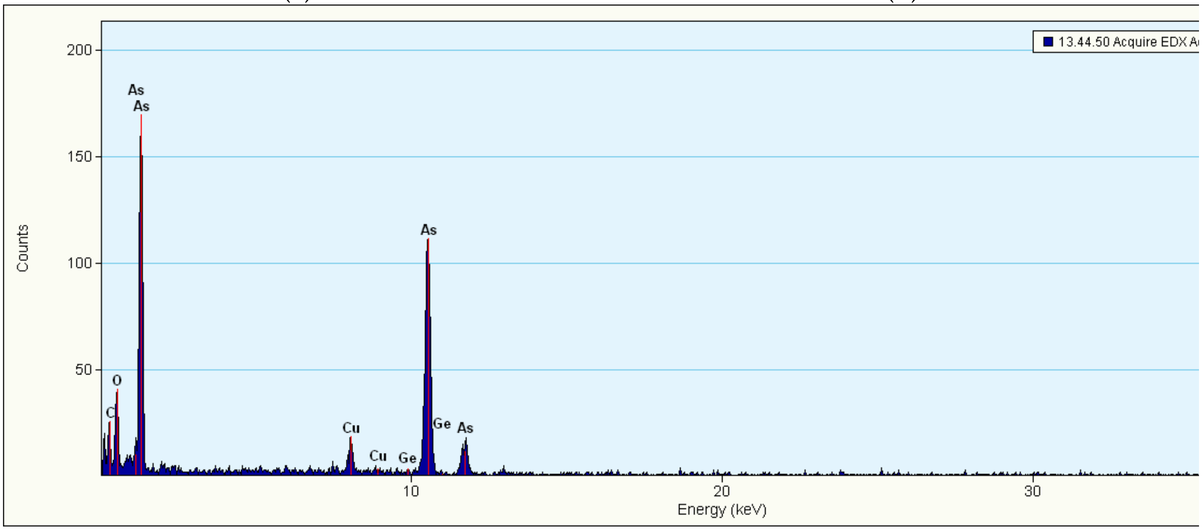
13.44.50 Acquire EDX A
200
150
100
50
Counts
As
As
O
C
Cu
Cu
Ge
As
Ge
As
10
20
30
Energy (keV)

(c)

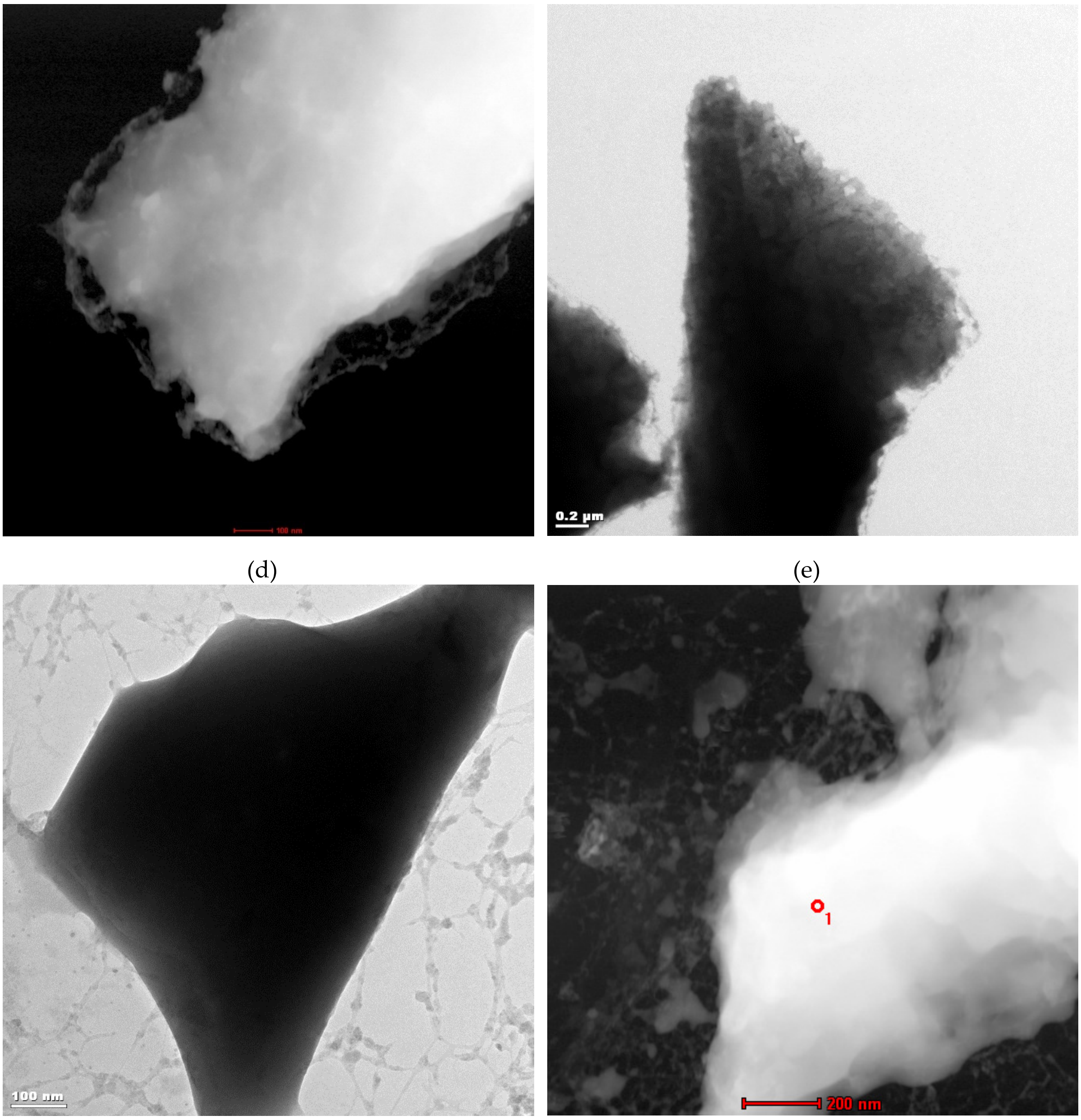

(d) (e)

(f) (g)

Figure S4 TEM pictures (a-b, d-g) and EDS Results (c) TEM images of of RP1A-2

RP1A-2 are presented in Figure SI4(a-g). Microscopic image in Figure SI4(a), RP1A-2 shows the fine particle size and crystal structure of the nanocomposite.of RP1A-2. EDX was performed to define the chemical composition of the nanocomposite. There are C, O, and As atoms in Figure SI4(c). C and O atoms indicate the presence of PS-PEG copolymer. As atoms indicate the presence of As_2O_3 NPs in the nanocomposite. The microscopic image in Figure SI4 (a-g) and Figure SI 3 represents the As_2O_3 nanoparticle embedded in the mesh-like structure of the PS-PEG copolymer.

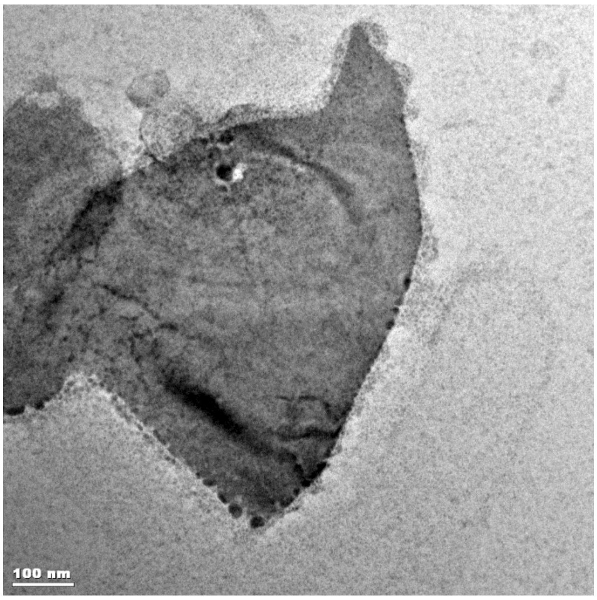

(**a**)

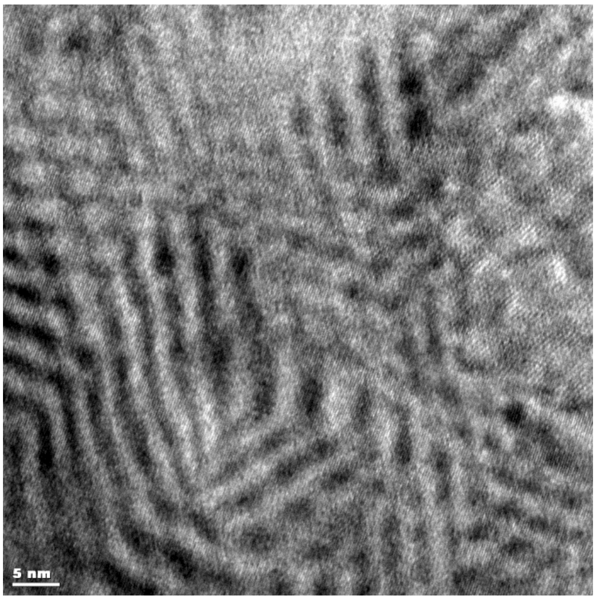

(**b**)

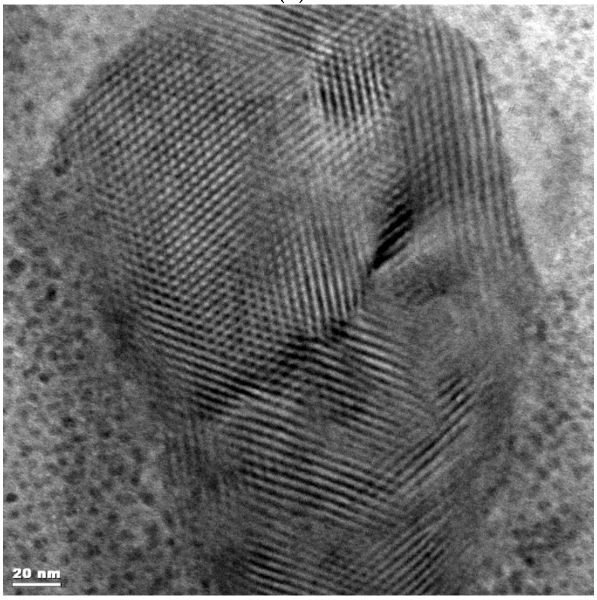

(c)

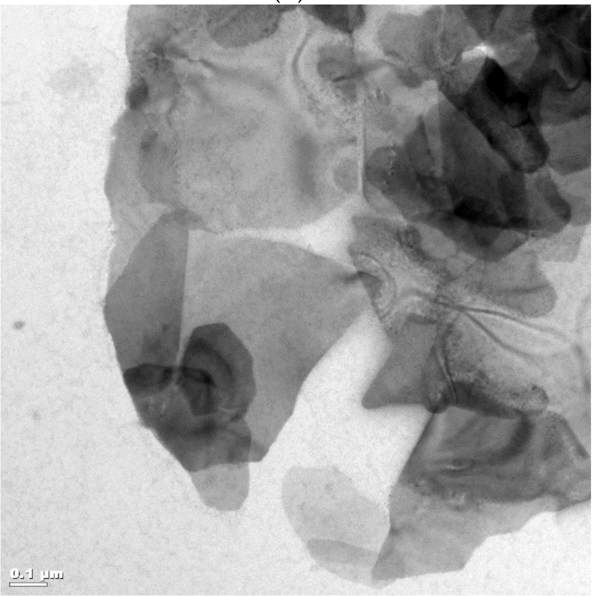

(d)

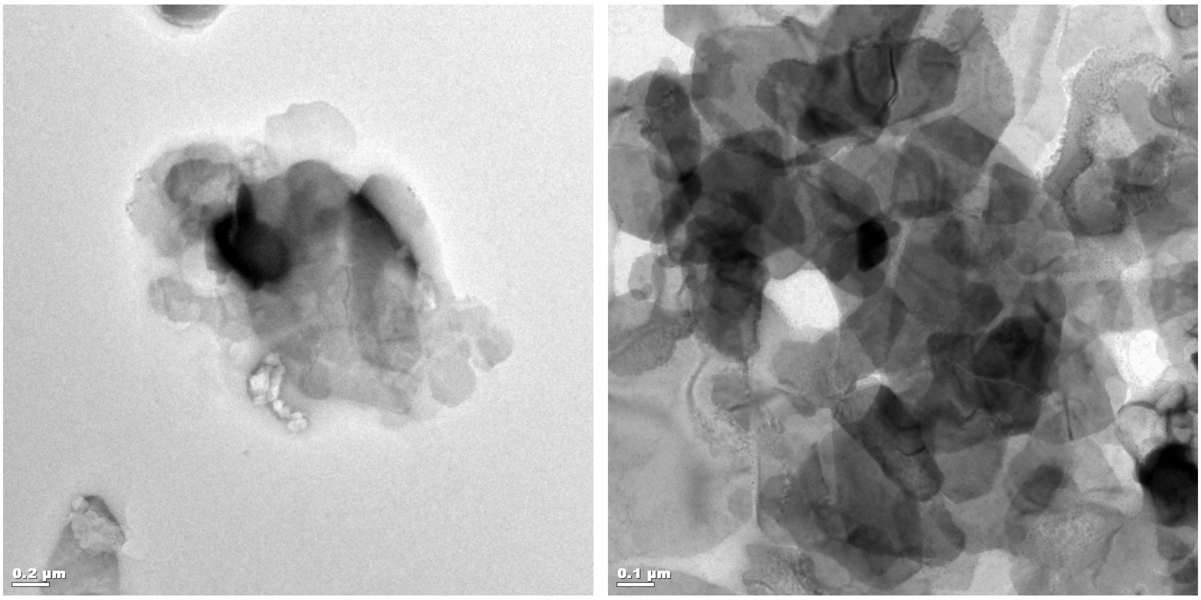

(e) **(f)**

Figure S5. (a-f TEM pictures of RP2A-2

Apart from the agglomerated small NPs, individual larger particles were observed without agglomeration (Figure S5 a, b, c, d). Particle distribution within the PS-PEG copolymer was characterized by TEM. TEM has been used as a successful method to study the distribution of NPs embedded in the copolymer. Figure S5 shows that although some particles are aggregated into microparticles, honeycombs, and zigzags, the particles are in the nano size range and are uniformly distributed throughout the copolymer.

Figures 6(a-f) present TEM views of the RP3A-2 nanocomposite. Figure S5 photographs shows that, the As_2O_3 nanoparticles are agglomerated close to spherical, as in the form of spherical microparticles. Figure 3a shows that As_2O_3 particles are triangular in shape. Figure 6(b) shows the small-big size distribution of As_2O_3.

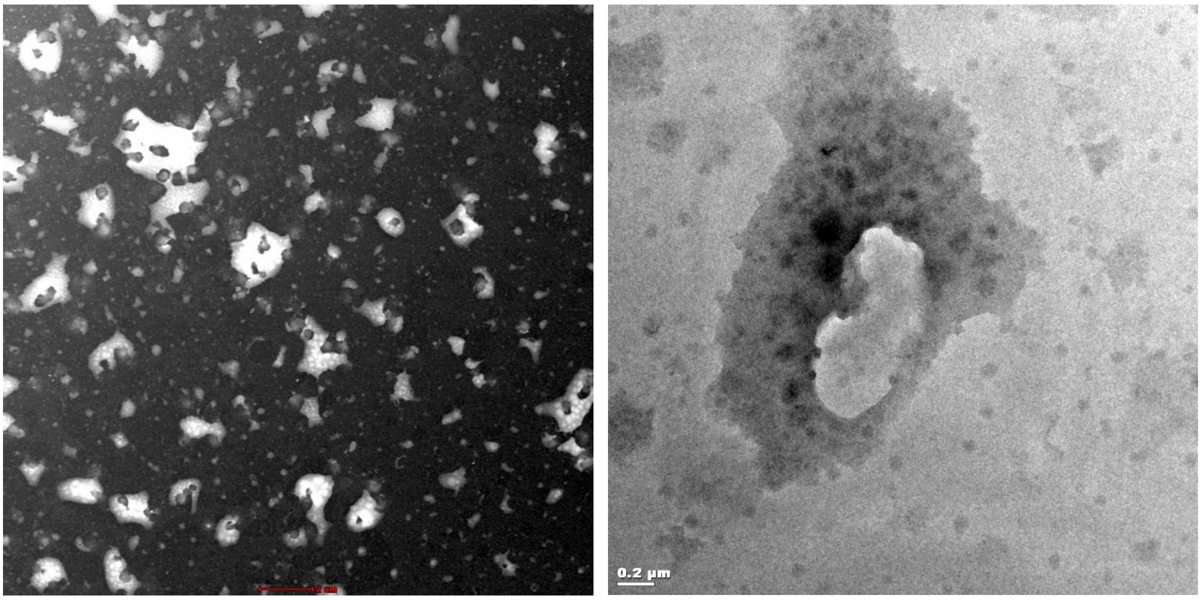

(**a**) (**b**)

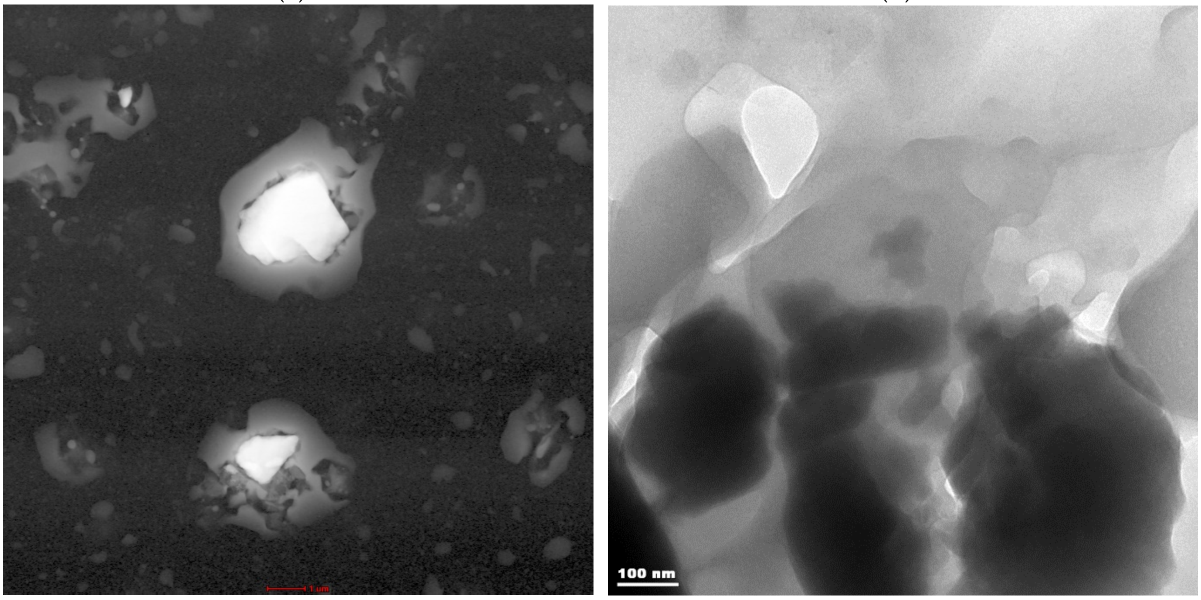

(c) **(d)**

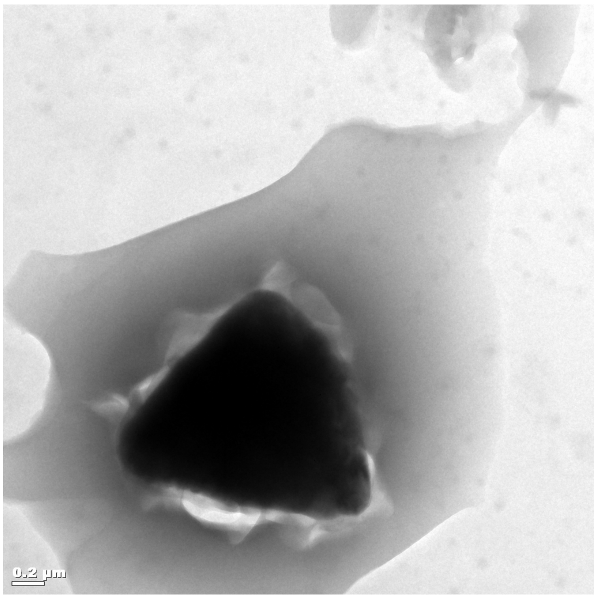

(e)

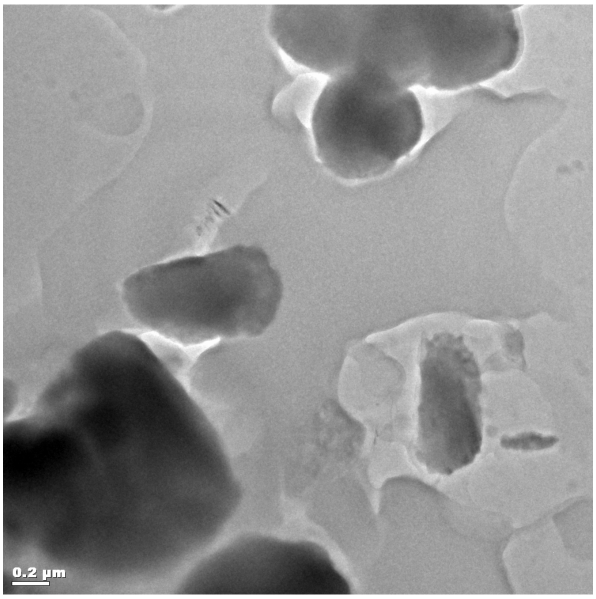

(f)

Figure S6. (a-f) TEM pictures of RP3A-2

Table S2. Experimental and XCOM μ_m Values of the nanocomposites.

Energy (KeV)	Exp.	XCOM	Exp.	XCOM	Exp.	XCOM	Exp.	XCOM	Exp.	XCOM
	**RPA1**		**RP1A-1**		**RP1A-2**		**RP1A-3**		**RP1A-4**	
121.8	0,235	0,267	0,296	0,256	0,170	0,264	0,269	0,439	0,163	0,283
344.3	0,198	0,188	0,086	0,113	0,064	0,101	0,078	0,147	0,082	0,121
778.9	0,137	0,133	0,073	0,077	0,073	0,067	0,076	0,096	0,074	0,082
964.1	0,098	0,120	0,060	0,069	0,068	0,060	0,083	0,086	0,059	0,074
1085.9	0,092	0,114	0,060	0,065	0,055	0,057	0,081	0,081	0,068	0,069
1112.1	0,080	0,112	0,065	0,064	0,052	0,056	0,087	0,080	0,064	0,068
1408.0	0,080	0,099	0,049	0,057	0,030	0,050	0,024	0,071	0,041	0,061
	**RPA2**		**RP2A-1**		**RP2A-2**		**RP2A-3**		**RP2A-4**	
121.8	0,283	0,250	0,183	0,241	0,342	0,289	0,237	0,335	0,143	0,241
344.3	0,194	0,176	0,091	0,106	0,065	0,110	0,062	0,112	0,055	0,103
778.9	0,141	0,125	0,088	0,072	0,072	0,073	0,060	0,073	0,049	0,070
964.1	0,111	0,113	0,066	0,065	0,046	0,066	0,069	0,066	0,057	0,063
1085.9	0,086	0,106	0,067	0,061	0,061	0,062	0,074	0,062	0,050	0,059
1112.1	0,068	0,105	0,057	0,060	0,060	0,061	0,055	0,061	0,057	0,058
1408.0	0,062	0,093	0,045	0,054	0,051	0,054	0,049	0,054	0,041	0,052
	**RPA3**		**RP3A-1**		**RP3A-2**		**RP3A-3**		**RP3A-4**	
121.8	0,177	0,183	0,149	0,215	0,215	0,319	0,336	0,297	0,225	0,319
344.3	0,156	0,129	0,067	0,095	0,086	0,121	0,088	0,099	0,088	0,136
778.9	0,071	0,091	0,085	0,064	0,085	0,081	0,077	0,065	0,088	0,092
964.1	0,077	0,083	0,067	0,058	0,056	0,073	0,066	0,058	0,079	0,083
1085.9	0,062	0,078	0,062	0,055	0,074	0,069	0,071	0,055	0,066	0,078
1112.1	0,057	0,077	0,071	0,054	0,069	0,068	0,074	0,054	0,078	0,077
1408.0	0,044	0,068	0,047	0,048	0,048	0,060	0,052	0,048	0,051	0,068
	**RPABN1**		**RP1ABN-1**		**RP1ABN-2**		**RP1ABN-3**			
121.8	0,293	0,173	0,243	0,201	0,244	0,278	0,220	0,260		
344.3	0,070	0,122	0,081	0,076	0,097	0,092	0,108	0,105		
778.9	0,072	0,086	0,069	0,050	0,063	0,060	0,073	0,070		
964.1	0,064	0,078	0,054	0,045	0,052	0,054	0,071	0,063		
1085.9	0,060	0,073	0,053	0,043	0,051	0,051	0,070	0,060		
1112.1	0,047	0,072	0,045	0,042	0,053	0,050	0,069	0,059		
1408.0	0,030	0,064	0,037	0,037	0,037	0,045	0,037	0,052		
	**RPABN2**		**RP2ABN-1**		**RP2ABN-2**		**RP2ABN-3**			
121.8	0,151	0,145	0,271	0,262	0,337	0,257	0,223	0,284		
344.3	0,128	0,103	0,085	0,099	0,095	0,085	0,064	0,115		
778.9	0,078	0,073	0,066	0,066	0,059	0,056	0,059	0,077		
964.1	0,069	0,066	0,065	0,059	0,046	0,050	0,052	0,069		
1085.9	0,068	0,062	0,060	0,056	0,036	0,047	0,068	0,065		

1112.1	0,066	0,061	0,052	0,055	0,035	0,047	0,078	0,064
1408.0	0,052	0,054	0,036	0,049	0,026	0,041	0,036	0,057
	**RPABN3**		**RP3ABN-1**		**RP3ABN-2**		**RP3ABN-3**	
121.8	0,142	0,153	0,423	0,550	0,383	0,358	0,187	0,253
344.3	0,094	0,108	0,272	0,207	0,106	0,119	0,115	0,102
778.9	0,080	0,076	0,177	0,138	0,097	0,078	0,075	0,068
964.1	0,061	0,069	0,095	0,124	0,053	0,070	0,012	0,062
1085.9	0,067	0,065	0,112	0,117	0,086	0,066	0,064	0,058
1112.1	0,061	0,064	0,125	0,115	0,073	0,065	0,062	0,057
1408.0	0,052	0,057	0,042	0,102	0,049	0,057	0,014	0,051

Table S3. Experimental and XCOM μ_L Values of the Nanocomposites.

Energy (KeV)	Exp.	XCOM	Exp.	XCOM	Exp.	XCOM	Exp.	XCOM	Exp.	XCOM
	**RPA1**		**RP1A-1**		**RP1A-2**		**RP1A-3**		**RP1A-4**	
121.8	0,241±0,05	0,274	0,499±0,18	0,432	0,346±0,04	0,539	0,899±0,79	1,467	0,276±0,18	0,478
344.3	0,203±0,04	0,193	0,146±0,15	0,190	0,131±0,13	0,205	0,262±0,42	0,489	0,138±0,14	0,204
778.9	0,141±0,18	0,137	0,123±0,11	0,129	0,149±0,15	0,137	0,253±0,41	0,320	0,125±0,08	0,138
964.1	0,100±0,13	0,123	0,100±0,19	0,116	0,139±0,23	0,123	0,278±0,36	0,287	0,100±0,08	0,125
1085.9	0,094±0,24	0,116	0,102±0,24	0,109	0,112±0,20	0,116	0,272±0,48	0,270	0,115±0,10	0,117
1112.1	0,082±0,13	0,115	0,110±0,14	0,108	0,106±0,21	0,114	0,290±0,08	0,267	0,108±0,17	0,116
1408.0	0,082±0,07	0,102	0,083±0,21	0,096	0,060±0,19	0,101	0,079±0,33	0,237	0,069±0,14	0,103
	**RPA2**		**RP2A-1**		**RP2A-2**		**RP2A-3**		**RP2A-4**	
121.8	0,282±0,16	0,249	0,302±0,06	0,397	0,685±0,10	0,579	0,619±0,06	0,877	0,244±0,08	0,411
344.3	0,193±0,09	0,176	0,150±0,18	0,175	0,129±0,26	0,220	0,162±0,43	0,292	0,093±0,11	0,176
778.9	0,141±0,05	0,124	0,145±0,08	0,119	0,145±0,15	0,147	0,158±0,26	0,191	0,083±0,12	0,119
964.1	0,111±0,13	0,112	0,108±0,24	0,107	0,093±0,07	0,132	0,180±0,17	0,172	0,097±0,09	0,107
1085.9	0,085±0,19	0,106	0,110±0,22	0,101	0,122±0,04	0,124	0,193±0,11	0,162	0,086±0,09	0,101
1112.1	0,068±0,08	0,105	0,093±0,13	0,100	0,119±0,25	0,123	0,144±0,21	0,160	0,097±0,12	0,100
1408.0	0,062±0,15	0,093	0,074±0,13	0,088	0,102±0,19	0,109	0,128±0,03	0,141	0,070±0,19	0,088
	**RPA3**		**RP3A-1**		**RP3A-2**		**RP3A-3**		**RP3A-4**	
121.8	0,195±0,13	0,202	0,258±0,11	0,374	0,473±0,16	0,700	0,815±0,23	0,720	0,401±0,05	0,569
344.3	0,172±0,06	0,142	0,116±0,04	0,165	0,188±0,22	0,266	0,212±0,27	0,240	0,157±0,13	0,243
778.9	0,078±0,20	0,100	0,148±0,13	0,112	0,186±0,22	0,177	0,187±0,16	0,157	0,156±0,16	0,164
964.1	0,085±0,06	0,091	0,116±0,12	0,101	0,122±0,20	0,160	0,159±0,10	0,141	0,141±0,16	0,148
1085.9	0,068±0,09	0,086	0,109±0,14	0,095	0,163±0,16	0,150	0,173±0,09	0,133	0,118±0,28	0,139
1112.1	0,062±0,08	0,085	0,124±0,07	0,094	0,151±0,24	0,148	0,180±0,30	0,131	0,139±0,05	0,138
1408.0	0,049±0,11	0,075	0,082±0,02	0,083	0,105±0,13	0,132	0,126±0,20	0,116	0,092±0,07	0,122
	**RPABN1**		**RP1ABN-1**		**RP1ABN-2**		**RP1ABN-3**			
121.8	0,362±0,13	0,213	0,513±0,15	0,423	0,593±0,34	0,675	0,433±0,14	0,511		
344.3	0,087±0,11	0,150	0,170±0,17	0,159	0,236±0,16	0,224	0,214±0,22	0,206		
778.9	0,089±0,14	0,106	0,146±0,14	0,106	0,152±0,23	0,147	0,143±0,13	0,138		
964.1	0,079±0,14	0,096	0,114±0,03	0,095	0,126±0,34	0,132	0,140±0,15	0,125		
1085.9	0,074±0,09	0,091	0,113±0,13	0,090	0,125±0,20	0,124	0,137±0,16	0,117		
1112.1	0,058±0,18	0,089	0,096±0,05	0,089	0,130±0,24	0,122	0,135±0,28	0,116		
1408.0	0,037±0,11	0,079	0,079±0,15	0,079	0,090±0,17	0,109	0,073±0,04	0,103		
	**RPABN2**		**RP2ABN-1**		**RP2ABN-2**		**RP2ABN-3**			
121.8	0,260±0,16	0,310	0,613±0,15	0,592	0,717±0,15	0,546	0,395±0,15	0,503		
344.3	0,220±0,07	0,219	0,192±0,18	0,223	0,201±0,29	0,181	0,113±0,12	0,203		
778.9	0,134±0,19	0,155	0,149±0,08	0,149	0,125±0,08	0,119	0,104±0,19	0,136		
964.1	0,119±0,08	0,140	0,147±0,34	0,134	0,098±0,30	0,106	0,092±0,08	0,123		

1085.9	0,118±0,17	0,132	0,136±0,14	0,126	0,077±0,24	0,100	0,121±0,15	0,115
1112.1	0,114±0,17	0,130	0,117±0,16	0,124	0,075±0,08	0,099	0,139±0,01	0,114
1408.0	0,089±0,12	0,115	0,083±0,20	0,110	0,055±0,24	0,088	0,063±0,25	0,101
	**RPABN3**		**RP3ABN-1**		**RP3ABN-2**		**RP3ABN-3**	
121.8	0,186±0,15	0,200	0,761±0,31	0,987	0,449±0,10	0,419	0,410±0,21	0,554
344.3	0,123±0,07	0,141	0,489±0,40	0,372	0,125±0,10	0,139	0,253±0,06	0,223
778.9	0,104±0,21	0,100	0,317±0,39	0,248	0,114±0,06	0,091	0,164±0,25	0,150
964.1	0,080±0,14	0,090	0,171±0,21	0,223	0,063±0,19	0,082	0,148±0,13	0,135
1085.9	0,087±0,14	0,085	0,202±0,09	0,210	0,100±0,12	0,077	0,140±0,17	0,127
1112.1	0,080±0,05	0,084	0,225±0,21	0,207	0,086±0,14	0,076	0,136±0,15	0,126
1408.0	0,069±0,17	0,074	0,075±0,25	0,184	0,057±0,06	0,067	0,086±0,21	0,111

Table S4. RPE(%), HVL, TVL, MFP Values of the Nanocomposites

	HVL (cm)	*TVL (cm)*	*MFP (cm)*	*RPE(%)*	*HVL (cm)*	*TVL (cm)*	*MFP (cm)*	*RPE(%)*
	**RPA1**				**RP1A-1**			
121.8	2,88	9,57	4,16	10,22	1,39	4,61	2,00	19,59
344.3	3,42	11,36	4,94	8,68	4,76	15,80	6,86	6,17
778.9	4,93	16,38	7,11	6,10	5,64	18,74	8,14	5,23
964.1	6,93	23,01	9,99	4,38	6,91	22,96	9,97	4,29
1085.9	7,38	24,52	10,65	4,12	6,82	22,64	9,83	4,35
1112.1	8,43	28,01	12,16	3,62	6,30	20,94	9,09	4,69
1408.0	8,46	28,10	12,20	3,60	8,36	27,78	12,06	3,56
	**RP1A-2**				**RP1A-3**			
121.8	2,00	6,66	2,89	12,95	0,77	2,56	1,11	13,79
344.3	5,29	17,56	7,63	5,12	2,65	8,79	3,82	4,23
778.9	4,64	15,40	6,69	5,82	2,73	9,08	3,95	4,10
964.1	4,99	16,56	7,19	5,42	2,50	8,30	3,60	4,48
1085.9	6,16	20,47	8,89	4,41	2,55	8,47	3,68	4,39
1112.1	6,56	21,79	9,46	4,15	2,39	7,94	3,45	4,67
1408.0	11,50	38,19	16,59	2,39	8,74	29,04	12,61	1,30
	**RP1A-4**				**RPA2**			
121.8	2,51	8,35	3,63	13,17	2,46	8,16	3,54	12,96
344.3	5,02	16,66	7,24	6,83	3,59	11,93	5,18	9,06
778.9	5,56	18,47	8,02	6,18	4,93	16,37	7,11	6,68
964.1	6,97	23,14	10,05	4,97	6,24	20,74	9,01	5,32
1085.9	6,03	20,03	8,70	5,72	8,12	26,99	11,72	4,11
1112.1	6,43	21,36	9,28	5,37	10,21	33,91	14,73	3,29
1408.0	10,03	33,31	14,47	3,48	11,27	37,42	16,25	2,98
	**RP2A-1**				**RP2A-2**			

121.8	2,30	7,63	3,31	13,41	1,01	3,36	1,46	22,55
344.3	4,63	15,38	6,68	6,89	5,36	17,81	7,74	4,71
778.9	4,78	15,86	6,89	6,69	4,78	15,87	6,89	5,27
964.1	6,39	21,23	9,22	5,04	7,48	24,84	10,79	3,40
1085.9	6,29	20,89	9,07	5,12	5,68	18,87	8,20	4,45
1112.1	7,43	24,67	10,71	4,35	5,81	19,29	8,38	4,35
1408.0	9,39	31,18	13,54	3,46	6,78	22,51	9,78	3,74
	**RP2A-3**				**RP2A-4**			
121.8	1,12	3,72	1,61	15,77	2,84	9,44	4,10	13,47
344.3	4,27	14,17	6,16	4,40	7,43	24,67	10,71	5,38
778.9	4,39	14,59	6,34	4,28	8,35	27,73	12,04	4,80
964.1	3,85	12,80	5,56	4,86	7,13	23,70	10,29	5,60
1085.9	3,60	11,95	5,19	5,20	8,03	26,66	11,58	4,99
1112.1	4,82	16,00	6,95	3,91	7,15	23,76	10,32	5,58
1408.0	5,42	18,02	7,82	3,48	9,84	32,67	14,19	4,09
	**RPA3**				**RP3A-1**			
121.8	3,56	11,84	5,14	11,24	2,68	8,91	3,87	12,33
344.3	4,03	13,39	5,82	10,00	5,99	19,89	8,64	5,72
778.9	8,94	29,68	12,89	4,64	4,67	15,51	6,74	7,28
964.1	8,15	27,06	11,75	5,08	5,95	19,78	8,59	5,75
1085.9	10,18	33,81	14,68	4,09	6,39	21,22	9,22	5,37
1112.1	11,10	36,89	16,02	3,75	5,60	18,62	8,09	6,10
1408.0	14,22	47,25	20,52	2,94	8,50	28,24	12,26	4,07
	**RP3A-2**				**RP3A-3**			
121.8	1,47	4,87	2,12	13,63	0,85	2,82	1,23	24,15
344.3	3,69	12,25	5,32	5,66	3,26	10,84	4,71	6,95
778.9	3,72	12,37	5,37	5,61	3,70	12,28	5,33	6,16
964.1	5,69	18,89	8,20	3,71	4,35	14,44	6,27	5,26
1085.9	4,26	14,14	6,14	4,92	4,01	13,31	5,78	5,69
1112.1	4,58	15,22	6,61	4,58	3,86	12,81	5,56	5,91
1408.0	6,58	21,86	9,49	3,21	5,49	18,25	7,93	4,19
	**RP3A-4**				**RPABN1**			
121.8	1,73	5,74	2,49	15,94	1,92	6,37	2,77	17,29
344.3	4,41	14,64	6,36	6,58	7,98	26,50	11,51	4,46
778.9	4,43	14,72	6,39	6,55	7,81	25,94	11,26	4,55
964.1	4,92	16,34	7,10	5,92	8,74	29,02	12,60	4,08
1085.9	5,87	19,48	8,46	4,99	9,30	30,91	13,42	3,84
1112.1	4,99	16,57	7,20	5,84	11,87	39,42	17,12	3,02
1408.0	7,58	25,16	10,93	3,88	18,50	61,45	26,69	1,95
	**RP1ABN-1**				**RP1ABN-2**			
121.8	1,35	4,49	1,95	22,23	1,17	3,88	1,69	18,89
344.3	4,07	13,52	5,87	8,01	2,93	9,74	4,23	8,01
778.9	4,76	15,82	6,87	6,88	4,56	15,13	6,57	5,23

964.1	6,07	20,16	8,75	5,44	5,51	18,31	7,95	4,34
1085.9	6,15	20,43	8,87	5,37	5,56	18,48	8,03	4,30
1112.1	7,24	24,06	10,45	4,58	5,33	17,71	7,69	4,49
1408.0	8,80	29,22	12,69	3,79	7,69	25,54	11,09	3,13
	**RP1ABN-3**				**RPABN2**			
121.8	1,60	5,32	2,31	17,16	2,66	8,84	3,84	10,89
344.3	3,24	10,78	4,68	8,87	3,15	10,46	4,54	9,29
778.9	4,84	16,06	6,98	6,04	5,17	17,18	7,46	5,77
964.1	4,96	16,49	7,16	5,90	5,85	19,43	8,44	5,11
1085.9	5,04	16,76	7,28	5,80	5,87	19,50	8,47	5,10
1112.1	5,13	17,05	7,40	5,71	6,07	20,16	8,76	4,93
1408.0	9,47	31,47	13,67	3,13	7,80	25,91	11,25	3,86
	**RP2ABN-1**				**RP2ABN-2**			
121.8	1,13	3,76	1,63	19,71	0,97	3,21	1,40	27,20
344.3	3,60	11,97	5,20	6,66	3,45	11,46	4,98	8,51
778.9	4,65	15,43	6,70	5,20	5,53	18,38	7,98	5,40
964.1	4,73	15,70	6,82	5,11	7,06	23,45	10,19	4,26
1085.9	5,10	16,93	7,35	4,75	9,05	30,07	13,06	3,34
1112.1	5,91	19,62	8,52	4,11	9,27	30,80	13,38	3,26
1408.0	8,40	27,91	12,12	2,91	12,60	41,86	18,18	2,41
	**RP2ABN-3**				**RPABN3**			
121.8	1,76	5,83	2,53	16,05	3,72	12,36	5,37	9,89
344.3	6,14	20,38	8,85	4,88	5,62	18,66	8,10	6,67
778.9	6,67	22,15	9,62	4,50	6,65	22,09	9,59	5,66
964.1	7,53	25,02	10,87	3,99	8,69	28,86	12,54	4,36
1085.9	5,73	19,03	8,27	5,22	7,96	26,43	11,48	4,75
1112.1	4,99	16,57	7,20	5,97	8,70	28,91	12,55	4,36
1408.0	10,99	36,51	15,86	2,75	10,11	33,59	14,59	3,76
	**RP3ABN-1**				**RP3ABN-2**			
121.8	0,91	3,02	1,31	14,71	1,54	5,12	2,23	22,21
344.3	1,42	4,71	2,05	9,71	5,56	18,48	8,02	6,73
778.9	2,18	7,26	3,15	6,42	6,07	20,17	8,76	6,18
964.1	4,05	13,46	5,85	3,51	11,08	36,80	15,98	3,44
1085.9	3,43	11,40	4,95	4,13	6,90	22,93	9,96	5,46
1112.1	3,08	10,22	4,44	4,60	8,04	26,72	11,60	4,70
1408.0	9,27	30,79	13,37	1,55	12,12	40,27	17,49	3,15
	**RP3ABN-3**							
121.8	1,69	5,62	2,44	15,29				
344.3	2,74	9,10	3,95	9,74				
778.9	4,23	14,04	6,10	6,42				
964.1	4,68	15,56	6,76	1,05				
1085.9	4,95	16,45	7,14	5,52				
1112.1	5,10	16,93	7,35	5,37				

1408.0	8,06	26,77	11,63	1,20	

Table S5. XRD value

RP1A-1		RP2A-1		RP3A-1		RP1ABN-1		RP2ABN-1		RP3ABN-1	
2θ (⁰)	hkl	2θ (⁰)	hkl	2θ (⁰)	hkl	2θ (⁰)	hkl	2θ (⁰)	hkl	2θ (⁰)	hkl
13.96	(002)	13.96	(002)	13.96	(002)	13.96	(002)	21.29	(021)	13.96	(002)
28.02	(122)	28.02	(122)	28.02	(021)	28.02	(221)	25.54	(002)	27.90	(201)
32.39	(112)	32.39	(112)	32.39	(210)	32.39	(112)	26.72	(002)	32.39	(102)
35.46	(200)	35.46	(200)	35.46	(002)	35.46	(200)	41.89	(202)	35.46	(102)
46.21	(203)	46.21	(203)	46.21	(203)	46.21	(203)			46.21	(203)

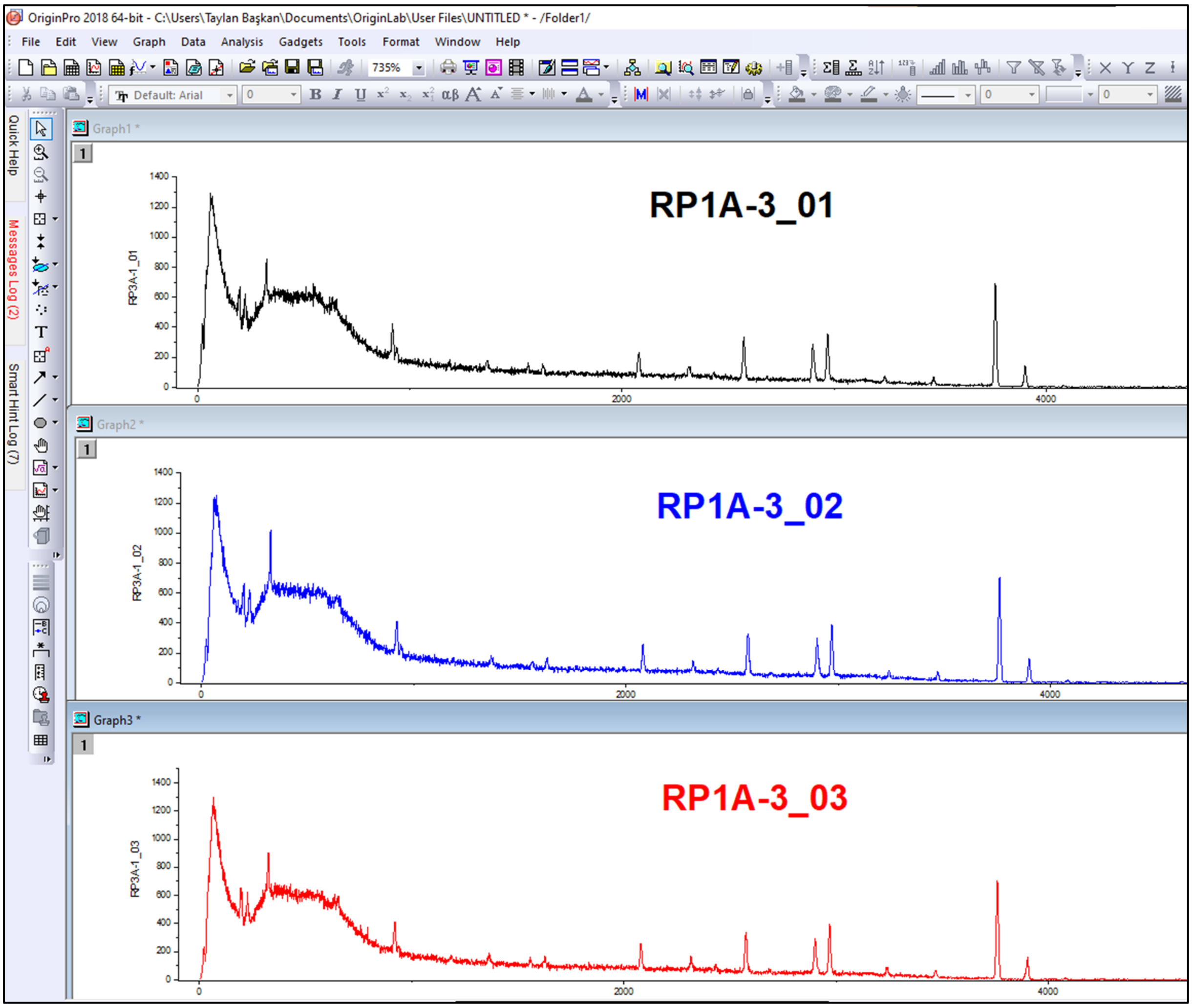

Figure S7. Origin graphs plotted for three separate measurements of sample RP1A-3

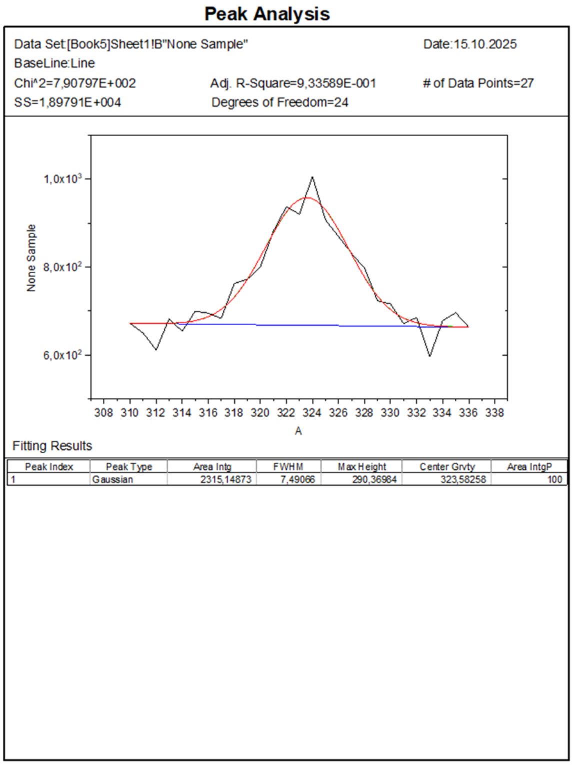

(a)

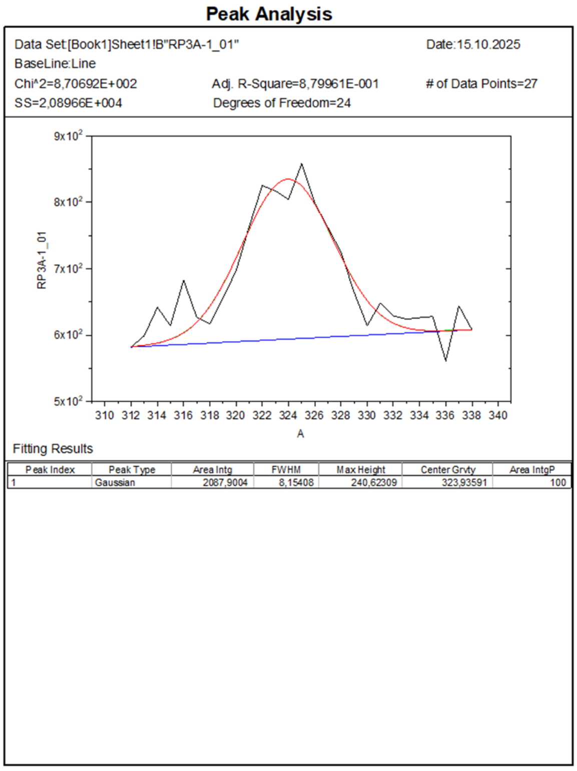

(b)

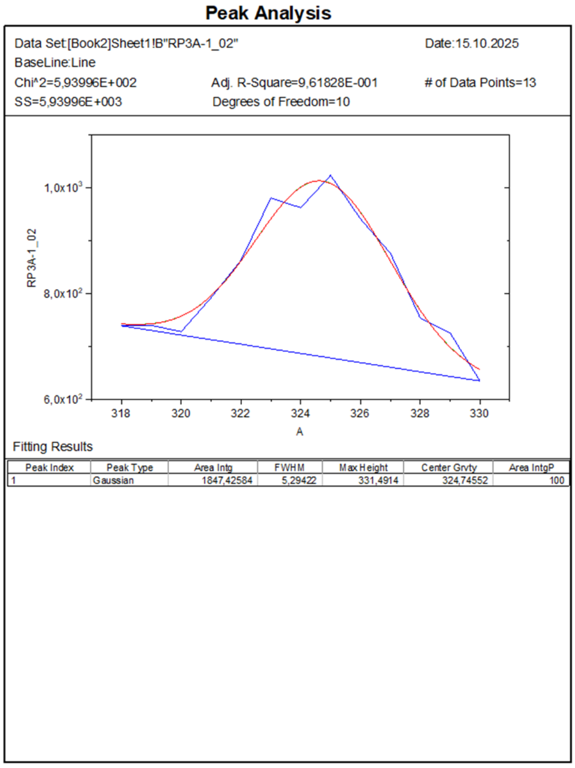

(c)

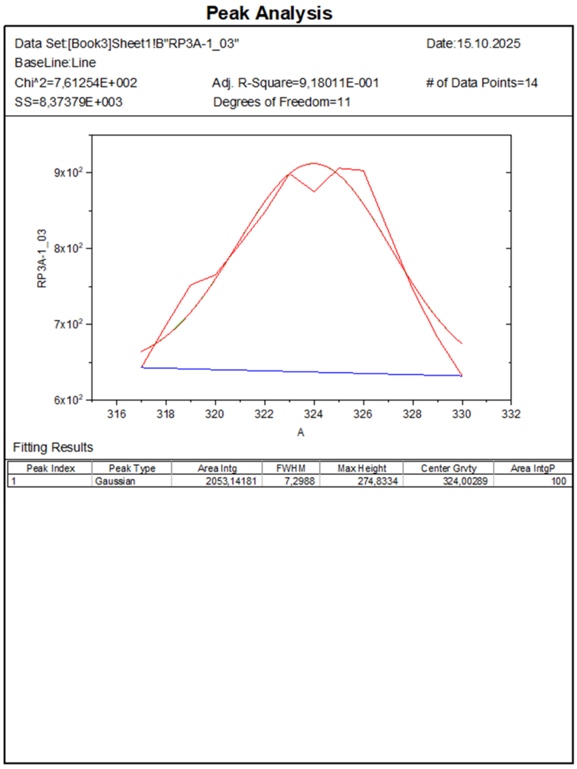

(d)

Figure S8. RP3A-1 sample peak analysis for 121.78 KeV (a) Peak area for measurement without sample (b) First RP3A-1 measurement (c) Second First RP3A-1 measurement (d) Third RP3A-1 measurement

Peak Analysis

Data Set:[Book5]Sheet1!B"None Sample" Date:15.10.2025
BaseLine:Line
Chi^2=2,36182E+002 Adj. R-Square=9,38701E-001 # of Data Points=52
SS=1,08643E+004 Degrees of Freedom=46

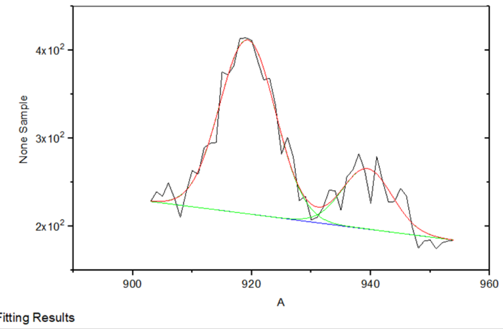

Fitting Results

Peak Index	Peak Type	Area Intg	FWHM	Max Height	Center Grvty	Area IntgP
1	Gaussian	2325,70338	11,06091	197,58091	919,29989	76,40977
2	Gaussian	718,02181	9,82626	68,66265	939,4127	23,59023

(a)

Peak Analysis

Data Set:[Book1]Sheet1!B"RP3A-1_01" Date:15.10.2025
BaseLine:Line
Chi^2=2,36839E+002 Adj. R-Square=9,35366E-001 # of Data Points=48
SS=9,94724E+003 Degrees of Freedom=42

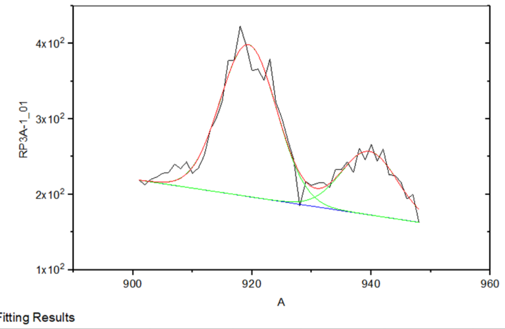

Fitting Results

Peak Index	Peak Type	Area Intg	FWHM	Max Height	Center Grvty	Area IntgP
1	Gaussian	2378,73491	11,09745	201,37796	919,37012	71,45774
2	Gaussian	950,13449	11,04217	84,03738	939,68218	28,54226

(b)

Peak Analysis

Data Set:[Book2]Sheet1!B"RP3A-1_02" Date:15.10.2025
BaseLine:Line
Chi^2=2,58369E+002 Adj. R-Square=9,34820E-001 # of Data Points=60
SS=1,39520E+004 Degrees of Freedom=54

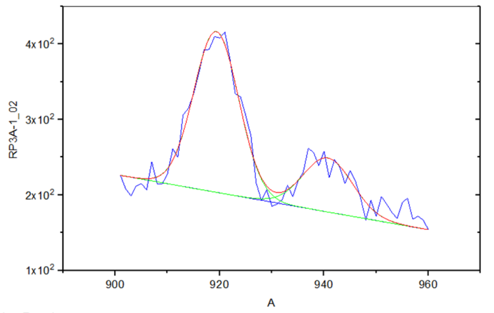

Fitting Results

Peak Index	Peak Type	Area Intg	FWHM	Max Height	Center Grvty	Area IntgP
1	Gaussian	2288,71531	10,10355	212,80895	919,34576	72,97833
2	Gaussian	847,44209	11,13538	71,49615	940,72052	27,02167

(c)

Peak Analysis

Data Set:[Book3]Sheet1!B"RP3A-1_03" Date:15.10.2025
BaseLine:Line
Chi^2=2,83599E+002 Adj. R-Square=9,16637E-001 # of Data Points=38
SS=9,07518E+003 Degrees of Freedom=32

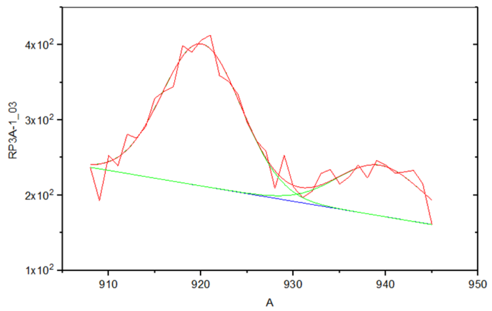

Fitting Results

Peak Index	Peak Type	Area Intg	FWHM	Max Height	Center Grvty	Area IntgP
1	Gaussian	2014,0112	10,04047	188,88758	920,05089	74,33208
2	Gaussian	695,46654	10,95829	67,27771	939,38307	25,66792

(d)

Figure S9. RP3A-1 sample peak analysis for 344,28 KeV (a) Peak area for measurement without sample (b) First RP3A-1 measurement (c) Second First RP3A-1 measurement (d) Third RP3A-1 measurement

Peak Analysis

Data Set:[Book5]Sheet1!B"None Sample" Date:15.10.2025
BaseLine:Line
Chi^2=1,12293E+002 Adj. R-Square=9,67438E-001 # of Data Points=1810
SS=2,01566E+005 Degrees of Freedom=1795

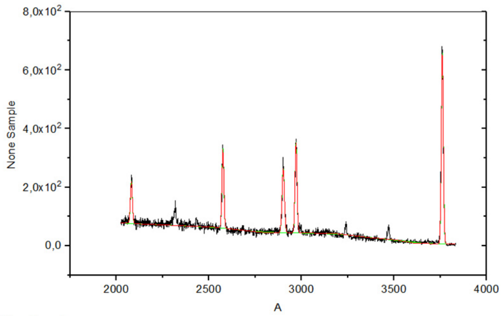

Fitting Results

Peak Index	Peak Type	Area Intg	FWHM	Max Height	Center Grvty	Area IntgP
1	Gaussian	1915,9738	12,37529	145,44608	2080,43273	8,72031
2	Gaussian	3542,88706	12,26611	271,34289	2574,75442	16,125
3	Gaussian	3506,43896	14,95764	220,22731	2900,82674	15,95911
4	Gaussian	4221,73398	12,77063	310,56061	2969,77663	19,21469
5	Gaussian	8784,35532	12,6377	652,99457	3760,22156	39,98088

(a)

Peak Analysis

Data Set:[Book1]Sheet1!B"RP3A-1_01" Date:15.10.2025
BaseLine:Line
Chi^2=1,18698E+002 Adj. R-Square=9,64515E-001 # of Data Points=1853
SS=2,18168E+005 Degrees of Freedom=1838

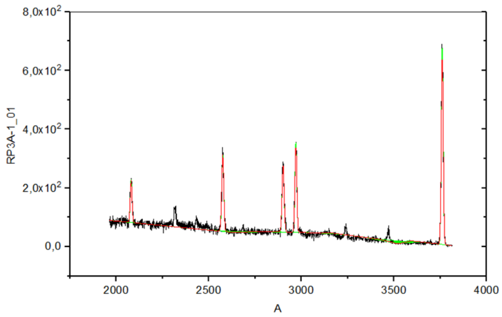

Fitting Results

Peak Index	Peak Type	Area Intg	FWHM	Max Height	Center Grvty	Area IntgP
1	Gaussian	1763,73078	11,3903	145,46719	2079,84448	8,39213
2	Gaussian	3599,54575	12,92989	261,52945	2574,55229	17,12725
3	Gaussian	3181,53165	13,19597	226,49715	2900,47544	15,13827
4	Gaussian	3905,47091	11,97726	306,32605	2969,65514	18,58289
5	Gaussian	8566,20836	12,01162	669,96929	3760,13724	40,75947

(b)

Peak Analysis

Data Set:[Book2]Sheet1!B"RP3A-1_02" Date:15.10.2025
BaseLine:Line
Chi^2=1,36178E+002 Adj. R-Square=9,62317E-001 # of Data Points=1811
SS=2,44576E+005 Degrees of Freedom=1796

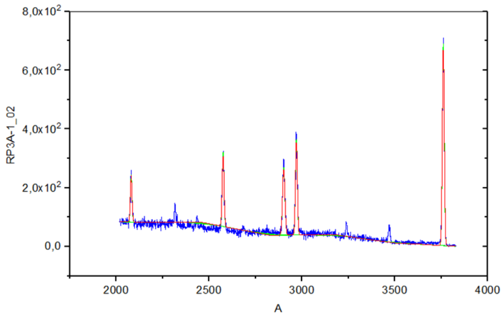

Fitting Results

Peak Index	Peak Type	Area Intg	FWHM	Max Height	Center Grvty	Area IntgP
1	Gaussian	1730,93412	10,19599	159,4846	2080,27971	8,04966
2	Gaussian	3118,49856	11,56851	253,24208	2574,91189	14,50248
3	Gaussian	3689,23092	15,05158	230,26168	2901,10664	17,15666
4	Gaussian	4213,01935	12,15403	325,64247	2969,70107	19,59252
5	Gaussian	8751,51992	11,98004	686,26717	3760,37814	40,69868

(c)

Peak Analysis

Data Set:[Book3]Sheet1!B"RP3A-1_03" Date:15.10.2025
BaseLine:Line
Chi^2=1,52753E+002 Adj. R-Square=9,57636E-001 # of Data Points=1767
SS=2,67623E+005 Degrees of Freedom=1752

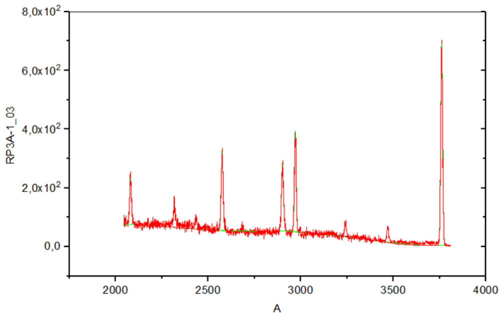

Fitting Results

Peak Index	Peak Type	Area Intg	FWHM	Max Height	Center Grvty	Area IntgP
1	Gaussian	2017,66084	12,07637	156,95666	2080,36697	9,47778
2	Gaussian	3434,96182	11,97241	269,53077	2574,6995	16,13542
3	Gaussian	3187,01025	13,20104	226,80003	2900,58421	14,97069
4	Gaussian	3954,77359	10,87365	341,67569	2969,5819	18,57719
5	Gaussian	8693,92351	11,96225	682,76412	3760,14595	40,83892

(d)

Figure S10. RP3A-1 sample peak analysis for 778,90 (Peak index 1), 964,08 (Peak index 2), 1085,87 (Peak index 3), 1112,07 (Peak index 4), 1408,01 (Peak index5) KeV (a) Peak area for measurement without sample (b) First RP3A-1 measurement (c) Second First RP3A-1 measurement (d) Third RP3A-1 measurement

Table S6. The amount of deviation between the peak areas obtained for RP3A-1 and the average of these areas.

Energy (KeV)	Sample-free measurement	RP3A-1_01	(%) Deviation	RP3A-1_02	(%) Deviation	RP3A-1_03	(%) Deviation	Average Peak Area
121,78	2315	2088	5%	1847	-7%	2053	3%	1996
344,28	2326	2379	7%	2289	3%	2289	3%	2227
778,90	1916	1764	-4%	1731	-6%	2018	10%	1837
964,08	3543	3600	6%	3118	-8%	3435	1%	3384
1085,87	3506	3182	-5%	3689	10%	3187	-5%	3353
1112,07	4222	3905	-3%	4213	5%	3955	-2%	4024
1408,01	8784	8566	-1%	8752	1%	8694	0%	8671

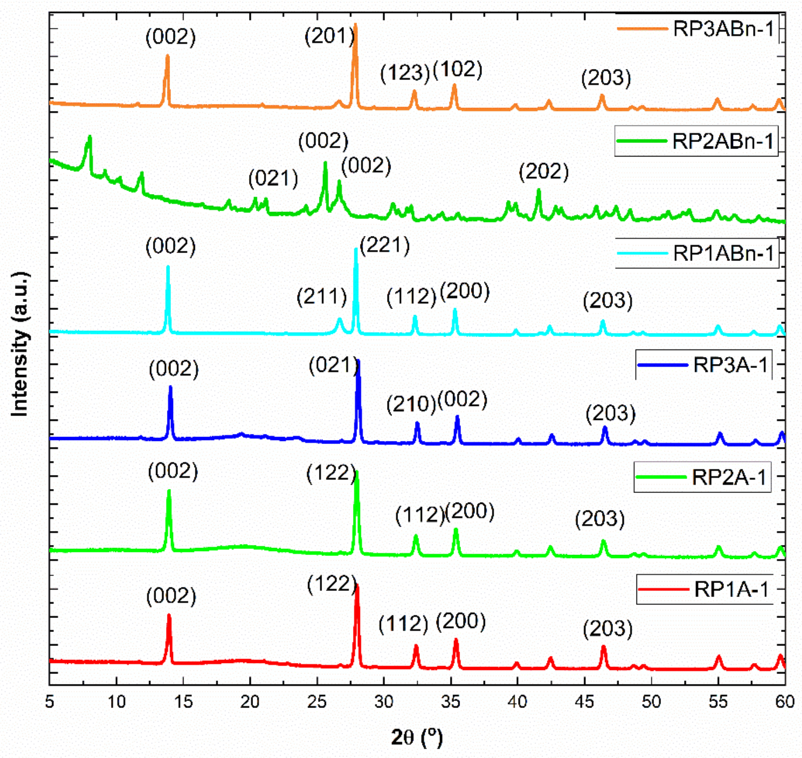

Figure S11. XRD graphs of RP1A-1, RP2A-1, RP3A-1, RP1ABN-1, RP2ABN-1 and RP3ABN-1 nanocomposites

Figure S11 shows the XRD diffraction patterns of selected nanocomposites. By inspecting the patterns, it can be seen that the materials are more crystalline in nature; this is an important point regarding the determination of phase integrity and stability. Although the pattern of the RP2ABN-1 sample exhibits an amorphous nature slightly, the fact that there are identical diffraction angles (2θ) and hkl values represented-to-listed also in Table S4 implies that the composites still have the same structure. This proves the successful inclusion of As_2O_3 and BN NPs into the PS-PEG matrix with the same density that gives a positive result regarding gamma radiation protection.
